# Cellular Identity Crisis: RD3 Loss Fuels Plasticity and Immune Silence in Progressive Neuroblastoma

**DOI:** 10.1002/advs.202519586

**Published:** 2026-01-31

**Authors:** Poorvi Subramanian, Sreenidhi Mohanvelu, Sheeja Aravindan, Afsana Parveen Jahir Hussain, Sivasubramani Narayanan, Sabir Salim, Loganayaki Periyasamy, Natarajan Aravindan

**Affiliations:** ^1^ Department of Physiological Sciences Oklahoma State University Stillwater Oklahoma USA; ^2^ OU Health Stephenson Cancer Center Oklahoma City Oklahoma USA

**Keywords:** cancer immunoediting, cellular plasticity, EMT, neuroblastoma, phenotypic switching, RD3, tumor immune evasion

## Abstract

Therapy‐resistant neuroblastoma (NB) reflects a lethal convergence of cellular plasticity and immune escape, yet the molecular drivers remain elusive. Here, we identify retinal degeneration protein 3 (RD3) as a master regulator of NB lineage fidelity and tumor immunogenicity. RD3 loss, induced by therapy, triggers a reprogramming cascade involving epithelial‐mesenchymal transition, pluripotency circuitry, and cancer stem cell enrichment. Mechanistically, RD3 binds to and regulates the promoter activity of SOX2, NANOG, and OCT3/4, as well as regulates the transcription of EMT factors. Concurrently, RD3 deficiency disrupts antigen presentation (MHC I/II, β2M), upregulates immune checkpoints (PDL1, CD276), and activates immune‐suppressive signaling (CD24, CD73, A2AR), fostering an immune‐silent microenvironment. RD3 restoration reverses these transitions, reinstating epithelial identity, differentiation, and immune surveillance. In‐vivo, RD3 modulates immune cell infiltration, activation, and tumor clearance. Mechanistically, RD3 governs a self‐reinforcing axis of cellular identity and immunoediting (by regulating T‐cell cytokine release, activation, and cytotoxic function), positioning it as a critical checkpoint in NB evolution. These findings establish RD3 as a dual‐function molecular switch and nominate RD3‐targeted strategies to re‐sensitize high‐risk NB to immunotherapy.

## Graphical abstract

Researchers discovered that therapy‐induced loss of RD3 protein in neuroblastoma triggers a dangerous shift: cancer cells become more stem‐like, invasive, and resistant to treatment while evading immune detection. RD3 loss suppresses antigen presentation and boosts immune checkpoints, creating an immune‐silent environment. Restoring RD3 reverses these changes, improving differentiation and immune response, offering a promising therapeutic strategy.

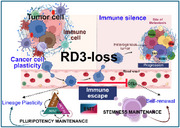

**Abbreviations**
A2AR: Adenosine A2A Receptor
AA: Amino acid
ALDH2: Aldehyde Dehydrogenase 2
ATCC: American Type Culture Collection
CALCB: Calcitonin related Peptide β
CD133: Cluster of Differentiation 133
CD206: Cluster of Differentiation 206
CD244.2: Cluster of Differentiation 244.2
CD39: Cluster of Differentiation 39
CD4: Cluster of Differentiation 4
CD44: Cluster of Differentiation 44
CD73: Cluster of Differentiation 73
CD8: Cluster of Differentiation 8`
CD86: Cluster of Differentiation 86
CD95: Cluster of Differentiation 95
COG: Children's Oncology Group
CSC: Cancer Stem Cells
CSF1: Colony Stimulating Factor 1
DMEM:F12: Dulbecco's Modified Eagle Medium/Ham's F‐12 Nutrient mixture
Dx: Diagnosis
Dx‐RD3^+/+^
: RD3‐expressing Dx cells
E/M: Epithelial/Mesenchymal hybrid state
ECM: Extracellular matrix
EDTA: Ethylenediaminetetraacetic acid
EMT: Epithelial Mesenchymal Transition
FBS: Fetal Bovine Serum
GITR: Glucocorticoid‐Induced Tumor Necrosis Factor Receptor
hPDGF: human Platelet‐derived Growth Factor
HR‐NB: High Risk Neuroblastoma
IACUC: Institutional Animal Care and Use Committees
ICAM1: Intercellular Adhesion Molecule 1
ICs: Immune checkpoint molécule
IHC: Immunohistochemistry
IL8: Interleukin 8
IMDM: Iscove's Modified Dulbecco's Medium
KLF4: Kruppel‐Like Factor 4
LIF: Leukemia Inhibitory Factor
MET: Mesenchymal Epithelial Transition
MHC I: Major Histocompatibility Class I
MHC II: Major Histocompatibility Class II
MMP9: Matrix Métalloprotéinase 9
MSDACs: Metastatic Site Derived Aggressive Cells
Multi‐ICF: Multiplex Immunocytofluorescence
Multi‐IHF: Multiplex Immunohistofluorescence
NB: Neuroblastoma
PD: Progressive disease
PDL1: Programmed Death Ligand 1
PD‐RD3^−/−^
: RD3‐deficient PD cells
PI3KR1: Phosphoinositide‐3‐kinase regulatory subunit 1
PTPN3: Protein Tyrosine Phosphatase Non‐Receptor Type 3
RD3: Retinal Degeneration 3 protein
RPMI: Roswell Park Memorial Institute
SMAD4: Mothers against decapentaplegic homolog 4
STING: Stimulator of Interferon Genes
T_c_
: T‐cells
TGFβ: Transforming growth factor β
TIME: Tumor Immune Evasion
TMA: Tissue Microarray
TME: Tumor microenvironment
T_u_
: Tumor cells
ZIC3: Zinc Finger Transcription Factor of Cerebellum 3

## 1 Introduction

**FIGURE 1:**
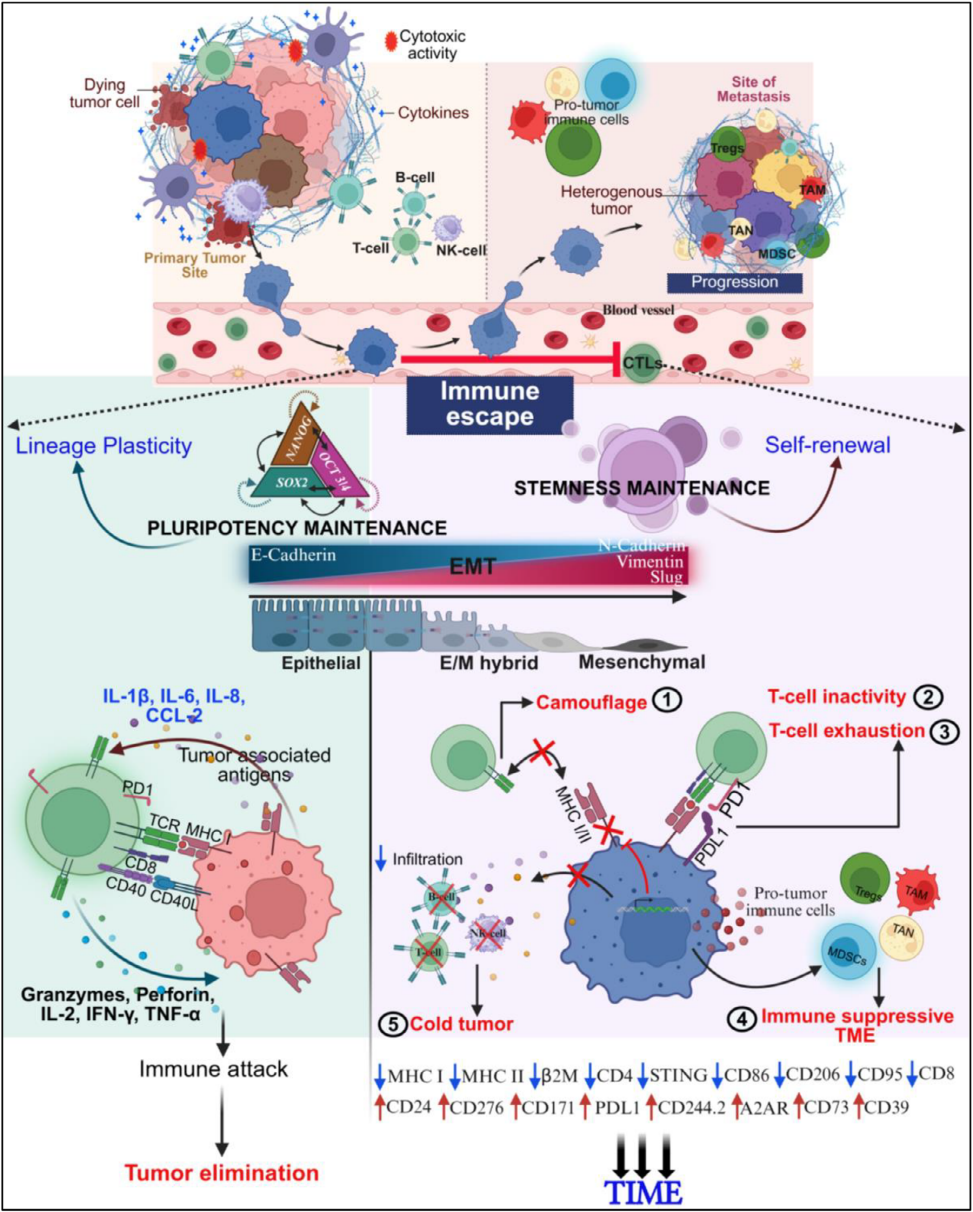
Schema illustrating the role of cellular plasticity in NB immune evasion. This schematic overview highlights how RD3‐loss orchestrates plasticity‐driven tumor immune editing. Therapy‐pressure acquired RD3‐loss prompts EMT induction in the primary tumor, which facilitates migratory and invasive behavior. Concurrently, T_u_ acquires stemness traits and maintains pluripotency, enabling lineage reprogramming and clonal expansion. These phenotypic transitions promote immune escape by downregulating antigen presentation molecules MHC I, MHC II, B2M, and upregulating immune checkpoint molecules PDL1, CD276, resulting in immune inactivation. Enhancing CD24 and GD2 promotes immune suppression by camouflage. The evolving tumors are characterized by reduced infiltration of effector T_c_ CD4, CD8; expansion of immune suppressive subsets Tregs, TAMs, and activation of adenosinergic pathway via CD39, A2AR, CD73. Imposingly, these changes transform the immune landscape from ‘hot’ state to an immune‐silent ‘cold’ state. ↑= upregulated; ↓= downregulated.

Cellular plasticity encompasses a broad spectrum of dynamic and adaptive reprogramming of cancer cells, triggering an identity crisis [1, 2]. This adaptability fosters passive enrichment of intra‐ and inter‐tumoral heterogeneity, shaping tumor evolutionary trajectory [2, 3]. This undeniably accelerates tumor progression and metastasis by driving tumor immune evasion (TIME), therapy resistance, and frequent recurrences within a rapidly decreasing timeframe [2, 4, 5]. Tumor‐mediated immune tolerance has long been an inherent feature of cancer biology, but recent advances have established it as a central paradigm in oncology. Malignant cancer cells have adapted and/ or respond to their environmental cues leading to successful evasion of host immune attack [6]. This critical process of TIME could be explained by the ‘three Cs model’: camouflage; coercion; cytoprotection [6]. In brief, the process of TIME involves effective disguise (plasticity) of tumor cells (T_u_) from circulating effector immune cells; immune editing and compensatory mechanisms such as defective immunological synapse and autophagy resistance [6, 7, 8, 9]. As a consequence, cancer cells limit the control of immune system over tumor and successfully evade immune surveillance [4, 5, 10]. One of the key processes contributing to immune escape is epithelial mesenchymal transition (EMT), a highly dynamic and dichotomous profile where‐in cells interconvert between epithelial‐and‐mesenchymal states [2, 11, 12]. This shape‐shifting cell‐state has been strongly associated with metastatic colonization and tumor progression [4, 5, 10, 11, 12, 13, 14]. Further, epithelial/mesenchymal (E/M) hybrid state driven plasticity is associated with unique immune‐modulatory effects within the tumor microenvironment (TME) involving both suppression and evasion [4, 5, 11, 14]. A full EMT is marked by decreased expression of antigen presenting molecules (Major Histocompatibility Complex; MHC Class I, II) and upregulation of immune‐neutralizing protein (Programmed death ligand 1; PDL1) preventing cancer cells to be recognized and lysed by T‐cells (T_c_, specifically the CD8^+^) [4, 5, 12, 15]. This adoptive immune feature of cancer cells without losing its plasticity contributes to EMT‐induced attenuation of CD8^+^ T_c_ activation. In addition, mesenchymal cell‐state promotes an immune‐suppressive TME through release of soluble factors (TGFβ, IL8, CSF1) and associated polarization of pro‐tumor immune cells (T_regs_ and tumor associated macrophages) [4, 5, 12]. Thus, T_u_ exhibiting sustained maintenance of E/M features are prone to develop anti‐tumor immune resistance and evasion (Figure 1). That being said, the molecular mechanisms and/or regulators governing plasticity driven TIME remain thus far unrealized. In this context, we aimed to investigate these processes in neuroblastoma (NB), a pediatric tumor marked by pronounced ‘undifferentiated cell‐state’ and immunologically ‘cold’ microenvironment.

NB is the most aggressive fetal malignancy of the sympathetic nervous system characterized by systemic metastasis, tumor evolution and relapses occurring with rapidly decreasing timeline [16, 17, 18, 19]. Despite the existence of extensive treatment modalities (induction therapy; consolidation therapy; maintenance therapy and; salvage therapy) NB persists, both locally and systemically, which contributes to poor quality of life and unacceptably low long term survival rates (<10%) in patients presented with high‐risk (HR), progressive stage‐4 disease [16, 17, 20, 21]. Each therapeutic regimen imposes selective pressure that drives tumor heterogeneity by promoting clonal expansion and selection of resistant sub‐clones [16, 18]. In parallel, NB cells exhibit adaptive and reversible lineage transformation which contributes to immune sculpting and adaptive resistance [19, 22]. In particular, residual and relapsed tumors exhibit distinct immunological profiles with altered immune cell infiltration, reduced Cytotoxic T_c_ activity and increased immune suppressive phenotype [22]. These hallmarks of aggressive and progressive cancer states are strongly attributed to NB cell's adoptive and reversible lineage transformation (cellular plasticity) and cold immune status [22]. Hence, there is an urgent requisite to define the mechanism and/or the driver of NB cellular plasticity driven immune repertoire. In this study, we investigated the impact and relevance of retinal degeneration protein 3 (RD3) in NB lineage transformation and cellular plasticity driven TIME.

RD3 is a small protein consisting of 195 amino‐acid (AA) initially identified for its expression in the retina. A genetic non‐sense mutation at position 319 of exon 3 (C→ T transition) produces a 36AA truncated protein that subsequently degrades, leading to loss of protein [23]. Our studies first revealed that the availability and abundance of RD3 is not restricted to retina, but constitutively expressed in all fetal and adult tissues [24]. With absolutely no functional mutations associated in NB, we observed a steady decline in its expression along NB evolution: from healthy tissues → ganglioneuroma → ganglio‐NB → NB [18, 25]. Further, we showed that RD3‐loss associated with advanced disease stage functionally mediates NB pathogenesis [25]. Crucially, our studies documented a de novo acquisition of RD3‐loss with therapy pressure in NB [18]. More importantly, our recent findings unveiled RD3's relevance in tumor immune surveillance and immune homing, and recognized that therapy pressure driven acquired RD3‐loss coordinates TIME in NB‐TME [22]. Precisely, RD3‐loss negatively impacts immune cell type composition, deterring homing and surveillance of CD4^+^ and CD8^+^ T_c_ [22]. Critically, we identified a model‐independent 27‐gene signature panel through which RD3 regulates TIME in NB [22]. Our findings imply a strong association of RD3‐loss with reduced infiltration and activation of T_c_, while conversely, it is correlated with enhanced survival of T_u_ [22, 25]. With such overwhelming relevance of RD3 in disease progression, functional significance in the aggressive metastatic disease evolution, acquired loss of RD3 with therapy pressure, and regulatory role of TIME in NB, herein we investigated the function of RD3 in lineage transformation and cancer cell plasticity, and its consequential TIME harmonization. With the comprehensive panel NB patient derived cell lines during disease at diagnosis (Dx), progressive disease (PD) that defy therapy coupled with six independent and controls matched RD3 reverse engineered in vitro and in vivo models, the outcomes of this study identified the difference in the composition and physiognomies of cancer stem cells (CSC) between disease at Dx and PD; function and mechanism of RD3 in regulating T_u_→CSC lineage transformation and coordinated NB cell plasticity; the significance of the RD3 driven plasticity in driving aggressive metastatic disease; mechanism how RD3 regulates T_u_ immunogenicity, and how RD3‐loss with therapy pressure dictates immune checkpoint and TIME and; unveiled novel insights into RD3‐driven NB plasticity‐immune evasion synergy. Collectively, outcomes from this study paves way for developing novel and effective therapeutic strategy that could counter therapy pressure driven cellular plasticity, and lineage transformation steered TIME in the deadly therapy defying progressive NB (Figure 1).

## 2 Materials and Methods

### 2.1 Cell Culture

All cell‐lines used in this study are well‐established, de‐identified, and their use in this study does not involve human subjects or new human samples, thus requiring no specific IRB approval for the cell line itself, adhering to our Institution's Policy. All experiments were performed in accordance with our institution's guidelines and regulations for the use of commercially available de‐identified human cells. Fourteen high‐risk (HR) human NB stage‐4 cell lines derived during diagnosis (CHLA‐15, RRID:CVCL_6594; CHLA‐42, RRID:CVCL_6603) and progressive disease (CHLA‐20, RRID:CVCL_6602; CHLA‐60, RRID:CVCL_6607; CHLA‐61, RRID:CVCL_AQ17; CHLA‐79, RRID:CVCL_6608; CHLA‐90, RRID:CVCL_6610; CHLA‐140, RRID:CVCL_6592; CHLA‐171, RRID:CVCL_6597; SMS‐LHN, RRID:CVCL_9539; COG‐N‐291, RRID:CVCL_AQ18; LA‐N‐6, RRID:CVCL_1363; NB‐Eb‐C1, RRID:CVCL_E218; and SK‐N‐FI, RRID:CVCL_1702) obtained from the children's oncology group (COG) NB cell line repository (March 14, 2016), Lubbock, TX were cultured and maintained in‐house as described in our earlier studies [18, 22]. In brief, NB cells were cultured in IMDM supplemented with 20% FBS, 4 mM L‐Glutamine, 5 µg mL^−1^ insulin, 5 µg mL^−1^ transferrin, 5 ng ml^−1^ selenous acid and 1% Pen‐strep (penicillin, 12 units/ml; streptomycin 12 µg/mL). LA‐N‐6 and SK‐N‐FI cells were cultured and maintained in RPMI‐1640 medium supplemented with 10%FBS and 1% Pen‐strip. All cell lines were contamination‐free and authenticated by COG and are available online (http://www.cogcell.org/clid.php). The commercially available human NB cell line SH‐SY5Y was obtained from ATCC (Manassas, VA, USA), cultured and maintained in DMEM:F12 media supplemented with 10% FBS and 1% Pen‐Strep as described in our earlier publications [23, 25, 26]. Immortalized human T_c_ was obtained from Applied Biological Materials Inc (Vancouver, Canada. CAT#T0086), cultured and maintained in PriGrow X series medium supplemented with 1% Pen‐strep (penicillin, 12 units/ml; streptomycin 12 µg/mL), 10% FBS and IL2 (20 ng/ml) supplemented every 3 days. For initial activation Immunocult human CD3/CD28 T_c_ activator (Stemcell Technologies, Vancouver, Canada) was added immediately after thawing according to manufacturer's instruction. For passage and all other experiments, the cells were detached using trypsin (0.05%) and EDTA (0.53 mM) (Corning, CA, USA), re‐suspended in complete media and incubated in 95% air/5% CO2 humidified chamber. Cell viability was performed using 0.4% trypan blue exclusion (Sigma, MO, USA) and cells were counted using the countess automated cell counter, (Invitrogen, MA, USA).

As part of our in vivo experiments T_u_ from primary (PT) and metastatic sites (MT1‐5) from SH‐SY5Y model were derived and maintained ex vivo as described in our earlier studies [27]. MT1‐5 refers to five different animals; each animal harbored multiple metastatic tumors, which are further designated as MT1‐1, MT1‐7 etc., indicating animal number and tumor number, respectively. In brief, tumor samples were thoroughly homogenized in cell dissociation buffer (DMEM:F12 media supplemented with 10% FBS, 1% Pen‐strep, 200U ml^−1^ Collagenase IV and 0.6U ml^−1^ Dispase). The homogenized tissue was incubated at 37°C for 2 h with intermittent vortexing for 1 min every 20 min. The cell suspension was filtered through a 70 µm filter, equal volume of Ficol‐Paque Plus was layered below and subjected to density gradient centrifugation to separate viable T_u_ from debris. Interface was collected carefully and washed once with thrice the volume of DMEM:F12 media. Cells were grown and maintained ex‐vivo in stem cell medium (DMEM:F12 with 1% N_2_ supplement, 20 ng ml^−1^ hPDGF, 100 ng/ml EGF, 1% Pen‐Strep) at 37°C, 5% CO_2_ humidified chamber.

### 2.2 Therapy Pressure Inflicted Acquisition of RD3‐Loss

To delineate whether the therapy pressure acquired RD3 loss is therapy (chemo‐drug) specific, as well to identify variances between chemo‐drugs, if any, three RD3‐expressing Dx systems (SH‐SY5Y, CHLA‐15 and CHLA‐42) were exposed to clinically relevant dose regimes (exposed to three cycles of treatment) individually for each drug and the resistant cells are examined for RD3 transcription (qPCR) at 24 h and protein levels (immunoblotting) at 36 and 48 h. Drugs clinically used in induction phase including vincristine (0.1 µM), doxorubicin (1 µM), cisplatin (10 µM); both in induction and consolidation phase, etoposide (10 µM); in maintenance phase, 13‐cis‐retinoic acid (10 µM); and in salvage therapy, docetaxel (0.01 µM) were utilized for the study.

### 2.3 RD3 reverse Engineering—Development of RD3 Restored Clones of PD‐RD3^−/−^ cells and RD3 KO Clones of Dx‐RD3^+/+^ cells

RD3 stable restoration in PD‐RD3^−/−^ cells, CHLA‐20, CHLA‐90, CHLA‐140 and, stable silencing of RD3 in Dx‐RD3^+/+^ cells, CHLA‐15, CHLA‐42, and SH‐SY5Y were carried out as described in our earlier studies [22, 25]. Consideration of these cell‐lines is in line with specific inclusion/exclusion (RD3 status, primary/metastatic, diagnosis/treatment, etc.) criteria required to define the function of RD3 in the cellular plasticity and the development of metastatic disease. Re‐expression of RD3 was achieved using RD3 (NM_183059) human tagged ORF Clone in pCMV6‐AC‐GFP (tGFP‐tagged, RD3 transcript variant 1, Origene, MD, USA). Stable silencing was performed using RD3 Mission shRNA (GCTGGAAGATGTCTGCGTTAA) in pLKO.1 – puro vector (TRCN0000116068, Sigma, St. Louis, MO, USA). Transfection was achieved using Neon Nxt electroporation transfection system with pulse voltage (1200 V), width (20 ms) and two pulses (Life Technologies, MA, USA); G418 (200 µg ml^−1^) and puromycin (3 µg ml^−1^) (Selleckchem, TX, USA) were used as mammalian selection markers to create stable RD3 restored and silenced clones, respectively. Modifications in RD3 levels in generated stable clones were validated by immunoblotting and immunofluorescence (vs. their respective parental controls). Further to define the role of RD3 in self‐renewal and pluripotency, we adopted two approaches (i) silenced SOX2, NANOG and OCT3/4 pluripotency regulators in highly aggressive NBP4 ex‐vivo clone (RD3^−p/−^ status). Transient transfection of SOX2 (TRCN0000355694), NANOG (TRCN000004885) and OCT3/4 (TRCN0000004881) (Sigma, St. Louis, MO, USA) was carried out by neon electroporation transfection system following the above‐mentioned parameters. (ii) Assessed the expression levels of these markers (immunoblotting) after restoration of RD3.

### 2.4 RD3 Elisa

Therapy pressure‐associated RD3 status comparing the levels between cells derived during Dx and those derived during PD that defy current clinical therapy in HR‐NB patients (total n = 14) was determined using SimpleStep ELISA custom ELISA kit (Abcam, MA, USA) following standard manufacturer's instructions. In brief, 50 µl protein lysate and 50 µl of RD3‐antibody cocktail were added to each well and incubated (4°C for 1 h). The wells were washed, followed by the addition of detection reagent and incubated in the dark for 15 min. Stop solution was added at the end of incubation, and absorbance was read at 450 nm using Synergy II microplate reader (Biotek Instruments, Vermont, USA). Cell‐specific RD3 expression was quantified and compared using Two‐way ANOVA with Dunnett post‐hoc correction. All samples were assayed in duplicates and quantified with two different protein inputs (10 and 5 µg ml^−1^).

### 2.5 RD3 pull‐down and SOX2, NANOG and OCT3/4 Co‐Immunoprecipitation

Co‐immunoprecipitation (Co‐IP) was performed using the Pierce co‐IP kit (Thermo Fisher Scientific, MA) following the standard manufacturer's instructions. In brief, cell lysates prepared using IP lysis buffer supplemented additionally with protease inhibitor cocktail and phosphatase inhibitor cocktail were cleared of debris by centrifugation at 13000 g for 10 min at 4°C. Desired concentrations of protein (quantified with BCA assay) were pre‐cleared using control agarose resin for 60 min at 4°C to minimize non‐specific binding. Custom‐archived rabbit polyclonal anti‐human RD3 antibody [23] was covalently immobilized onto the aminolink resin in spin columns using 1X coupling buffer and reducing agent sodium cyanoborohydride for 2 h at 4°C with gentle rocking, followed by quenching using quenching buffer and extensive washing to remove unbound antibody from resin. Subsequently, 100 µg of pre‐cleared lysates (∼50 µl/sample) were incubated with RD3 antibody‐coupled resin for 2 h at 4°C with gentle mixing. Following incubation, columns were centrifuged at 1,000 × g for 1 min, and the flow‐through was retained for further analysis. The resin was washed once with IP lysis buffer to remove unbound proteins. Bound complexes were eluted sequentially twice with 50 µl elution buffer. RD3 pull‐down was confirmed with western blotting, and the co‐immunoprecipitation of SOX2, OCT‐3/4, and NANOG was analyzed by immunoblotting using respective antibodies (Cell Ssignaling Technology, MA). Input (whole cell lysate) and unbound fraction (flow‐through) were used as controls.

### 2.6 ChIP‐qPCR

ChIP was performed using SimpleChIP Enzyamtic Chromatin IP kit (Cell signaling technologies, MA, USA) following standard manufacturer's instructions. In brief, cells cross‐linked using 1% formaldehyde for 10 min at RT and quenched with glycine for 5 min were washed twice with ice‐cold PBS. Nuclei were isolated using buffer A supplemented with PIC and DTT and resuspended in ice‐cold buffer B supplemented with DTT. Chromatin was digested using 0.25 µl micrococcal nuclease/reaction (20 min at 37°C) with intermittent mixing to obtain optimal DNA fragments (∼150–900 bp). Digestion reaction was stopped by the addition of 0.5 M EDTA, and the nuclear pellet was resuspended in 100 µl ChIP buffer. Samples were sonicated (Misonix XL‐2000 series, Qsonica) at amplitude 2, 4 cycles of 5s on/35s off, with samples kept on ice between pulses. Lysates were clarified by centrifugation (9400 g for 10 mins, 4°C), purified with RNase A and Proteinase K, and then quantified. 20 µg of purified digested chromatin was diluted in ChIP buffer (supplemented with PIC) and incubated with RD3 antibody overnight at 4°C. ChIP grade protein G magnetic beads were added to the IP reaction and incubated for 2 hrs at 4°C. Beads were washed thrice sequentially with low‐salt and once with high‐salt ChIP buffers. Chromatin was eluted by incubating with 150 µl of ChIP elution buffer at 65°C, 30 min with gentle mixing. Cross‐links were reversed by incubating the elutes at 65°C, 30 min after adding NaCl and proteinase K. DNA was purified using spin columns and was analyzed by qPCR using primers specific for *SOX2* (F–CGCTGATTGGTCGCTAGAAAC; R‐ CCCCTTTTGCAAACACTCTC), *OCT3/4* (F–TGAACTGTGGTGGAGAGTGC; R–AGGAAGGGCTAGGACGAGAG), and *NANOG* (F‐ GTCTTTAGATCAGAGGATGCCCC; R–CTACCCACCCCCTATTCTCCCA) promoter regions.

### 2.7 Immunoblotting

Cellular protein extraction, quantitation, and immunoblotting were performed as described earlier [28]. In this study, to validate RD3 stably re‐expressed and silenced systems, blots were incubated with our custom‐synthesized and well‐characterized anti‐rabbit RD3 Polyclonal antibody (Human reactivity; Abclonal, MA, USA). To assess RD3's role in cellular plasticity, protein‐transferred membranes were incubated with rabbit polyclonal anti‐human E‐Cadherin, SOX2, RON‐β (Santa Cruz Biotechnology Inc., TX, USA), N‐Cadherin, NANOG, OCT3/4, (Aviva Systems Biology corp., CA, USA), CD54 (Cell Signaling Technology, MA, USA); mouse monoclonal anti‐human NOTCH‐1 (Thermo Fisher Scientific, MA, USA), GAP‐43, NF‐H, β‐3‐Tubulin, GFAP (Santa Cruz Biotechnology Inc., TX, USA), MMP‐9 (Biolegend Inc., CA, USA), Vimentin, Slug, MMP‐2 (Abcam, Waltham, MA). All membranes were developed using appropriate anti‐mouse/anti‐rabbit secondary antibodies (Invitrogen, MA, USA). To elucidate RD3‐loss induced cellular plasticity impact on TIME, we quantified a panel of immune surveillance and response proteins, rabbit polyclonal anti‐human CD276, PDL1, HLA A/B, HLADPB1, β2M, CD171, GD2, and CD24 (Abclonal Technologies, MA, USA) in our 12 RD3 systems. To assess whether NFκB mediates RD3‐regulated PDL‐1 expression, cytoplasmic, and nuclear lysates [26] from two independent RD3‐deficient (CHLA‐20 and CHLA‐90) systems treated with and without Bay 11‐7082 (IκBα phosphorylation blocker, 10 µM Calbiochem, San Diego, CA, USA) and harvested after 24 h were used. For this, membranes were probed with rabbit polyclonal p65 (Abclonal Technologies, MA, USA) and rabbit polyclonal PDL1 (Cell Signaling Technologies, MA, USA). Mouse monoclonal β‐Actin (Sigma–Aldrich, MO, USA), rabbit monoclonal GAPDH, or rabbit monoclonal α‐tubulin (Abclonal Technologies, MA, USA) served as loading controls. Quantitation of blots was performed using Quantity One gel analysis software (Bio‐Rad). GAPDH, α‐tubulin, or β‐actin normalized values were compared between groups and statistical tests involving t‐tests and/or ANOVA with post hoc correction were performed in GraphPad prism.

### 2.8 Immunophenotyping and Characterization of Cancer Stem Cell Composition

Association of therapy pressure, metastasis, and RD3 loss in the modification of CSC composition were assessed in a panel of (n = 10) HR‐NB patient derived cell lines and RD3 reverse engineered systems (n = 4) utilizing sequential exclusion inclusion flow sorting [29]. Briefly, cells were pelleted, washed and re‐resuspended in ALDEFLUOR buffer (Stem Cell Technologies, MA, USA) and sorted using defined stem cell phenotypes with appropriate controls using a BD FACSAria Fusion (BD Biosciences, CA, USA). We adopted a sequential exclusion → inclusion criteria to isolate human NB CSCs. Unstained, single stained (CD133^+^PE, CD114^+^APC, CD117^+^APCVio770), ALDEFLUOR^+^DEAB^−^, ALDEFLUOR^+^DEAB^+^, fluorescence minus One (FMOs; ALDEFLUOR^+^CD133^+^CD114^+^, ALDEFLUOR^+^CD114^+^CD117^+^, ALDEFLUOR^+^CD133^+^CD117^+^) were used as compensation controls to set up appropriate gates. Forward scatter vs. side scatter was used to eliminate debris and doublets. Viable cell population was included using 7‐AAD staining (Biolegend, CA, USA). Unstained and compensation controls were used to correct autofluorescence and spectral overlap between fluorophores. Data acquisition was carried out for 100,000 cells/sample. NB‐CSCs were screened and isolated for double‐positive population (ALDH^+^CD133^+^CD114^+^, ALDH^+^CD114^+^CD117^+^ and, ALDH^+^CD133^+^CD117^+^) and triple‐positive cell population with ALDH^+^CD133^+^CD117^+^CD114^+^ phenotype. NB‐CSCs were collected and maintained in stemline neural stem cell expansion media (Sigma–Aldrich, MO, USA) without FBS, complemented with 100 ng/ml epidermal growth factor (EGF) and 10 ng/ml fibroblast growth factor (FGF) (Biolegend, CA, USA). For immune markers, cells were stained with PE‐anti HLA A/B/C, APC‐anti‐β2M, and FITC‐anti‐PDL1 (Biolegend, CA, USA). Unstained, single‐stained, FMOs controls were used for compensation and to set up gates. Data from FACSAria were further processed and analyzed using FlowJo software. The percentage of single, double, and triple positive population was calculated, and statistical analysis was performed using GraphPad prism software.

### 2.9 Clonal Expansion by BrdU Incorporation Assay

Parental NB cell lines from Dx (n = 2, CHLA‐15 and CHLA‐42), PD (n = 8, CHLA‐20, CHLA‐61, CHLA‐79, CHLA‐90, CHLA‐140, CHLA‐171, NB‐Eb‐C1 and SK‐N‐FI), reverse engineered clones of RD3 stable restored CHLA‐20, CHLA‐90, CHLA‐140, and silenced CHLA‐15, CHLA‐42, SH‐SY5Y were treated with 1 µM bromodeoxyuridine (BrdU) for 2 h. BrdU labeling and detection in all cells were performed as described earlier [30]. Briefly, BrdU labeled cells were then washed and fixed in 4% paraformaldehyde supplemented with sucrose. Fixed cells were sequentially treated with 0.1% triton‐x‐100 buffer and 2N HCl with intermittent washing with PBS. The cells were blocked with 0.1% BSA in PBS, incubated with anti‐mouse BrdU Alexa Fluor (AF) 488 fluorescent tagged antibody (Santa cruz biotechnology Inc, TX, USA) for 1 h at 37°C. The assay wells were washed and counterstained with 4',6‐diamidino‐2‐phenylindole (DAPI) for nuclear detection. Images of BrdU‐incorporated cells were captured in 10 different fields using EVOS M5000 imaging system for Fluorescence and Transmitted Light Applications at 20X magnification (Thermo Fisher Scientific, Waltham, MA, USA).

### 2.10 Phase Contrast and Transmission Electron Microscopy (TEM)

To investigate RD3‐dependent phenotypic and ultrastructural alterations, we adopted a dual imaging strategy comprising (i) phase contrast imaging of fixed cells for assessing changes in cellular morphometric physiognomies and (ii) TEM for the visualization of ultrastructural modifications. For this, first, we screened our twelve ex vivo systems, including Dx clones with inherent RD3 expression; PD clones with therapy pressure acquired RD3 loss; and their RD3‐reverse engineered (RD3 muted Dx clones, RD3 reinstated PD clones) counterparts. Under 80% confluence of these clones cultured in 60 mm plates and maintained under standard culture conditions (95% air, 5% CO_2_) were fixed with 3:1 methanol: acetic acid solution for 1 min to preserve its native morphology. Fixed cells were stained with crystal violet, and the phase contrast images at 20X magnification were acquired with constant exposure time, gain, and contrast settings. Representative fields were selected based on cell distribution and absence of debris and/or artifacts, if any. Second, TEM was performed for high‐resolution morphometric profiling in RD3^+/+^ and RD3^−/−^ systems. In brief, cells were washed with PBS and fixed in 2.5% glutaraldehyde in 0.1 M sodium cacodylate buffer (pH 7.0) for 2 h, and they were washed thrice for 15 min each followed by post‐fixation with 1% osmium tetraoxide for an hour. Samples were dehydrated through increasing concentrations of (50%, 70%, 90%, 95% and thrice in 100%) ethanol, each for 15 min. Dehydrated specimens were washed thrice in propylene oxide and infiltrated overnight in a 1:1 propylene oxide‐Poly/bed resin. Final embedding and polymerization were performed for 48 h at 60°C in 100% Poly/bed resin. Ultrathin sections (60 nm) were obtained using ultramicrotome equipped with diamond knife and mounted on copper grids. Sections were stained sequentially with uranyl acetate and lead citrate to enhance contrast prior to imaging. Images were taken using JOEL JEM‐2100 with Bruker EDS at 2000x magnification. Digital microphotographs were acquired and stitched in ImageJ. Cells selected randomly across multiple fields were examined per group to confirm consistency of morphological features; representative pictures were shown in the manuscript.

### 2.11 Tumor Cell Migration, Invasion, and Tumorosphere Growth

Cell migration and invasion assays were performed as described in our earlier studies [25]. Alterations in migration and invasive potential in response to RD3 status were assessed in a panel of ten NB ex‐vivo systems using trans‐well migration assay under proliferation‐controlled (mitomycin C, 10 µg ml^−1^, Sigma, MO, USA) conditions. For invasion assays, we coated inserts with 50% matrigel as discussed in our earlier studies [18, 25]. In brief, 1*10^6^ cells were seeded in the inserts and 24 h post incubation, cells that invaded through the pores were fixed in 3:1 methanol:acetic acid and stained with 0.1% Crystal violet. Images were captured in 0.8X and quantified using ImageJ. Group‐wise comparisons for both the T_u_ migration and invasion was performed and plotted in GraphPad prism (mean ± SEM) using ANOVA with Bonferroni's post hoc correction. p<0.05 was considered statistically significant.

Limited dilution tumorosphere assay (LDTA) was used to investigate RD3‐loss dependent tumorosphere formation (and/or cell differentiation) and cellular migrative capabilities as discussed in our earlier studies [30, 31]. For this, NB patient derived ex‐vivo clones were utilized, where Dx cell lines with inherent RD3 availability served as baseline control. In brief, cells were plated in triplicate on 96‐well culture plates and stained with 1,1′‐Dioctadecyl‐3,3,3′,3′‐Tetramethylindocarbocyanine Perchlorate (DiI; DiIC_18_(3), Life Technologies, Grand Island, NY), an orange‐red fluorescent dye and a long‐term tracer for neuronal cells. High content real‐time live cell fluorescent imaging every 20 min for a period of 42 h was performed in Operetta (Perkin Elmer, Inc., Waltham, MA) under a growth‐controlled environment. Sequential images were reconstructed in Harmony (Perkin Elmer), and a time‐lapsed video was obtained. Total distance travelled, and tumorosphere size were analyzed in our custom‐designed cell tracking computer tool that aids time‐lapse image analysis. Formation and growth of tumorosphere was calculated using the equation area = πabc, where ‘a’ is the semi‐major axis, ‘b’ is the semi‐minor axis, and ‘c’ is the measured absolute orientation. Cellular movement (total distance moved by the cell) was calculated using the formula, distance = √[(x2‐x1)^2^+(y2‐y1)^2^] where the x1 and y1 are the coordinates for particular cell at time point A and the x2, y2 are coordinates for the same cell at time point B. Mean distance travelled by each cell line was computed from the cumulative distance travelled by 50–500 individual cells over a period of time. Cellular motility and tumorosphere formation between groups were analyzed using GraphPad prism.

### 2.12 High Content Multiplex Confocal Immunocytofluorescence

We examined the cellular localization and expression of RD3 in 6 reverse engineered (CHLA‐15 RD3^−/−^, CHLA‐42 RD3^−/−^, SH‐SY5Y RD3^−/−^, CHLA‐20 RD3^+/+^, CHLA‐90 RD3^+/+^, CHLA‐140 RD3^+/+^) and corresponding parental systems (CHLA‐15, CHLA‐42, SH‐SY5Y, CHLA‐20, CHLA‐90, CHLA‐140). Further, the function of RD3 in altering pluripotency maintenance, lineage transformation, EMT, and metastatic potential was investigated using these reverse‐engineered clones (n = 6) with their matched parental controls (n = 6) using multiplex immunocytofluorescence (multi‐ICF). Multi‐ICF was performed manually following Novus biologicals multicolor ICC/IF standard protocol (Novus biologicals, Centennial, CO, USA). Twenty‐four hours post‐seeding, cells were fixed in 4% paraformaldehyde (10 min), permeabilized (0.25% Triton‐X‐100 buffer for 10 min), and blocked (1% BSA for 1 h) with extensive PBS washing between each step. Cells were incubated with human RD3‐AF488 conjugated antibody for 1h at 4°C. For assessing RD3‐dependent modifications in cell behavior, labeling on cells were performed using AF488 conjugated N‐Cadherin (N‐CAD), SOX2, GAP‐43, and NF‐H; AF546 labeled E‐Cadherin (E‐CAD), RON‐β, and MMP‐9; AF647 tagged Vimentin, NOTCH‐1, ALDH2, NANOG, β‐3‐Tubulin, GFAP, OCT3/4 and; CD133‐PE. Post‐labeling, cells were washed, and nuclei were counter‐stained using DAPI. Images were captured in EVOS M5000 imaging system for Fluorescence and Transmitted Light Applications (Thermo Fisher Scientific, Waltham, MA, USA). Cell detection was performed using DAPI followed by flour specific imaging. Post image fine‐tuning, including contrast and brightness for each channel was performed using EVOS M5000 integrated image correction software.

### 2.13 Coculture (CoC) and Cytokine Secretion Assay

To assess tumor intrinsic RD3‐dependent regulation of T_c_ activation and its effector function, we co‐cultured Tc with one RD3‐expressing parental (CHLA‐15 RD3^+/+^) T_u_, one RD3‐deficient parental (CHLA‐90 RD3^−/−^) T_u_, and their stably RD3 reverse‐engineered counterparts, CHLA‐15 RD3^−/−^ and CHLA‐90 RD3^+/+^. Prior to establishing CoC, T_u_ were labeled with CFSE (5 µM; Cell trace CFSE cell proliferation kit, Molecular probes, Waltham, MA, USA) and T_c_ with Tag‐It Violet (5 µM; Proliferation and cell tracking dye, Biolegend, San Diego, CA, USA) for 20 min at room temperature (RT) in the dark. Cells were washed with PBS to remove excess dye. CoC were established using matrigel at an effector‐to‐target ratio of 1:3 (T_c_:T_u_) and maintained under standard culture conditions (37°C, 5%CO_2_).

Cytokine profiling provides functional insight into T_c—_T_u_ interactions beyond surface expression. TNFα and IFNγ are hallmark effector cytokines of activated T_c_ and key mediators of anti‐tumor immunity. Their measure in T_c_ reflects activation status and effector function: TNFα modulates the TME and promotes apoptosis; IFNγ enhances antigen presentation and drives cytotoxic response. In contrast, the measure of cytokines TNFα and IFNγ in T_u_ suggests adaptive signaling to immune pressure. To facilitate intracellular cytokine staining, BrefeldinA (5 µg ml^−1^, Biolegend, San Diego, CA, USA) was added 2 h post CoC. Following CoC for 6 h, cells were harvested, washed with FACS buffer (PBS without Ca^2+^/Mg^2+^ supplemented with 2%FBS), and fixed using Cytofast Fix/Perm solution (Biolegend, San Diego, CA, USA) for 20 min at 4°C. After washing with Cytofast Perm/Wash solution (Biolegend, San Diego, CA, USA), intracellular staining was performed for TNFα (clone MAb11) and IFNγ (clone 4S.B3) (Biolegend, San Diego, CA, USA) in Cytofast Perm/ Wash solution for 30 min at 4°C. Samples were analyzed on FACSAria and data were processed using FlowJo software (v10). Unstained, single‐stained, and FMO controls (CFSE, Tag‐It‐Vio, TNFα, IFNγ) were appropriately used to set up compensation and minimize spectral overlap. Forward scatter vs. side scatter was used to eliminate debris and doublets. T_c_ (Tag‐It‐Vio^+^) and T_u_ separately (CFSE^+^) were identified and gated separately. Sequentially, cytokine positive fractions from both T_c_ and T_u_ were sorted using a 2‐way purity sort to obtain CFSE^+^TNFα^+^, CFSE^+^IFNγ^+^, Tag‐It‐Vio^+^TNFα^+^, Tag‐It‐Vio^+^IFNγ^+^ pure populations.

### 2.14 Tumor Reactivity Assay

To identify NB‐specific or reactive T_c_ that become activated upon recognizing tumor‐specific antigens, we established T_u_‐T_c_ CoC setup as described earlier with T_u_ embedded in matrigel domes and maintained for 12 h. To assess the functional activation of T_c_, we measured the key functional markers CD107a, a degranulation marker that indicates cytotoxic activity of T_c_, and CD137, a co‐stimulatory molecule expressed upon antigen‐specific activation. Cells were harvested from Matrigel using brief dispase digestion and washed once with FACS buffer. Surface staining for T_c_ activation markers CD107a (clone H4A3) and CD137 (clone S18014C) (Biolegend, San Diego, CA, USA) was performed along with appropriate compensation controls and viability dye. Data was acquired on FACSAria and analyzed using FlowJo software. The gates for sorting were set up as discussed in the earlier experiment until the selection of pure T_u_ and T_c_ populations. Within the T_c_ gate, we selectively gated for CD107a^+^ and CD137^+^ T_c_.

### 2.15 Tumor Killing Assay and Imaging

Tumor killing assay is a measure of direct anti‐tumor immune efficacy and is a measure of cytotoxic capacity of T_c_ against tumor targets (T_u_ elimination). To assess whether RD3‐loss influences susceptibility to immune‐mediated lysis and tumor clearance, we established CoC models as described earlier. In brief, T_u_ was labeled with CellTrace Far Red dye and T_c_ were labeled with Tag‐It‐Violet for 20 min at RT. To detect T_c_mediated apoptosis, cells were incubated with cell membrane‐permeable fluorogenic Caspase‐3 substrate (NucView488 Caspase‐3 assay kit, Biotium, Fremont, CA, USA) according to manufacturer's instruction. After 48 h, cells were harvested and processed using FACSAria, and data were analyzed using FlowJo software. Immunofluorescence images were captured in EVOS M5000 imaging system for Fluorescence and Transmitted Light Applications (Thermo Fisher Scientific, Waltham, MA, USA). T_u_ detection was performed using CellTrace Far Red (Cy5 filter), and T_c_ were detected using Tag‐It‐Violet (DAPI filter). Apoptotic cells were identified by NucView488 fluorescence and captured at 100x magnification. Post image fine‐tuning, including contrast and brightness for each channel was performed using EVOS M5000 integrated image correction software.

### 2.16 qPCR

We used real‐time qPCR to investigate therapy‐pressure driven RD3‐transcription (RD3) and RD3‐driven transcriptional modification of EMT transcription factors (*TWIST, SNAIL and SLUG*), pluripotency maintaining core circuit (*SOX2, OCT3/4, NANOG*), stemness (*LIF, ZIC3, NKX3.2)*, differentiation *(KLF4, PTPN3)*, plasticity and self‐renewal capabilities *(PI3KR1, SMAD4, LIN28, CALCB)* in primary and metastatic site derived clones as described in our earlier studies [32]. RNA was isolated using RNA STAT‐60 following manufacturer's instructions, reverse transcribed to cDNA and amplified using specific primers. We used β‐actin as an internal control. The ^ΔΔ^Ct values were calculated by normalizing the gene expression levels to β‐actin. The relative fold expression levels of genes were shown as fold change over parental SH‐SY5Y derived tumors. Group‐wise comparisons were performed using two‐way‐ANOVA with Tukey's post‐hoc correction in GraphPad prism.

### 2.17 In‐Vivo Mouse Models of Primary, High‐Risk Metastatic, and Pseudo‐Metastatic Disease

All animal experiments conformed to American Physiological Society standards for animal care and were conducted following guidelines from the National Research Council. All the protocols used were approved by the Oklahoma State University Institutional Animal Care and Use Committees (IACUC‐23‐42) and the University of Oklahoma Health Science Center (IACUC 20‐012‐CI). All animal studies complied with institutional guidelines on handling laboratory animals as well as all appropriate state and federal regulations. NB xenograft, metastatic disease development, tumor growth, and dissemination were assessed as per our earlier studies [18, 25]. For this study, seven‐week‐old mice homozygous for Foxn1^nu^ (Jackson Laboratory, Bar Harbor, ME) received SC injections of 1x10^6^ RD3‐expressing parental SH‐SY5Y and RD3‐null parental CHLA‐20 cells suspended in Matrigel (Corning, NY, USA) into their right flank. Animals were observed for xenograft and/or aggressive metastatic disease development for extended periods of time (15‐60 days). Tumor growth, regression, and dissemination to distant sites were investigated by tumor volume measurements. For assessing the function of RD3‐loss in metastatic disease, pseudo‐metastatic mouse models were developed. For this four‐week‐old homozygous Foxn1^nu^ (Jackson Laboratory, Bar Harbor, ME) mice received tail vein injections of 1x10^6^ RD3‐null parental CHLA‐20, CHLA‐90, and RD3‐expressing parental CHLA‐42 cells suspended in saline (0.89%). Parallel experiments with RD3 stably restored CHLA‐20 RD3^+/+^, CHLA‐90 RD3^+/+^ cells were performed. Animals were monitored for metastasis for a period of 90 days, and at the end of the experiment, all animals were euthanized by CO_2_ asphyxiation and the tumors harvested from primary and metastatic sites were harvested and prepared as single cell suspensions; flash frozen or fixed for downstream endpoint analysis.

### 2.18 Tissue Microarray (TMA) and Quantitative Immunohistochemistry (IHC)

All TMA construction, IHC staining, and quantification were performed as discussed in our earlier studies [27, 32]. IHC staining for SOX2 (Santa Cruz Biotechnology Inc., TX, USA), NANOG, and OCT3/4 (Aviva systems biology corp., CA, USA) was performed using automated Leica Bond Rx system at OU Health Stephenson Cancer Center—Tissue Pathology Core. The slides were digitally scanned using Aperio Scanscope (Aperio Technologies Inc., CA, USA) slide scanner and analyzed using spectrum software. Total, cytoplasmic, nuclear‐ stain intensity counts were quantified in precisely annotated cores within the TMA.

### 2.19 TIME‐Specific Multiplex Fluorescent Immunohistochemistry

To unequivocally define RD3's regulation in plasticity‐driven NB‐TIME, multiplex fluorescent immunohistochemistry studies were performed on developed tumors in RD3^−/−^, xenograft, and pseudometastatic models. This was performed in the Tissue Pathology Core of Stephenson Cancer Center (SCC) following standard protocols. In brief, tumors harvested from primary and pseudometastatic models were fixed, paraffin‐embedded, sectioned, and subjected to multi‐fluorescent IHC. Automated multi‐fluorescent IHC was performed on the Leica Bond RX as described earlier [22]. In this study, we utilized rabbit monoclonal anti‐CD4, CD8 (Abcam, Waltham, MA), CD73/NT5E (D7F9A), CD86, STING (Cell Signaling Technology Inc., MA, USA), GITR (CAL61) (Abcam, MA, USA), CD206 (LS Bio vector laboratories Inc., CA, USA), CD44 (Santa Cruz, Biotechnology Inc., TX, USA), mouse monoclonal CD95, CD244.2 (2B4 B6 Alloantigen) (Biolegend Inc., CA, USA). The nucleus was counterstained with Spectral DAPI, and the slides were mounted with Prolong Gold antifade reagent (Invitrogen) and scanned using Zeiss Axioscan.Z1 with calibre7, a high‐throughput imaging system to digitize slides with high resolution.

Identification of tumor infiltrating lymphocytes was achieved by utilizing specific inclusion/exclusion criteria including: CD4^+^CD206^+^CD44^−^CD86^−^ (naïve CD4^+^ T_c_), CD8^+^CD206^+^CD44^−^CD86^−^ (naïve cytotoxic T_c_), CD4^+^CD73^+^ (suppressive T‐helper cells), CD8^+^CD73^+^ (suppressive cytotoxic T‐ cells), CD4^+^CD206^+^CD44^+^CD86^+^ (activated helper T_c_), CD8^+^CD206^+^CD44^+^CD86^+^ (activated CD8^+^ T_c_), CD4^+^GITR^+^ (proliferating CD4^+^ T_c_), CD8^+^GITR^+^ (proliferating CD8^+^ T_c_), CD4^−^CD8^−^CD206^+^ (Tumor associated macrophages), CD4^−^CD8^−^CD244.2^+^ (NK cells). Image analysis was performed using multiplex‐IF module of the Qupath bioimage analysis software for precise identification and quantification of multiple fluorescent signals for each specimen [33]. Regions of interest on the specimen were carefully annotated to eliminate tissue deformities, artefacts, adjacent normal tissue and voids. DAPI staining was used as the cell detection channel for nuclear detection and segmentation with pixel resolution size at 0.1 µm. Nuclear parameters, including background radius and median filter radius, were configured to default settings of 8 µm and 0, respectively, to optimally detect nucleus within the annotated tissue. Noise reduction was applied using a sigma of 1.5 µm and nuclear size ranging between 10µm^2^ and 80µm^2^. Likewise, marker specific intensity threshold parameters were optimized for each immune marker to distinguish weak (+1), moderate (+2), and strong (+3) positive expression levels. For each immune marker, a single measurement classifier was assigned to precisely detect positive cells. The outputs from the whole image analysis were represented as average percent positive cells, with statistical significance determined using ANOVA.

### 2.20 Statistical Analysis

All statistical analysis were performed using GraphPad Prism (v10). Technical replicates were averaged to obtain a single value per biological replicate. When necessary, data were log‐transformed, and qPCR analysis was performed on ΔCt values. To compute the difference between two groups, we used one‐tailed/ two‐tailed unpaired t‐tests, and comparisons involving multiple groups were evaluated using one‐way or two‐way ANOVA with appropriate post‐hoc corrections Tukey, Sidak, Dunnett or Bonferroni. Statistical significance was set at P = 0.05. Data are presented as mean ± SEM, with exact *p* values reported for each test. Figure legends specify sample size (n), statistical test used, *p* values, and meaning of all significance symbols used.

## 3 Results

### 3.1 RD3 Diminution with Therapy Pressure Fosters Lineage Transformation, Stemness Maintenance, CSC Composition, and Propels NB Progression

**FIGURE 2:**
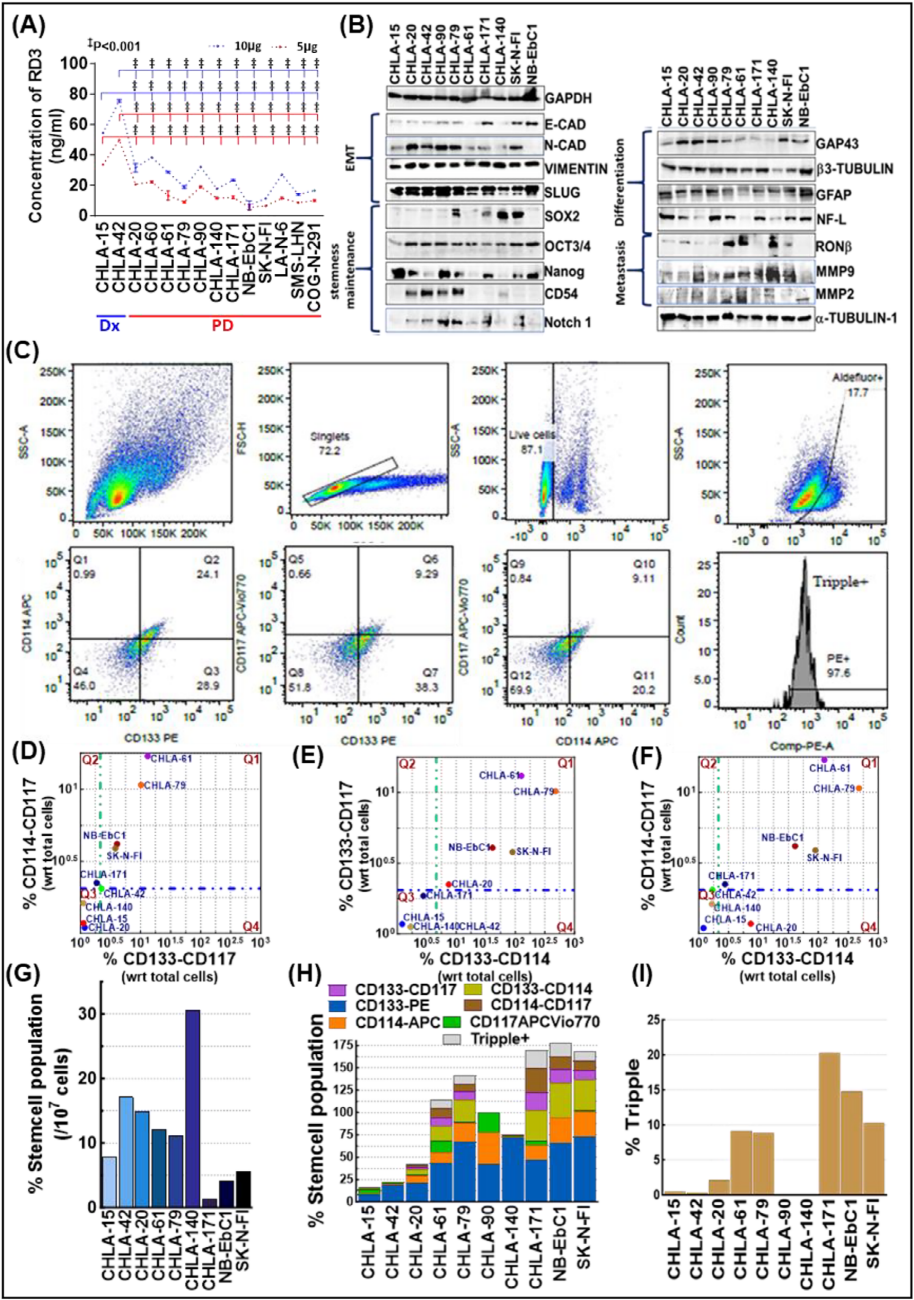
Clinical therapy pressure acquired RD3 loss associates with stemness acquisition and CSC enrichment in NB (A) ELISA showing reduced RD3 expression in a panel of patient derived post IMCT *ex vivo* systems when compared with therapy‐naïve NB. Data represent mean ± SD; statistical analysis was performed using two‐way ANOVA with Dunnett's correction (p<0.0001) (B) immunoblots showing RD3‐loss associated rearrangements of key determinants of EMT (E‐cadherin, N‐cadherin, Vimentin, Slug), pluripotency and stemness maintenance (SOX2, OCT3/4, NANOG), dedifferentiation (GFAP, GAP43, NF‐L, B3Tubulin), and metastasis (RONB, MMP9, MMP2, CD54, NOTCH1). GAPDH and/or α‐Tubulin served as loading controls. (C) FACS sorting with appropriate inclusion exclusion gating (total, singlets, live, and ALDH positive side population) strategy to classify CSC subsets based on single, double, and triple positive expression of CD133, CD114, and CD117. A total of 10x10^6^ total cells were included across all samples investigated. (D–F) Distribution cris‐cross analysis computing combinations of double positive CSC phenotypes reveals a RD3^−/−^ dependent dynamic shift in stemness composition of each clone investigated and their CSC distribution (vs. RD3^+/−^). All three combinations of double positive comparison unequivocally identified low CSC enrichment in treatment‐naïve (CHLA‐15 and CHLA‐42) cells, while therapy‐defying PD RD3^−/−^ cells (e.g., CHLA‐61, SK‐N‐FI) exhibited unparalleled CSC enrichment. (G) Histogram showing RD3‐loss associated enrichment of general CSC‐like ALDH^+^ side population in NB. (H) Compared to Dx RD3^+/+^, in‐depth sorting showing RD3 expression associated frequency distribution of single (CD133^+^, CD114^+^, CD117^+^); double (CD133^+^CD114^+^, CD114^+^CD117^+^, CD133^+^CD117^+^) and triple (CD133^+^CD114^+^CD117^+^) population CSC enrichment in PD‐RD3^−/−^. **(I)** All PD‐ RD3^−/−^ NB investigated consistently exhibited heightened levels of deadly triple‐positive CSC population compared to the disease at Dx.

With our critical finding that unearthed the *de novo* acquisition of RD3‐loss with therapy pressure that coordinates the evolution of progressive NB [18], herein we first validated whether therapy exposure directly enforces RD3 depletion. We utilized a panel of primary‐ and metastatic‐site derived cells from NB patients during Dx (n = 2) and PD after intensive multimodal clinical therapy (IMCT; n = 12). Total protein input (5µg or 10µg) normalized ELISA identified a definite and significant cell‐line (and patient) independent loss of RD3 in PD when compared with cell lines derived from primary (CHLA‐15) and Bone marrow (BM)‐metastatic (CHLA‐42) disease at Dx (Figure 2A). Imperatively, cells derived from the same patient during Dx (CHLA‐15) and PD (CHLA‐20) affirmed the acquisition of RD3‐diminution with therapy pressure. Defining whether the therapy‐pressure acquired RD3 loss is universal or drug‐specific, three independent Dx RD3^+/+^ systems were exposed to three cycles of vincristine (0.1 µM), doxorubicin (1 µM), cisplatin (10 µM), all used in the induction phase; etoposide (10 µM) used in both the induction and consolidation phase; 13‐cis‐retinoic acid (10 µM) used in the maintenance phase; or docetaxel (0.01 µM) in salvage therapy and examined for RD3 transcription at 24 h and protein levels at 36 and 48 hrs. Compared to the mock‐treated controls, qPCR analysis revealed a drug‐directed complete transcriptional loss of RD3 in all three independent systems analyzed. Relatively, treatment with 13‐cis‐retinoic acid or with docetaxel elicited a lesser degree of RD3 inhibition in these resistant cells. Likewise, immunoblotting displayed a significant loss of RD3 in cells that survive vincristine, doxorubicin, etoposide, and cisplatin treatment. Treatment with 13‐cis‐retinoic acid or with docetaxel inflicted a cell‐specific response with a significant loss in SH‐SY5Y and only a marginal difference in the other two cells investigated. These results corroborate well with the transcriptional loss identified by the qPCR analysis. Pertinently, this drug‐directed acquisition of RD3 loss in surviving cells is typically time‐dependent, with severe loss in 48 h. These observations clearly iterate (i) chemo‐drugs used in the induction and consolidation phase clinical management leads to a significant loss of RD3, while differentiation therapy (13‐cis‐retinoic acid) used during the maintenance phase and docetaxel used for salvage therapy has lesser effect, and (ii) RD3 is transcriptionally lost in response to therapy pressure. To define the function of therapy pressure dictated RD3‐loss in EMT, the coordinated shift in EMT machinery, E‐Cadherin, Slug, Vimentin, and N‐Cadherin in RD3‐deficient PD (PD‐RD3^−/−^) cells was compared with RD3‐expressing Dx (Dx‐RD3^+/+^) cells. Compared to the Dx‐RD3^+/+^ cells, we observed a significant increase in the expression of Vimentin, the intermediate filament protein that provides structural support to mesenchymal cells; N‐Cadherin, the transmembrane protein that contributes to the mesenchymal phenotype; and Slug, a transcriptional repressor for E‐Cadherin in PD‐RD3^−/−^ cells (Figure 2B; Figure S1A). The RD3‐EMT association is evident from the differential expression of these markers between the Dx‐RD3^+/+^ (CHLA‐15) and PD‐RD3^−/−^ (CHLA‐20) clones derived from the same patient (Figure S2A). On the flip side, investigating the relevance of therapy pressure‐depleted RD3 in NB cell maturation and differentiation unveiled a tumor site (primary, metastatic)‐dependent (GAP43, NF‐L) or independent (β3‐Tubulin, GFAP) expression in Dx‐RD3^+/+^ (Figure 2B, Figure S2B). Although the PD‐RD3^−/−^ cells exhibit a site, therapy, and other variables‐dependent heterogeneous expression of these crucial drivers of differentiation, we observed a relative and consistent loss across the cell lines investigated compared to the Dx‐RD3^+/+^ (Figure 2B, Figure S2B). Together, the EMT outcomes and differentiation profile of the RD3‐status defined pre‐ and post‐therapy clones affirmed the loss of lineage commitment and RD3 loss‐associated EMT processes in PD‐RD3^−/−^ (Figure S2B).

Further to determine whether the RD3^−/−^ status triggered lineage plasticity translates to the modulation of CSC dynamics within PD, we studied the discrete landscape of pluripotency maintenance core circuitry determinants, SOX2, NANOG, and OCT3/4. Compared to the Dx‐RD3^+/+^ NB cells, we observed a significant increase in SOX2 and OCT3/4, the binary complex that activates genes responsible for pluripotency and represses differentiation genes in PD‐RD3^−/−^ (Figure 2B; Figure S2C). While NANOG expression showed a site‐dependent fluctuation in Dx‐RD3^+/+^ cells, we observed a consistent increase of NANOG in PD‐RD3^−/−^ cells compared to the BM‐derived Dx‐RD3^+/+^ NB cells (Figure 2B Figure S2C). The increase in these three core regulatory transcription factors in PD‐RD3^−/−^ not only triggers an autoregulatory loop for each other's expression and activity but also ensures gene transcription, which dictates pluripotency and represses differentiation. To determine whether the cellular reprogramming extends to functional traits associated with tumor evolution, we next assessed the RD3‐associated migratory and invasive capabilities of NB. Specifically, we analyzed the expression of RONβ, a transmembrane tyrosine kinase implicated in tumor dissemination; MMP2 and MMP9, extracellular matrix (ECM) metalloproteinases that facilitate ECM remodeling and promote tumor metastasis; CD54, an adhesion molecule involved in transendothelial migration; and NOTCH1, a membrane‐bound receptor protein crucial for enhancing metastatic competency. We found a significant and consistent upregulation of matrix metalloproteinases MMP2 and MMP9 in PD‐RD3^−/−^ clones compared to Dx‐RD3^+/+^ clones (Figure 2B; Figure S2D). In addition, NOTCH1 showed a marked increase in PD‐RD3^−/−^ cells, which aligned with the observed stemness reprogramming and promoted metastatic potential in NB (Figure 2B; Figure S2D). On the other hand, RONβ and CD54 exhibited a more variable, clone‐specific response to RD3‐status (Figure 2B; Figure S2D). While some showed elevated RONβ and CD54 levels, others remained comparable to Dx‐RD3^+/+^ cells, suggesting a inter tumoral context dependent enhanced migratory readiness (Figure 2B; Figure S2D). These findings not only indicate RD3‐loss associated cellular reprogramming towards stem‐like states but also functionally augment metastatic readiness in NB cells through coordinated regulation of migration‐invasion associated effectors.

Real‐time profiling of CSC composition in NB tumors displayed a significant difference in the density and CSC populations between Dx‐RD3^+/+^ and PD‐RD3^−/−^ cells. FACS with FSC and SSC gating with 7‐AAD excluded debris and selectively included live cells (Figure 2C; Figure S3). Stringently limiting the inclusion to ≥80% viable cells to gate Aldefluor^+^ side population which was then sequentially subjected to quadrant gating to distinguish single positivity for putative cell‐surface marker for NB CSCs, CD133 (Prominin‐1, a glycoprotein), CD114 (G‐CSFR, Granulocyte Colony‐Stimulating Factor Receptor), and CD117 (c‐kit, mast/stem‐cell growth factor receptor) (CD133^+^, CD114^+^, or CD117^+^); double^+^ (CD133^+^CD114^+^, CD133^+^CD117^+^, or CD114^+^CD117^+^), and triple^+^ (CD133^+^CD114^+^CD117^+^) CSCs (Figure 2C; Figure S3). This inclusion‐exclusion strategy identified a unique and remarkable enrichment of different CSC subpopulations in PD‐RD3^−/−^ compared to that of Dx‐RD3^+/+^ cells. Imputing the double positive combinations for all the cell lines investigated, PCA analysis displayed the significance of RD3 in determining the NB‐CSC dynamics (Figure 2D–F). Precisely, crisscrossing CD114^+^CD117^+^ with CD133^+^CD117^+^ (Figure 2D) subpopulation identified intense CSC dynamics in RD3^−/−^ CHLA‐61, CHLA‐79, NB‐EBC1, SK‐N‐FI, CHLA‐171 PD (Figure 2E). Also, composing CD133^+^CD117^+^ with CD133^+^CD114^+^ subpopulations identified energetic CSC dynamics in RD3^−/−^ CHLA‐61, CHLA‐79, NB‐EBC1, SK‐N‐FI, CHLA‐171, and CHLA‐20 PD (Figure 2D). Consistently, inputting CD114^+^CD117^+^ and CD133^+^CD114^+^ displayed dynamic CSCs in RD3^−/−^ CHLA‐61, CHLA‐79, NB‐EBC1, SK‐N‐FI, CHLA‐171 PD (Figure 2F). More importantly, these combinations identified that both Dx‐RD3^+/+^ NB cells are CSC naïve, decisively recognizing the relevance of therapy‐pressured RD3‐loss in CSC enrichment in PD. Although the general Aldefluor^+^ side population did not specify pattern (Figure 2G), quantifying the abundance of single^+^ (CD133^+^, CD114^+^, or CD117^+^), double^+^ (CD133^+^CD114^+^, CD133^+^CD117^+^, or CD114^+^CD117^+^), and triple^+^ (CD133^+^CD114^+^CD117^+^) CSCs displayed a consistent and significant increase in CSC composition in PD‐RD3^−/−^ when compared to the Dx‐RD3^+/+^ (Figure 2H,I). Collectively, these outcomes indicate that RD3‐status correlates with lineage plasticity (EMT and differentiation), pluripotency maintenance, and CSC burden and composition in NB.

**FIGURE 3:**
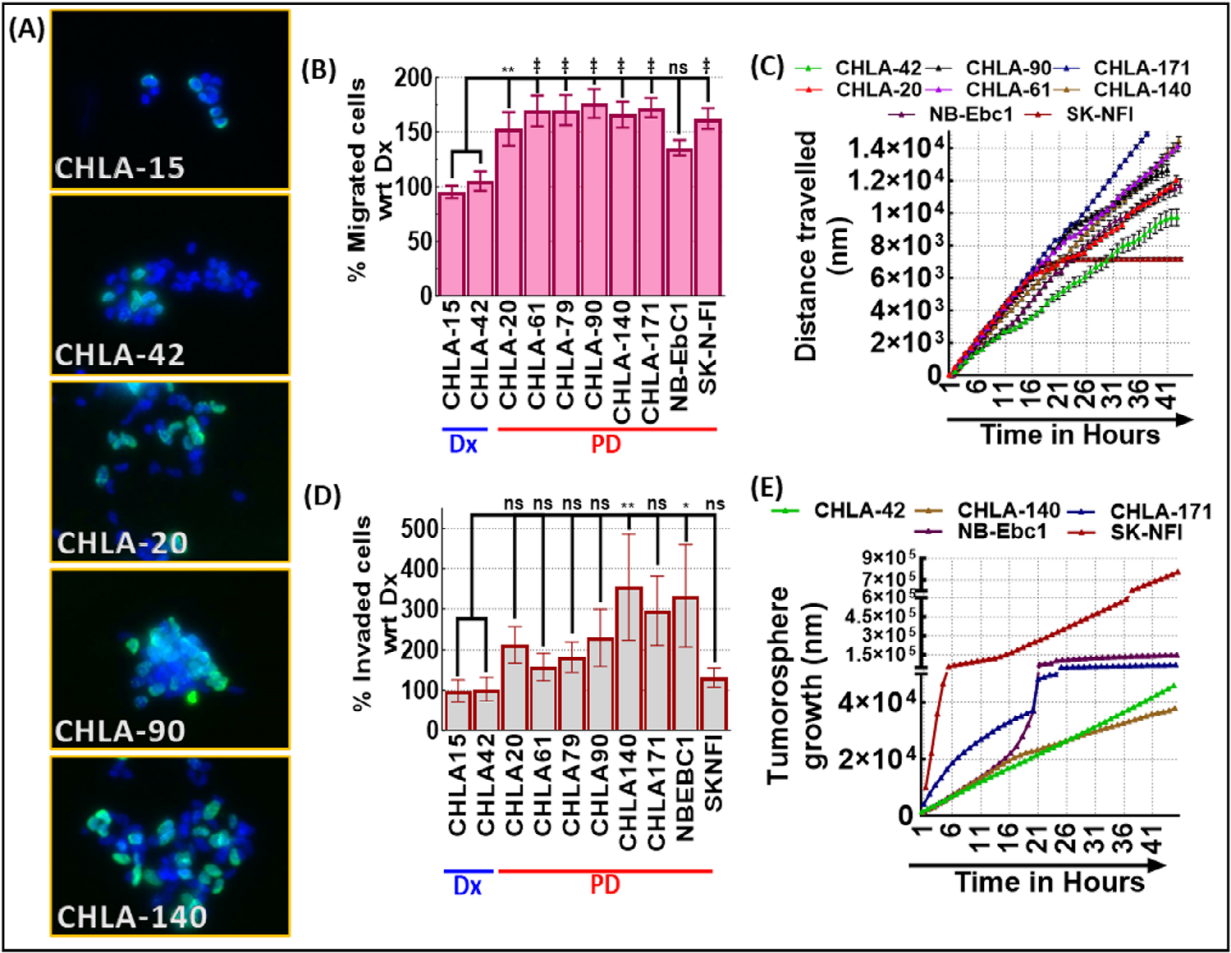
Functional characterization of RD3‐dependent clonal expansion, migration, and invasion in NB (A) Representative images from BrdU incorporation assay performed in Dx‐RD3^+/+^ (CHLA‐15, CHLA‐42) and PD‐RD3^−/−^ (CHLA‐20, CHLA‐90, CHLA‐140) depicting RD3‐loss associated clonal enrichment. (B) Histograms of trans well migration assay performed to evaluate RD3 status‐associated motility in a panel of patient‐derived NB clones, including Dx‐RD3^+/+^ (n = 2) and PD RD3^−/−^ (n = 8) NB *ex‐ vivo* clones. PD‐RD3^−/−^ clones exhibited enhanced migratory potential compared to RD3^+/+^ clones. (C) Quantitative analysis of migratory distance travelled by individual cells measured via live cell time‐lapse imaging and cell tracking algorithm. Consistently, RD3^−/−^ status at PD corresponded to increased migratory distance travelled compared to RD3^+/+^ CHLA‐42 at Dx. (D) Matrigel‐based invasion assay was performed to elucidate RD3‐mediated regulation of invasive potential in a panel of 10 NB clones. Analyzing and quantifying the invaded cells revealed therapy pressure‐driven RD3‐loss enhanced pro‐invasive phenotype in NB. (E) LDTA performed under non‐adherent conditions to elucidate anchorage‐independent growth and stem‐like behavior demonstrated increased sphere‐forming efficiency in RD3^−/−^ clones compared to Dx‐RD3^+/+^ clones. Data are presented as mean ± SEM across biological replicates.

Realization of this RD3‐loss correlation for the lineage transformation, pluripotency maintenance, CSC composition, and burden could be attributable to the evolution of PD. Examining the hallmarks of tumor (migration, invasion, clonal expansion) evolution in the Dx‐RD3^+/+^ and PD‐RD3^−/−^ *ex vivo* systems revealed a consistent association between therapy‐pressure driven RD3‐loss and CSC dynamics during disease progression. While both the Dx‐RD3^+/+^ and PD‐RD3^−/−^ clones displayed a cell‐line‐, site‐, therapy‐dependent fluctuations in the endpoints assessed, PD‐RD3^−/−^ clones predominantly displayed heightened clonal expansion (Figure 3A), T_u_ migration (Figure 3B,C), invasion (Figure 3D) and anchorage independent tumorosphere growth (Figure 3E) when compared with Dx‐RD3^+/+^ cells. A definitive and significant cell‐line independent increase in cellular migration was evident in PD‐RD3^−/−^ cells when compared with Dx‐RD3^+/+^ (Figure 3B,C). Crucially, the pre‐ and post‐therapy RD3 status associated migration studied in the same patient strongly reflects the association of RD3‐loss and enhanced migratory potential. Consistently, under proliferation‐controlled conditions, we observed a robust increase in T_u_ invasive capabilities as early as 24 hrs in PD‐RD3^−/−^ compared to the Dx‐RD3^+/+^ clones (Figure 3D). Collectively PD‐RD3^−/−^ clones display enhanced migratory, invasive, and proliferative capacities. These *ex‐vivo* observations underscore the correlation of therapy pressure‐depleted RD3 status‐associated cellular dynamics and metastatic competence in NB progression.

### 3.2 RD3 Deficient Metastatic Disease In Vivo Simulate Lineage Plasticity

**FIGURE 4:**
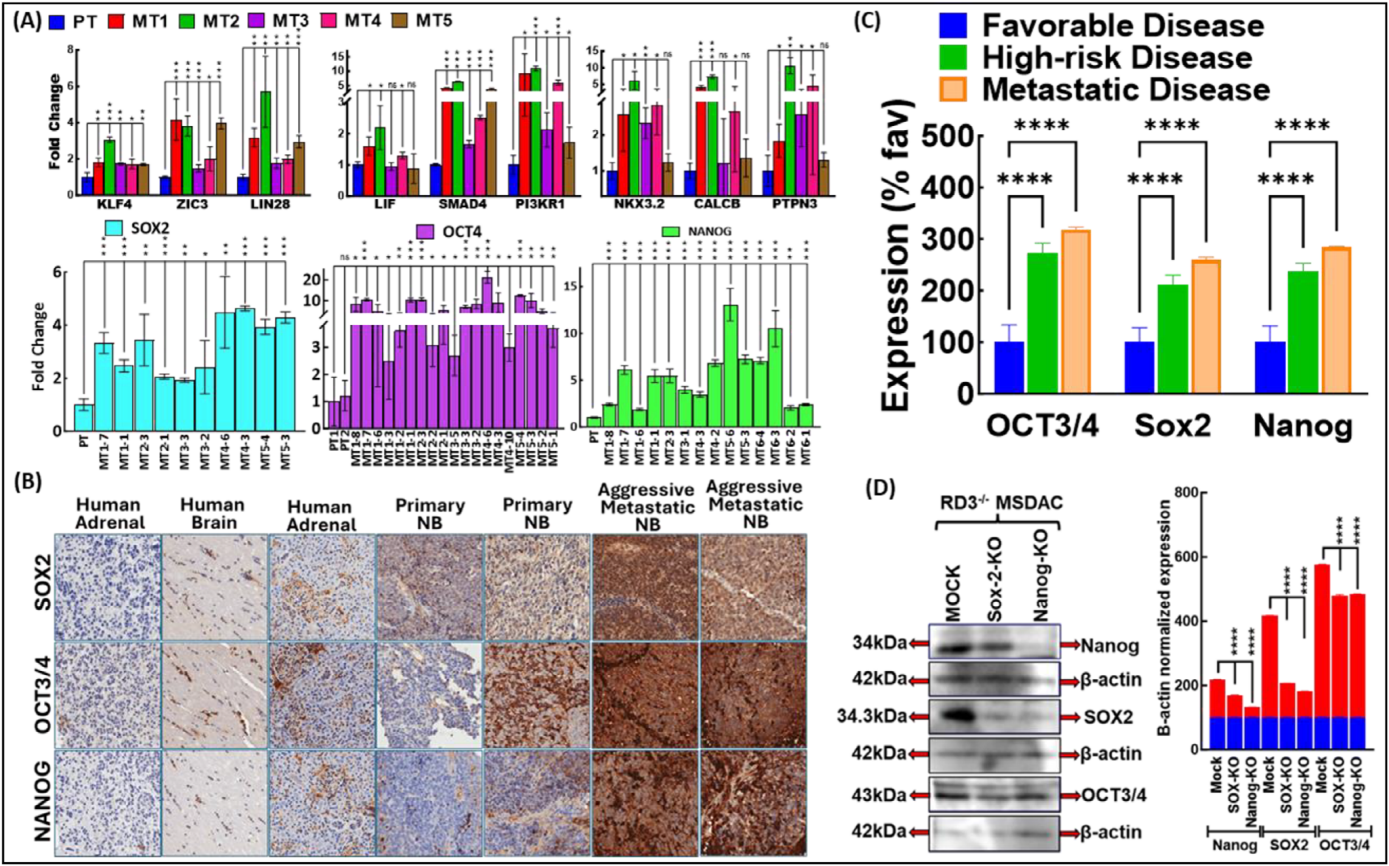
RD3 loss associated with activated pluripotency and plasticity in therapy‐defying NB in‐vivo. (A) qPCR analysis of pluripotency and lineage‐defining markers was profiled to assess RD3‐dependent transcriptional modulation in primary (PT, RD3 expressing) and metastatic (MT, RD3‐deficient) tumors developed in mice utilizing SH‐SY5Y NB cells. Top panel shows RD3‐loss‐associated increase in the transactivation of the crucial plasticity determinants KLF4, ZIC3, LIN28, LIF, SMAD4, PI3KR1, NKX3.2, CALCB, and PTPN3 in RD3‐deficient metastatic tumors (vs. PT). Bottom panel shows transcriptional activation of pluripotency core circuitry TFs SOX2, NANOG, and OCT3/4 in RD3‐deficient MT when compared with PT with inherent RD3 presence. (B) Representative microphotographs of SOX2, NANOG, and OCT3/4 IHC affirming the RD3‐associated regulation of the pluripotency triad at the translational level. Tumors derived from PT (RD3^+/+^) and MT (RD3^−/−^) were assessed for expression of SOX2, NANOG and OCT3/4 proteins. Positivity and cellular localization were validated in normal adrenal and brain tissues. RD3‐deficient MT tumors exhibited elevated levels of all three markers, confirming that RD3‐loss modulates pluripotency translational level. (C) Aperio quantification of IHC for pluripotency‐associated pillars in PT‐RD3^+/+^ and MT‐RD3^−/−^ highlights RD3‐dependent shift in protein abundance and spatial distribution of SOX2, NANOG and OCT3/4. (D) Independent functional ex vivo validation of RD3's role in sustaining pluripotency circuitry. Using metastatic site tumor‐derived MSDAC clones that lack RD3^−/−^, a targeted silencing of SOX2 and NANOG was performed to elucidate the definitive interdependence of the core transcription triad. All three TFs showed high constitutive levels of expression RD3^−/−^ MSDAC. Silencing SOX2 and NANOG in the RD3^−/−^ MSDAC resulted in downregulation of NANOG and SOX2, respectively. In addition, muting either SOX2 or NANOG exerts partial regulation of OCT3/4. This alternate muting experiment in RD3^−/−^ MSDAC validated the existence of a self‐reinforcing autoregulatory loop that maintains pluripotency under RD3‐deficient conditions. PT, primary tumor; MT1‐MT‐5 denote MT tumors from five different animals; multiple tumors per animal are designated as MT1‐1, MT‐7 etc.

To substantiate the definitive correlation of RD3 to the T_u_ identity through altered self‐renewal and pluripotency, we profiled the transcription of crucial neuronal plasticity determinants including Krüppel‐Like Factor 4 (KLF4, neurite formation, cell growth, differentiation, survival); Zinc Finger Transcription Factor of Cerebellum 3 (ZIC3, inhibit neuronal differentiation, maintaining cells in an undifferentiated state); LIN28 (crucial regulator of the transition between pluripotency and committed cell lineages); Leukemia Inhibitory Factor (LIF, promote cancer stemness and phenotypic plasticity, enabling cancer cells to evade a terminally differentiated state and proliferate); SMAD4 (epithelial‐mesenchymal plasticity by integrating TGFβ and BMP signaling); Phosphoinositide‐3‐kinase regulatory subunit 1 (*PI3KR1*, metabolic and cellular plasticity)*, NKX3.2* (maturation inhibitor), Calcitonin related polypeptide beta (*CALCB*, neuronal plasticity); Protein Tyrosine Phosphatase Non‐Receptor Type 3 (*PTPN3*, cell growth, differentiation, and oncogenic transformation) in the panel of primary and metastatic tumors developed in mice utilizing patient derived (during Dx) metastatic (brain) site derived aggressive (SH‐SY5Y) cells [25, 27]. Our earlier studies have sequentially validated the transcriptional and translational loss of RD3 in HR metastatic (vs. primary) tumors [25]. Coherent with the RD3‐loss, we observed a significant transactivation of *KLF4, ZIC3, LIN28, LIF, SMAD4*, and *PI3KR1* in metastatic tumors (Figure 4A). Notably, disease aggression‐associated RD3 loss paralleled transactivation of these determinants, which was not only robust, but also consistent across multiple metastatic sites within an animal, as well across the animals (Figure 4A). The coordinated activation of the canonical transcriptional regulators (*KLF4, ZIC3, LIN28, LIF*) and its’ downstream signaling cascade effectors *SMAD4* and *PI3KR1* suggest disease aggression‐driven RD3‐loss enhanced NB lineage flexibility. Affirming this transcriptional profiling of *SOX2, OCT3/4*, and *NANOG*, the core circuitry drivers of pluripotency maintenance in primary and metastatic tumors mirrored the transcription profile of plasticity markers with significantly high transcription of *SOX2, OCT3/4, and NANOG* consistently across metastatic tumors within and between animals (Figure 4A). Assessing the flow‐through of these transcriptional events, we profiled the translated protein levels of SOX2, NANOG, and OCT3/4 in primary and metastatic tumors (Figure 4B). Normal human adrenal, kidney, and brain tissues are used as controls. IHC revealed a strong SOX2, NANOG, and OCT3/4 nuclear positivity in tumors. Within tumors, we found inter‐ and intra‐tumoral heterogeneity in the expression of these pluripotency maintenance proteins. In stark contrast to the primary favorable disease, we observed a significant (p<0.0001) increase in the translation of SOX2, NANOG, and OCT3/4 in HR‐disease in the primary site and in metastatic tumors (Figure 4C). This compelling evidence supports a strong association between RD3‐loss and the activation of plasticity and stemness determinants at both transcriptional and translational levels. Moreover, the selective induction of SOX2, OCT3/4, and NANOG in aggressive metastatic disease but not in normal and primary tumors underscores its critical role as a gatekeeper of tumor plasticity.

Emphasizing the functional relevance of RD3 loss in promoting phenotypic adaptability, we adopted targeted analysis using RD3^−/−^ metastatic site‐derived aggressive cells (MSDAC) *ex‐vivo*. Three independently derived MSDAC clones were examined for tumorigenic properties, including self‐renewal, proliferation, and pluripotency. Specifically, dissecting out the regulatory interdependency within the circuitry network, we employed a switch‐on/off approach muting *SOX2* or *NANOG* individually, and assessed the expression dynamics of all‐three core components. RD3^−/−^ MSDACs displayed significantly high levels of SOX2, NANOG, and OCT3/4 and served as the positive controls (Figure 4D). Muting SOX2 resulted in a significant reduction of both NANOG and OCT3/4 in RD3^−/‒^MSDACs (Figure 4D). Likewise, silencing NANOG led to substantial downregulation of SOX2 and OCT3/4 in NANOG^−/−^RD3^−/−^ MSDAC system (Figure 4D). Crucially, these findings demonstrated a strict interdependency between NANOG and SOX2 (Figure 4D). Interestingly, fractional regulation of OCT3/4 was observed with muting SOX2 or NANOG, indicating partial interdependency on the binary proteins. Collectively, these data reveal a tightly coupled and mutual regulatory axis between SOX2 and NANOG, with OCT3/4 playing a partial independent node in RD3‐deficient NB. Further, these results indicate that RD3 deficiency facilitates the establishment and maintenance of a stable stem‐like phenotype with the core molecules SOX2, OCT3/4, and NANOG functioning as a self‐reinforcing regulatory loop.

### 3.3 RD3 Engineer NB‐CSC Identity

**FIGURE 5:**
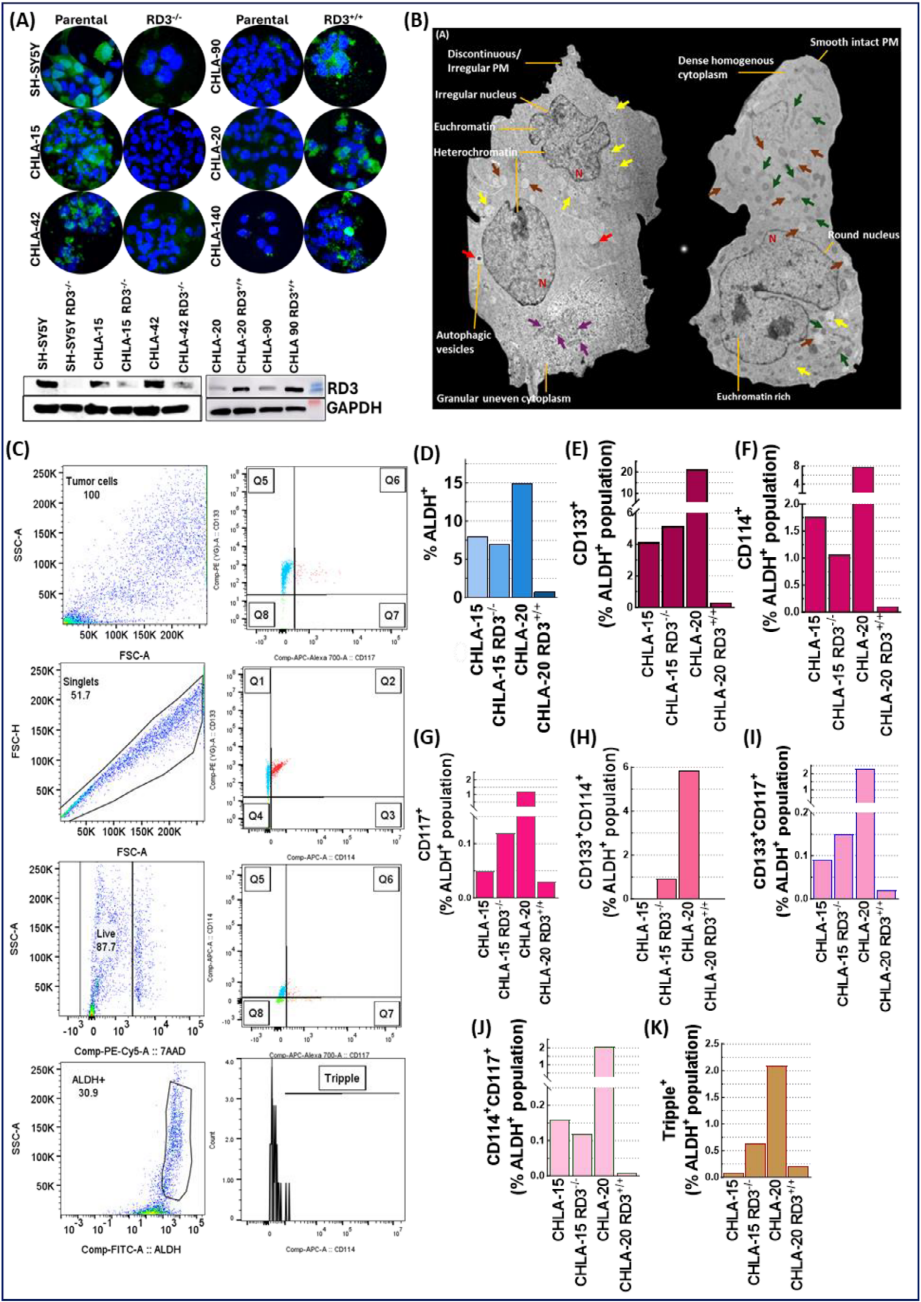
RD3 engineers NB cell identity and CSC composition. (A) Stable silencing of RD3 in RD3 expressing CHLA‐15, CHLA‐42, SH‐SY5Y (designated as CHLA‐15 RD3^−/−^, CHLA‐42 RD3^−/−^, SH‐SY5Y RD3^−/−^) and stable restoration of RD3 in RD3‐deficient CHLA‐20, CHLA‐90, CHLA‐140 (designated as CHLA‐20 RD3^+/+^, CHLA‐90 RD3^+/+^, CHLA‐140 RD3^+/+^) *ex‐vivo* systems were validated with IF and WB. Representative IF images (top panel) shows RD3 localization and intensity, and immunoblot (bottom panel) confirms RD3 expression status across conditions following stable KO and re‐expression in NB patient derived cells, validating the engineered models generated (B) Ultrastructural and morphological alterations (plasma membrane integrity, nuclear morphology, chromatin organization, organelle distribution, cytoplasm: nuclear ratio) following RD3 KO in RD3^+/+^ CHLA‐15 visualized via TEM. RD3^−/−^ clones displayed smooth intact membranes, homogenous cytoplasm, euchromatin‐rich nuclei, and abundant mitochondria associated with lipids, consistent with lipid‐fueled metabolic phenotype. RD3^+/+^ clearly depicted features indicative of relative cellular stress, including irregular membrane, condensed chromatin, granular uneven cytoplasm, suggesting compromised homoeostasis and integrity. Colored arrows denote key features including mitochondrial morphology (dark green), vesicular trafficking (yellow), lipid moieties (brown), lipophagy (light blue), lysosomal contents (purple) and autophagosome (red). Magnification 2000x. (C) FACS sorting with appropriate inclusion exclusion gating (total, singlets, live, and ALDH^+^ side population) strategy to classify CSC subsets based on single, double and triple positive expression of CD133, CD114, and CD117. A total of 1x10^6^ total cells were included across all samples investigated. (D) ALDH^+^ cells were computed to identify stem‐like side populations across Dx‐RD3^+/+^, PD‐RD3^−/−^, RD3^−/−,^ and RD3^+/+^ ex‐vivo systems. DEAB treated controls were used to define baseline fluorescence. RD3^+/+^ cells exhibited a significant decrease of ALDH^+^, indicative of RD3‐regulated stemness traits in NB, while RD3‐loss promotes enrichment of functionally defined CSC compartments. (E–G) An abundance of single (CD133, CD114, CD117) positive CSC populations across RD3 reverse‐engineered systems. Appropriately matched parental strains were used as controls. Compared to the RD3^−/−^ CHLA‐20, its RD3^+/+^ engineered clone showed a remarkable reduction in all three single CD133^+^, CD114^+^, CD117^+^ populations, indicating constrained stemness and lineage fidelity. Conversely, forced silencing of RD3 in CHLA15 showed defined enrichment of these single positive CSC subsets, corroborating the role RD3 as a suppressor of CSC expansion and phenotypic plasticity. (H–J) FACS performed to assess the RD3‐modualted expansion of dual positive CD133^+^CD114^+^, CD133^+^CD117^+^, CD114^+^CD117^+^ CSC populations. Forced silencing RD3^−/−^ cells promoted the expansion and accumulation of dual positive CSC subsets in NB (vs. RD3^+/+^), mirroring the trends observed in single positive subsets. (K) Triple marker analysis revealed a distinct expansion of CD133^+^CD114^+^CD117^+^ aggressive CSC subset in RD3^−/−^ cells, compared to RD3^+/+^ counterpart; and on the flipside forced re‐expression of RD3 in PD‐RD3^−/−^ resulted in complete negation of this deadly subset expansion. The marked increase in triple‐positive subsets underscores RD3's critical role as a suppressor of CSC expansion and stemness maintenance.

To explicitly define RD3 function in lineage commitment (EMT, dedifferentiation), pluripotency maintenance, CSC composition, we adopted a RD3 reverse engineering strategy in six unique bed‐to‐bench systems. Armed with our clinical (RD3 status, site, risk, primary/metastatic, diagnosis/treatment etc.,) and functional (migration and invasive capabilities, pluripotency, and stemness maintenance, dedifferentiation, tumorosphere formation, clonal expansion, and tumor immune evasion) characterization of diverse ex‐vivo systems [18, 22, 25, 30, 22], we appropriated an inclusion/exclusion criteria to select three Dx‐RD3^+/+^ (CHLA‐15, CHLA‐42, SH‐SY5Y) and another three PD‐RD3^−/−^ (CHLA‐20, CHLA‐90, CHLA‐140) systems. For stable reinstatement of RD3 in PD‐RD3^−/−^ systems, the RD3 transcript variant 1 cloned into pCMV6‐AC was transfected and selected using G418. The stably reinstated clones were designated as CHLA‐20 RD3^+/+^, CHLA‐90 RD3^+/+^, and CHLA‐140 RD3^+/+^. For stably silencing RD3 in Dx‐RD3^+/+^ (CHLA‐15, CHLA‐42, SH‐SY5Y) clones, we utilized RD3 shRNA construct GCTGGAAGATGTCTGCGTTAA in pLKO.1 – puro vector (Sigma, St. Louis, MO, USA) and the knocked‐out clones were selected using puromycin. The stable RD3‐KO clones are designated as CHLA‐15 RD3^−/−^, CHLA‐42 RD3^−/−^, and SH‐SY5Y RD3^−/−^. The stable restoration and/or silencing of RD3 was investigated with ICF and validated with immunoblotting (Figure 5A). RD3 IF staining with Alexafluor488 exhibited specific localization in nucleus, perinuclear, and cytoplasmic regions. Compared to the Dx‐RD3^+/+^ CHLA‐15, CHLA‐42, and SH‐SY5Y, IF and immunoblotting validated the complete knockout of RD3 in CHLA‐15 RD3^−/−^, CHLA‐42 RD3^−/−^, and SH‐SY5Y RD3^−/−^ (Figure 5A). Like‐wise, compared to the PD‐RD3^−/−^ CHLA‐20, CHLA‐90, CHLA‐140 cells, we observed stable restoration RD3 in CHLA‐20 RD3^+/+^, CHLA‐90 RD3^+/+^, and CHLA‐140 RD3^+/+^ cells (Figure 5A). With this stable silencing and expression engineered system, we have a validated set of 12 ex‐vivo unique models for unequivocally define RD3 function in defining NB‐CSC identity.

#### 3.3.1 RD3 Curbs the Shift in NB Cell Organelle Composition, Ultra‐Structure, and Morphometrics

Changes in NB cell morphometrics including shape, size, colony formation etc., mirror the complex interplay of internal cellular (EMT, stemness, differentiation etc.,) processes those maps the continuum of cellular plasticity. Phase contrast imaging of three independent models of PD‐RD3^−/−^ including CHLA‐20, CHLA‐90, CHLA‐140 cells displayed cellular clusters with formation of organized tumorospheres (Figure S4A Top). Conversely, forced re‐expression of RD3 in these RD3^−/−^ PD cells inhibited tumorosphere formation and promoted neurite formation and monolayer spreading (Figure S4A). Likewise, the RD3 expressing Dx‐RD3^+/+^ CHLA‐15, CHLA‐42, and SH‐SY5Y systems exhibited a less motile, polarized morphology with neurite extensions showing typical hallmarks of differentiation (Figure S4A, lower panel). Parallel profiling of T_u_ architecture with muted RD3 (CHLA‐15 RD3^−/−^, CHLA‐42 RD3^−/−^, SH‐SY5Y RD3^−/−^) exhibited remarkable disruption in apico‐basal polarity that was evidenced by formation of tumorosphere indicating a pronounced anchorageindependent growth, EMT‐driven detachment and enhanced metastatic potential (Figure S4A, lower panel). Together, this complete systems approach with engineered RD3 levels displayed a cell‐line, disease and site independent function of RD3 in directed cytoskeletal reorganization modifying cellular size, shape, monolayer, or tumorosphere formation, etc., all conducive to RD3‐governed NB differentiation.

Substantiating further, in‐depth analysis with TEM revealed striking ultrastructural variations between RD3‐null and RD3‐expressing NB cells reflecting the distinct patterns of subcellular organization, organelle abundance, and distribution (Figure 5B; Figure S4B). RD3^−/−^ cells exhibited homogenous cytoplasmic volume with densely populated cellular organelles consistent with a hypermetabolic phenotype (Figure 5B; Figure S4B). Precisely, when compared with RD3^+/+^ cells RD3‐deficient cells showed marked increase both in the number and size of mitochondria (Figure 5B; Figure S4B). In particular, these mitochondria displayed spatial associations with lipid moieties suggestive of lipid‐fueled bioenergetics; however, functional validation is required to confirm substrate utilization and bioenergetic shift. Lipid droplets were abundant and often engaged lipophagy in RD3^−/−^ cells as evidenced by the presence of large autophagosomes and phagolysosomes containing unmetabolized lipids, indicating impaired lipid processing and/or enhanced lipid turnover (Figure 5B; Figure S4B). The cytoplasm displayed a smoother texture, with less prominent endoplasmic reticulum, further affirming overwhelmed lipid metabolism. Notably, the plasma membrane of RD3^−/−^ cells was smooth and continuous, indicating intact cell boundaries and pronounced structural stability. Likewise, the nuclear membrane was intact and observed to be enriched with euchromatin, consistent with normal transcriptional activity and functioning. Conversely, RD3^+/+^ cells showed hallmark of quiescent phenotype and homeostatically balanced state with an enlarged cytoplasmic‐to‐nuclear ratio (Figure 5B; Figure S4B). Mitochondria and lipid droplets were sparse, indicating reduced metabolic flux and lipid utilization. Interestingly, these cells exhibited both early and late endosomes, indicative of active intracellular trafficking and a basal level of maintenance. Furthermore, RD3^+/+^ cells showed irregular plasma membrane with regions of blebbing and discontinuity, chromatin condensation in the nucleus relatively, indicating cellular stress. In addition, RD3^+/+^ cells exhibited a notably enriched rough endoplasmic reticular network compared to their RD3^−/−^ counterparts that relied on lipid metabolism. Collectively, these morphometric and ultrastructural findings reveal that RD3‐loss not only configures organelle distribution and energy utilization but also regulates lineage and metabolic plasticity, reinforcing its role as a molecular gatekeeper of NB cellular adaptability.

#### 3.3.2 RD3 Deficiency Directed Brake Failure Fuels CSC‐Rich NB

Utilizing the rigorous immunophenotyping strategy, we dissected the NB CSC dynamics through sequential sorting of Aldefluor‐based side population, lining up with single, double, and triple combinations using canonical CSC marker CD133 coupled with and NB‐CSC specific CD114 and CD117 (Figure 5C,D). For pinning the function of therapy‐pressure dictated RD3‐loss in increasing NB‐CSC burden, we compared Dx‐RD3^+/+^ CHLA‐15 and PD‐RD3^−/−^CHLA‐20 systems, both derived from the same patient pre‐ and post‐therapy with known RD3 status, and their RD3 reverse engineered (stable silencing and reinstating, respectively) counterparts. Compared to the Dx‐RD3^+/+^ cells, ALDH^+^CD133^+^, ALDH^+^CD114^+^, ALDH^+^CD117^+^ CSCs were profoundly enriched (2–5 fold increase) in PD‐RD3^−/−^ cells. (Figure 5E–G). Muting RD3 in Dx‐RD3^+/+^ cells instigated a RD3‐loss dependent expansion of ALDH^+^CD133^+^ and ALDH^+^CD117^+^ subpopulations (Figure 5E,G). Conversely, re‐expression of RD3 in PD‐RD3^−/−^CHLA‐20 completely abrogated the expansion of ALDH^+^CD133^+^, ALDH^+^CD114^+^, ALDH^+^CD117^+^ CSCs, pinning the function of RD3 in regulating the selection and enrichment of these CSC subpopulations (Figure 5E–G). Further, FACS revealed that the aggressive dual positive stem cell subpopulations ALDH^+^CD133^+^CD114^+^, ALDH^+^CD113^+^CD117^+^, ALDH^+^CD114^+^CD117^+^together comprise <2% of the total population in Dx‐RD3^+/+^ CHLA‐15 (Figure 5H‐J). Compared to these Dx‐RD3^+/+^ cells, PD‐RD3^−/−^ CHLA‐20 cells exhibited a significantly high composition of ALDH^+^CD133^+^CD114^+^, ALDH^+^CD113^+^CD117^+^, ALDH^+^CD114^+^CD117^+^ double‐positive populations. Contrastively, stable RD3 restoration led to the complete decline of all three dual positive CSC population enrichment (Figure 5H–J). Significantly, the rare and resistant ALDH^+^CD133^+^CD114^+^CD117^+^ triple‐positive CSCs emerged as the most tightly RD3‐regulated subpopulation (Figure 5K). Both intrinsic RD3‐loss (PD‐RD3^−/−^) due to therapy pressure and induced silencing (CHLA‐15 RD3^−/−^) enriched the triple positive CSCs in NB compared to RD3‐proficient controls Dx‐RD3^+/+^ and RD3 reinstated CHLA‐20 RD3^+/+^ (Figure 5K). Taken together, RD3‐deficiency profoundly reprograms the CSC composition, essentially enriching both general and lineage‐specific CSC subpopulations in NB. With this sequential immunophenotyping, we demonstrate that RD3‐loss fuels expansion of aggressive CSC niches and indicate that RD3 is a central regulator of malignant reprogramming by promoting the expansion of rare and highly malignant CSC populations, redefining the stemness landscape in NB.

#### 3.3.3 RD3 Deters EMT

**FIGURE 6:**
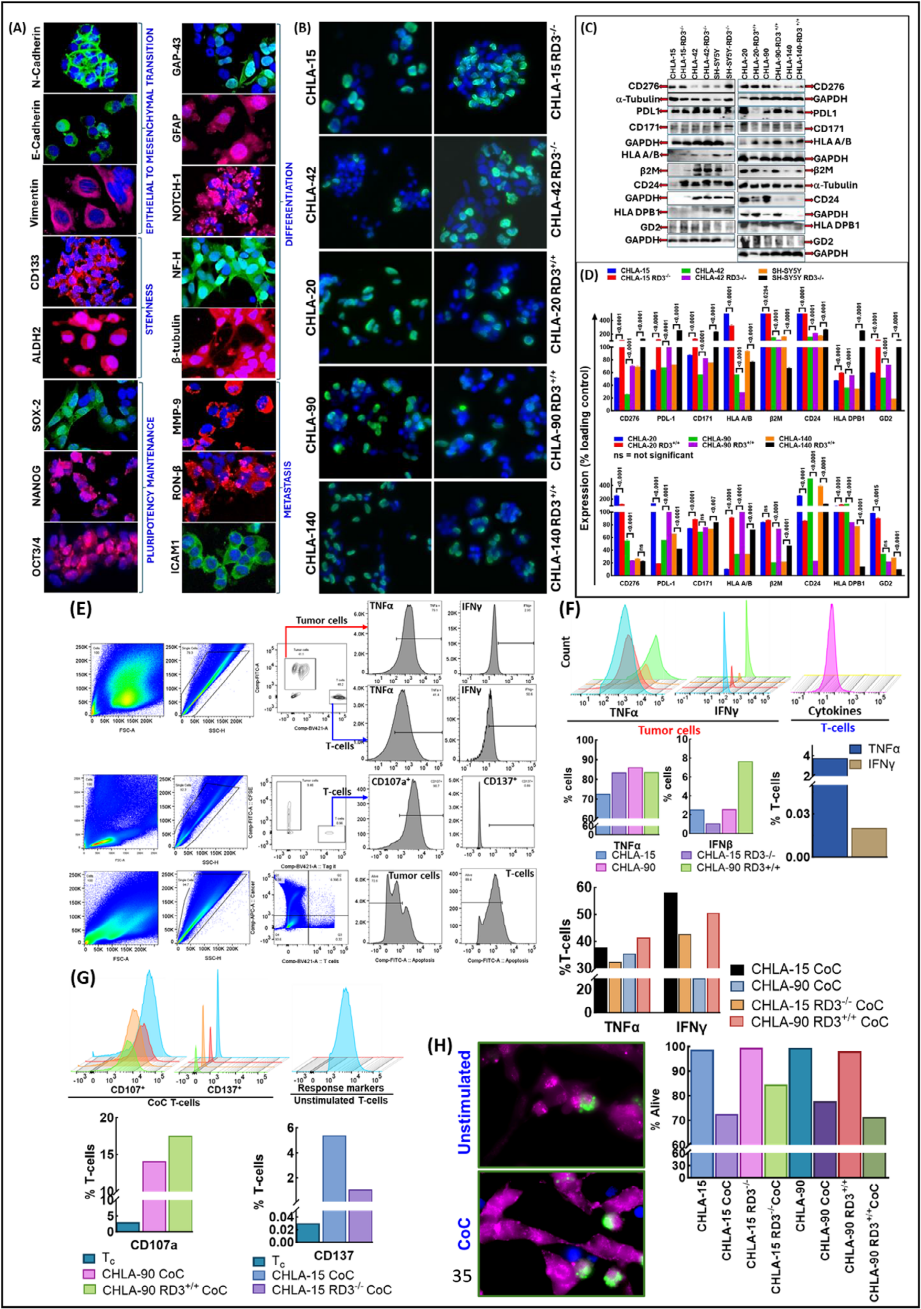
RD3 impedes cellular plasticity and governs immune editing in NB (A) Representative images from multi‐IF analysis of plasticity‐associated core pluripotency components (SOX2, NANOG, OCT3/4), stemness effectors (ALDH2, CD133), EMT drivers (E‐CAD, N‐CAD, Vimentin), NB maturation determinants (GFAP, GAP43, β‐3‐Tubulin, NF‐H). and metastasis facilitators (MMP9, RON‐β, ICAM1) across a panel of RD3 reverse‐engineered clones (n = 6) and compared to their parental counterparts (n = 6). IF reveals RD3^−/−^ directed multi‐layered reprogramming towards a stem‐like, mesenchymal phenotype with lineage plasticity and heightened pluripotency in NB. Comparisons of the relative expression for each of these fifteen crucial determinants in all twelve systems were pictured and compared in Figure S4A–E. (B) BrdU incorporation assay was performed across Dx‐RD3^+/+^ CHLA‐15, CHLA‐42; PD‐RD3^−/−^ CHLA‐20, CHLA‐90, CHLA‐140; RD3‐muted CHLA‐15 RD3^−/−^, CHLA‐42 RD3^−/−^ and RD3‐reinstated CHLA‐20 RD3^+/+^, CHLA‐90 RD3^+/+^, CHLA‐140 RD3^+/+^ to elucidate RD3 regulated clonal expansion. RD3^−/−^ clones showed nuclear BrdU incorporations in higher number of cells compared to Dx‐RD3^+/+^ and RD3‐reinstated clones, confirming RD3's potential in restraining hyperplastic state and clonal propagation. (C) RD3‐loss driven plasticity‐regulated immune response in NB ‐immunophenotypic profiling of immune visibility (HLA A/B, HLA DPB1, β2M), checkpoint (PDL1, CD276), surveillance (CD171, GD2) and immune evasive (CD24) determinants profiled in RD3‐engineered clones and its parental counterparts. Representative immunoblots and (D) histograms constructed from densitometric quantification of immunoblots showing expression profiles in PD‐RD3^−/−^ and RD3‐muted clones depicting downregulation of antigen presentation machinery, upregulation of immune checkpoint and promoted tumor disguise via CD24 compared to RD3‐expressing counterparts, highlighting RD3‐loss driven modulation of tumor‐immune interactions through plasticity‐driven remodeling (E) Representative FACS gating strategy utilized for functional immune assays, cytokine secretion (TNFα and IFNγ; top panel), tumor reactivity assay (CD107a and CD137; middle) and tumor killing assay (Caspase‐3 activity; bottom panel) (F) TNFα and IFNγ secretion in T_C_ CoC with RD3‐expressing and reinstated tumors induced robust cytokine secretion; T_C_ CoC with RD3‐deficient and kncokout clones elicited minimal response (G) Degranulation (CD107a) and sustained activation (CD137) of T_C_ CoC with RD3‐proficient tumors whereas RD3‐silencing reduced T_C_ effector function. (H) Representative images of T_C_‐mediated apoptosis (left panel). All CoC systems displayed increased caspase activity, confirming T_C_‐mediated cytotoxicity. RD3‐expressing and reinstated tumors showed greater apoptosis of T_u_ compared to RD3‐null clones, indicating an immune evasive phenotype (right panel).

To elucidate the functional flow‐through in RD3‐regulated plasticity, we assessed the RD3‐directed EMT core machinery. For this, first, we assessed the regulatory function of RD3 on the core EMT transcriptional factors (TF), including TWIST, which promotes mesenchymal transition and metastatic competence; SNAIL, a master repressor of E‐CAD, and SLUG, synergizes with SNAIL to reinforce epithelial suppression. For this, we used one RD3‐deficient PD system (CHLA‐90) and one RD3‐expressing Dx system (CHLA‐42) with their matching RD3 stably expressed or stably silenced counterparts. Compared to the RD3 expressing Dx clones, β‐actin normalized QPCR analysis revealed significant transcriptional activation of *TWIST, SNAIL*, and *SLUG* in their RD3‐stablly silenced counterparts (Figure S5A, *Top panel*). Conversely, compared to the RD3‐deficient PD clones, we observed a complete suppression in *TWIST, SNAIL*, and *SLUG* mRNA levels when RD3 is stably re‐expressed (Figure S5A, *Top panel*). Collectively these outcomes undeniably portray that RD3 regulates the transcription of these crucial EMT transcription factors. Second, we assessed the RD3‐directed modifications in EMT core factors (Vimentin, N‐CAD, E‐CAD) in Dx‐RD3^+/+^, PD‐RD3^−/−^, RD3‐muted Dx, and RD3‐restored PD NB. Compared to the RD3^+/+^ Dx cells (CHLA‐15, CHLA‐42, SH‐SY5Y), muting RD3 (CHLA‐15 RD3^−/−^, CHLA‐42 RD3^−/−^, SH‐SY5Y RD3^−/−^) heightened the expression of cytoskeleton remodeling protein VIMENTIN, marking early transition to EMT (Figure 6A; Figure S5A, *Bottom panel*). Congruent to the onset of early‐stage transition to EMT, silencing RD3 results in the reduction in E‐CAD, regulating adherens and tight junctions, altering cell‐cell and cell‐matrix contact (Figure 6A; Figure S5A, *Bottom panel*). Consistently, pronounced clustering of cells was evident with notable globular morphology. Furthermore, knocking out RD3 affected a cell‐line independent “cadherin switching” (increase in N‐CAD paralleling E‐CAD decrease), a hallmark of mesenchymal transition (Figure 6A; Figure S5A, *Bottom panel*). RD3‐muting associated increase in N‐CAD expression was more pronounced in the BM‐ derived cells, reinforcing the conducive metastasis following EMT (Figure 6A; Figure S5A, *Bottom panel*). On the flip side, RD3‐restoration in three independent *ex vivo* systems promoted MET phenotypic outcomes by decreasing the expression of VIMENTIN and N‐CAD while simultaneously restoring E‐CAD expression, promoting epithelial identity (Figure 6A; Figure S4A). These outcomes convincingly demonstrate that silencing RD3 effect MET→EMT and RD3 restoration effect EMT→MET, facilitating cellular plasticity. Collectively, these outcomes position RD3 as a critical checkpoint molecule of mesenchymal dynamics, curbing cellular plasticity and metastatic competence in NB.

#### 3.3.4 RD3 Curtail Stemness and Endorse Lineage Commitment

CD133 and ALDH2 are key determinants of stemness phenotype, however the regulation of its expression in NB remains unexplored. Our study using a panel of patient‐derived cell lines showed a marked up CSC niche with RD3 loss. To affirm the stemness regulatory function of RD3, we screened the fluctuations inflicted in the stemness‐associated CD133 and ALDH2 with RD3 manipulation clones, when compared to their parental controls. IF showed that the innate expression of CD133 and ALDH2 were unequivocally elevated in RD3 deficient systems (PD‐RD3^−/−^‐CHLA‐20, CHLA‐90, CHLA‐140; RD3 KO‐CHLA‐15 RD3^−/−^, CHLA‐42 RD3^−/−^, SH‐SY5Y RD3^−/−^) compared to Dx‐RD3^+/+^ or RD3 restored clones (Figure 6A; Figure S5B). These outcomes undeniably illustrate that RD3 regulates CD133 and ALDH2 expression and negatively influences stem‐like traits.

#### 3.3.5 RD3 Promotes Differentiation

To further substantiate the function of RD3 in directing differentiation, we performed multi‐IF for crucial neuronal differentiation drivers, GFAP, GAP43, β‐3 Tubulin, and NFH alongside NOTCH1, a known differentiation repressor. Corroborating our EMT and stemness inferences, we found robust expressions of GFAP, GAP43, β‐3‐Tubulin, and NF‐H accompanied by pronounced neurite extensions and branching in RD3 expressing CHLA‐15, CHLA‐42, SY‐SY5Y, CHLA‐20 RD3^+/+^, CHLA‐90 RD3^+/+^, and CHLA‐140 RD3^+/+^ cells (Figure 6A; Figure S5C). Conversely, the loss and/or complete silencing of RD3 inflicted a significant decrease in the localization of GFAP, GAP43, β‐3 Tubulin, and NFH, and reflected with clustered morphology and reduced neurite maturation (Figure 6A; Figure S5C). Importantly, RD3^+/+^ cells that expressed higher levels of differentiation markers simultaneously exhibited reduced NOTCH1 expression, reinforcing the inverse relationship of RD3 to stemness maintenance (Figure 6A; Figure S5C). This dual regulatory role positions RD3 as a key molecular determinant of NB cell fate, with potential implications in lineage plasticity.

#### 3.3.6 RD3 Disconcert Pluripotency Circuitry Network and Clonal Expansion

NANOG, SOX2, and OCT3/4 are three crucial transcription factors that play a crucial role in pluripotency maintenance, working together in a complex regulatory network and ensuring the self‐renewal potential of T_u_. With our *ex‐vivo* and in‐vivo studies showing the significant association of RD3 loss to the activation of these TFs (Figure 4), and our demonstration of tight‐interdependency for autoregulation of SOX2‐OCT3/4‐NANOG in RD3 null cells (Figure 6A; Figure S5D), here we elucidate the direct function of RD3 in regulating this key signaling of self‐renewal. Muting RD3 resulted in consistent increase in NANOG‐SOX2‐OCT3/4 triad across RD3‐deficient models, including CHLA‐20, CHLA‐90, CHLA‐140, CHLA‐15 RD3^−/−^, CHLA‐42 RD3^−/−^ and SH‐SY5Y RD3^−/−^ (Figure 6A; Figure S5D). Particularly, our KO clones, as well as parental PD‐RD3^−/−^ showed remarkable elevation in the nuclear localization of these transcription factors, indicating functional activation (Figure 6A and S5D). In contrast, RD3^+/+^ Dx cells (CHLA‐15, CHLA‐42 and SH‐SY5Y) and RD3‐reinstated clones (CHLA‐20 RD3^+/+^, CHLA‐90 RD3^+/+^ and CHLA‐140 RD3^+/+^) showed reduced expression of these factors (Figure 6A and S5D). The reduction of OCT3/4, SOX2, and NANOG proteins in Dx‐RD3^+/+^ and RD3‐restored clones suggests that the reprogramming of these factors is post‐transcriptionally controlled by RD3 in NB. More importantly, dissecting out the mechanism how RD3 regulates pluripotency maintenance, first, we investigated whether RD3 binds to these three core circuitry proteins. RD3 pull‐down and co‐immunoprecipitation (Co‐IP) of SOX2, NANOG, and OCT3/4 in two independent RD3‐deficient PD systems matched with their stable RD3 restored systems clearly indicated a RD3 dose (availability)‐dependent binding of SOX2, Nanog, and OCT3/4 (Figure S5E, *bottom panel*). Crucially, chromatin‐immunoprecipitation (ChIP‐qPCR) analysis in four independent systems (two RD3‐deficient PD and two RD3‐harboring DX systems) with matching RD3 stable expression or stable knockout counterparts unveiled an RD3 binding‐dependent regulation of *SOX2*, *NANOG*, and *OCT3/4* promoter activity (Figure S5E, *bottom panel*). Relatively, there is a strong and significant association of RD3 repressor activity for SOX2, while a cell‐line dependent intensity variations are evident for NANOG and OCT3/4 (Figure S5E, *bottom panel*). Collectively, these findings reveal a thus far unrealized function of RD3 as a critical upstream regulator of pluripotency and lineage fidelity in NB, acting primarily by sequestering SOX2, NANOG, and OCT3/4 in protein complexes and restricting their chromatin engagement.

To assess the function of RD3‐derranged NANOG, SOX2, OCT3/4 autoregulation in clonal expansion, we performed the BrdU incorporation assay in our 12 cellular systems: Dx‐RD3^+/+^ (CHLA‐15, CHLA‐42, SH‐SY5Y); PD‐RD3^−/−^ (CHLA‐20, CHLA‐90, CHLA‐140); RD3‐muted (CHLA‐15 RD3^−/−^, CHLA‐42 RD3^−/−^, SH‐SY5Y RD3^−/−^); and RD3‐restored (CHLA‐20 RD3^+/+^, CHLA‐90 RD3^+/+^; CHLA‐140 RD3^+/+^) models (Figure 6B). BrdU incorporation serves as a measure of active dividing and cell cycle progression through S‐phase. RD3^−/−^ systems demonstrated a heightened clonal expansion, reflecting accelerated entry into S‐phase with robust increase in DNA synthesis (Figure 6B). These heightened levels of DNA synthesis were consistent in both our RD3^−/−^ model system. Conversely, reinstating RD3 led to substantial reduction of DNA synthesis and constrain cell cycle progression contributing to a more quiescent state (Figure 6B). Further the switch to hypoproliferative phenotype strongly supports RD3's role as a negative regulator of cell proliferation and its absence disrupts normal cell cycle checkpoint mechanisms, allowing for uncontrolled progression through G1/S transition. Moreover, in non‐transformed systems, including Dx‐RD3^+/+^ and PD‐RD3^−/−^ showed a RD3 availability‐dependent cell‐independent clonal expansion (Figure 6B). Together, these results critically underscore RD3's role in mitotic regulation and suggest its ablation leads to uncontrolled proliferation and tumor progression.

#### 3.3.7 RD3 Deprived Lineage Plasticity Preclude Tumor Dissemination

Building on RD3 directed fluctuations in EMT, stemness, dedifferentiation, and pluripotency maintenance, we investigated whether this phenotypic shift translates into NB metastatic spread. Dissecting out the designated hallmarks of metastasis, T_u_ migration and invasion we evaluated the induced changes of metastatic facilitators including Intercellular Adhesion Molecule 1 (ICAM1), a transendothelial migration inducer; matrix metalloproteinase (MMP9), an extracellular matrix (ECM) remodeling protein that degrades collagen type IV and; RON‐β, a variant of receptor tyrosine kinase facilitating spread in RD3‐silenced and restored systems alongside Dx‐RD3^+/+^ and PD‐RD3^−/−^. Forced restoration of RD3 suppressed the translation of metastatic coordinators MMP9, ICAM1, and RON‐β, and corroborated our findings on EMT, dedifferentiation, stemness maintenance, and self‐renewal. In contrast, depletion of RD3 from T_u_ displayed a consistent elevation in MMP9, ICAM1, and RON‐β localization promoting tumor dissemination (Figure 6A; Figure S5F). Together, RD3 depletion from the system activates a pro‐metastatic program that involves ECM degradation, transendothelial migration, and invasive signaling resulting in intravasation. Our findings decisively pin RD3's pivotal role in regulating metastatic fate in NB cells.

Comprehensively, our twelve‐system approach with three independent models dedicated to each: intrinsic RD3 available; RD3 muting; intrinsic RD3 deficient; and RD3 restoration establishes RD3 as a master regulator of T_u_ plasticity in NB by governing the intricate interplay between stemness, differentiation EMT‐MET balance, and metastatic competence. Communally, our findings showed that RD3 depletion reactivates the embryonal pluripotency network through SOX2‐OCT3/4‐NANOG axis, elevating the stemness traits via CD133, ALDH2 expression, resulting in a more dedifferentiated CSC phenotype. Concurrently, its silencing disrupts the fine epithelial architecture in T_u_ by altering the EMT ‐MET dynamics (↓E‐CAD, ↑N‐CAD), facilitating T_u_ motility. This phenotypic switching could lead to the induction of hematogenous metastasis in NB via ECM degradation, transendothelial migration, and intravasation, evidenced by upregulation of ICAM1, MMP9, and RON‐β. Contrarily, restoration of RD3 reverses the observed phenotypic alterations by repressing self‐renewal and stemness; promoting a more epithelial phenotype, reinstating neural maturation and differentiation; and halting tumor dissemination. Decisively, here we show that RD3 is a crucial regulator of NB lineage commitment, CSC dynamics, EMT‐MET balance, cellular identity and metastatic progression.

### 3.4 RD3‐Edited Cellular Identity Rewires Tumor Immunity

Expanding on our findings that RD3 critically regulates NB cell plasticity, RD3‐loss facilitate dynamic transitions towards stem‐like traits with self‐renewal sustenance, we investigated their contagion effect in NB tumor evolution. Pertinently, these phenotypic shifts may not merely compose cytoskeletal rearrangements, but rather represent a broader reprogramming of tumor microenvironment, biology, and function. Notably, we showed the significance of RD3 in shaping tumor immune microenvironment, tumor immune cell type composition, and immune surveillance [22]. Given this dual role in both lineage fidelity and immune regulation, here we investigated whether RD3‐reprogrammed cell‐identity translates to tumor immunogenicity.

#### 3.4.1 RD3 Bolster Tumor Cell Immunogenicity

Effective immune surveillance relies on the integrity of the MHC system, which enables lymphocytes to recognize and elicit an effective antitumor immune response. MHC I molecules present intracellular antigens to cytotoxic T_c_, while MHC II present antigenic peptides to helper T_c_. Dysregulation in expression/function of these molecules results in poor antigen presentation and presents an important mechanism of tumors escaping immune detection. Notably, loss of epithelial morphology (hallmark of cellular plasticity) has been linked to reduced immunogenicity. Given the RD3's role in preserving epithelial identity and suppressing phenotypic switching, we investigated whether RD3 attenuation compromises the antigen presentation machinery. Using our 12 RD3 systems approach, we first quantified the total protein expression of key antigen presentation determinants, HLA A/B, α‐chain of the MHC I molecule; HLA‐DPB1, encodes the β‐chain of MHC II; and β‐2‐Microglobulin (β2M), a subunit of MHC I. RD3 deficiency resulted in a remarkably consistent and cell‐line independent downregulation of HLA A/B expression underscoring a robust impairment of MHC I antigen presentation (Figure 6C,D). This collapse in immune visibility aligns with the heightened cellular plasticity observed in RD3^−/−^ systems, suggesting that plasticity driven by RD3 loss actively suppresses immune recognition. More importantly, the diminished levels of B2M, a crucial chaperone for MHC I complex stability, further affirms the disruption of immune visibility in RD3^−/−^ systems. Conversely, reinstating RD3 prompted a coordinated upregulation of both HLA A/B and β2M in all cell lines investigated, signifying a functional rescue of the antigen presentation machinery (Figure 6C,D). Interestingly, HLA DPB1 expression increases with RD3 loss and decreases in the presence of RD3 (Figure 6C,D). More importantly, to determine whether these alterations extend translationally to surface expression, we performed flow cytometry to quantify surface expression of HLA A/B/C and its partner β2M in RD3‐expressing Dx (CHLA‐15), RD3‐deficient PD (CHLA‐140), and their RD3 reverse‐engineered counterparts (CHLA‐15 RD3^−/−^ and CHLA‐140 RD3^+/+^). The readouts clearly displayed marginal HLA A/B/C and β2M surface expression in RD3‐deficient CHLA‐140 system, while restoring RD3 in these cells markedly reversed this phenotype by rearming the antigen presentation machinery (Figure S6A,B). Parallel experiments with RD3 expressing system showed high surface expression of HLA A/B/C and β2M, but a notable decrease when RD3 is silenced in these cells (Figure S6A,B). Together, these findings iterate that RD3 ensures that T_u_ remains within the radar of CD8^+^ T_c_ by promoting proper assembly and membrane localization of MHC I complexes. This plasticity‐driven evasion in immune detection designates RD3 as a crucial regulator of immune visibility, linking epithelial stability to effective immune recognition.

#### 3.4.2 RD3‐Loss Dictates Immune Checkpoint

In the core of NB evolution, RD3 loss emerges as an orchestrator of cellular plasticity, coordinating a symphony of molecular transformations that allow cells to evade immune surveillance. As RD3 fine‐tune the identity of T_u_, guiding them through EMT and back, it fuels a malleable phenotype primed for survival. This plasticity does more than shape shifting; it rewires the immune regulators to protect them from immune attack. Immune checkpoints (ICs) are critical negative regulators of immune homeostasis, preventing saturation and autoimmunity and maintaining self‐tolerance. While we observed a significant increase in ICs (PDL1, CD276) in RD3‐deficient and RD3‐muted cells, we observed a significant decrease in the expression of these ICs with RD3 restoration and/or in RD3‐expressing cells (Figure 6C,D). Findings from our RD3 muted models (CHLA‐15 RD3^−/−^, CHLA‐42 RD3^−/−^, SH‐SY5Y RD3^−/−^) undeniably exhibited strong phenotypic flexibility, which translated with increased expression of canonical immune checkpoint effectors PDL1 and CD276. This suggests that RD3 not only governs cell state transitions, but its loss negatively modulates immune activation, promoting NB immune evasion. On the contrary, reinstating RD3 reduced plasticity and led to a significant decrease in ICs, thereby enhancing both visibility and triggering the immunological cascade in NB (Figure 6C,D). Further, to dissect checkpoint dynamics, we assessed PDL1 co‐expression with MHC I at the cell surface. Our outcomes revealed that RD3‐silenced clones displayed a higher proportion of MHC I^+^PDL1^+^ cells compared to Dx‐RD3^+/+^ clone, indicating that RD3‐loss promotes IC engagement, potentially reinforcing an immune‐suppressive phenotype (Figure S6A,B).

#### 3.4.3 RD3 Regulated Immune Checkpoint is Mediated Through Inhibition of NFκB Signal Transduction

Owing to our observations that RD3 regulates PDL‐1, an immune checkpoint and a prognostic indicator after therapy in NB, here we examined the mechanism(s) involved. Since NFκB p65 is known direct transcriptional activator for PD‑L1, we investigated whether RD3 regulated PDL‐1 is mediated through the inhibition of NFκB transcriptional switch. For this, we used two independent RD3 deficient PD systems (CHLA‐90, CHLA‐20), with/without impeding NFκB signaling with Bay 11‐7082. Immunoblotting revealed high levels of cytoplasmic and nuclear p65, concomitant with increased PDL1 expression in both RD3‐deficient cells investigated (Figure S7). Blocking IκB phosphorylation completely abrogated both the cytoplasmic and nuclear abundance of p65. Crucially, this blockage of p65 in RD3‐deficient cells significantly reduced the PDL‐1 expression when compared with the untreated cells (Figure S7). These outcomes unveil not only that RD3‐deficient cells harbor high levels of nuclear and cytoplasmic p65 but critically demonstrate that RD3‐dependent regulation of PDL‐1 in NB could be mediated by altering the canonical NFκB signaling pathway. These findings point to a mechanistic link between cellular plasticity and immune stealth, recognizing RD3 as an upstream regulator in immune escape pathways.

#### 3.4.4 RD3 Regulates Tumor Antigens and Favors Immune Surveillance

In NB, RD3 selectively architect tumor plasticity. Strikingly, we observed that absence of RD3 led to an upregulation of GD2, a clinically relevant disialoganglioside heavily utilized as target in NB immunotherapy. Notably, RD3 deficient systems CHLA‐15 RD3^−/−^, CHLA‐42 RD3^−/−^, and SH‐SY5Y RD3^−/−^ increased GD2 expression alongside heightened expression of stemness and EMT drivers, suggesting that RD3 loss upregulates GD2 via cellular reprogramming (Figure 6C,D). Affirmingly, all RD3 expressing and restored systems showed a complete inhibition of GD2 (Figure 6C,D). Interestingly, the expression of CD171, another NB specific antigen remained unfazed despite the extensive transcriptional and phenotypic reprogramming of NB cells triggered by RD3 loss. This selective modulation hints a distinct regulatory axis, where GD2 is tightly linked to aggressive malleable cell state governed by RD3, while CD171 expression is more RD3‐independent (Figure 6C,D). Crucially these findings not only reveal differential control of antigen expression but carry critical clinical implications; tumors with RD3^−/−^ status may become more immunogenic due to GD2 overexpression, yet at the same time escape immunological detection through co‐upregulation of ICs, presenting a double‐edged sword in the context of NB immunotherapy.

#### 3.4.5 RD3‐Regulates Don't‐Eat Me Signaling: CD24 is at the Crossroads of Plasticity and Immune Evasion

CD24 acts as a molecular shield aiding immune evasion mechanisms, including suppression of T_c_ activation and proliferation, thereby contributing to an immune‐silent TME. To investigate the influence of RD3‐loss driven tumor plasticity in shaping the immune landscape of NB, we assessed the levels of CD24, a “don't eat me signal” in RD3 expressing, RD3‐reinstated, RD3‐deficient, and RD3‐muted systems. RD3‐deficiency and/or muting resulted in profound upregulation of CD24 (Figure 6C,D). This CD24 increase appears to be plasticity dependent, as RD3^−/−^ cells express higher levels of CD24 and corroborate the dictated EMT and dedifferentiation. On the other hand, RD3‐expressing and/or RD3‐restored systems, including CHLA‐20 RD3^+/+^, CHLA‐90 RD3^+/+^, and CHLA‐140 RD3^+/+^ exhibited pronounced differentiation as well as retained lower CD24 expression (Figure 6C,D). Together, this complete systems approach endorses RD3 as an upstream regulator and CD24 as an effector molecule that functionally emerges in response to an orchestrated identity crisis to prompt ‘do not eat me’ signal from cancer cells so as to dictate tumor immune evasion.

#### 3.4.6 RD3 Loss Drives Tumor Immune Evasion by Impairing T‐Cell Activation and Cytotoxicity

To determine whether RD3‐loss directed cellular plasticity translates into functional immune escape, we assessed the T_c_ capacity to carry out effector functions (TNFα, IFNγ secretion, activation) upon recognition of T_u_ and their cytotoxic capacity to kill. For this, T_c_ were cocultured (CoC) with RD3‐expressing Dx (CHLA‐15), RD3‐deficient PD (CHLA‐90) and their stably RD3 reverse engineered counterparts (CHLA‐15 RD3^−/−^ and CHLA‐90 RD3^+/+^). Baseline cytokine profiling revealed RD3‐status dependent TNFα and IFNγ secretion by T_u_ (Figure 6E,F; Figure S8A). T_c_ produced negligible cytokines under unstimulated conditions (Figure 6E,F; Figure S8A). Following CoC, T_c_ cytokine secretion was strongly induced by RD3‐proficient CHLA‐15 T_u_, while RD3‐deficient T_u_ (CHLA‐15 RD3^−/−^) negatively affected T_c_ TNFα and IFNγ production (Figure 6E,F; Figure S8A). Conversely, while T_c_ CoC with the RD3‐deficient PD T_u_ (CHLA‐90 RD3^−/−^) displayed impeded cytokine secretion, this compromise was strikingly rescued (cytokine secretions comparable to RD3‐proficient conditions) upon RD3 restoration (CHLA‐90 RD3^+/+^, Figure 6E,F; Figure S8A). These findings affirm RD3's critical role in promoting tumor immunogenicity. Extending our observations into functional activation, we measured the expression of CD107a (Lysosome‐associated membrane protein 1), a marker of degranulation and cytotoxic granule, and CD137, a late activation marker indicative of antigen‐specific engagement and persistence on T_c_. Under unstimulated conditions, T_c_ displayed low surface expression of CD107a and CD137 (Figure 6E,G; Figure S5B). Presence of T_u_ induced robust activation of T_c_ as evidenced by the upregulation of both markers. T_c_ CoC with RD3‐reinstated CHLA‐90 RD3^+/+^ clones showing pronounced expression of CD107a, (compared to the Tc CoC with the parental RD3‐deficient CHLA‐90 RD3^−/−^ T_u_), indicating RD3‐dependent effective cytotoxic release. On the other hand, T_c_ CoC with RD3‐knock down CHLA‐15 RD3^−/−^ T_u_ displayed (vs. Tc CoC with the parental RD3 expressing CHLA‐15 T_u_) reduced Tc activation (CD137) (Figure 6E,G; Figure S8B). These findings iterate that RD3‐loss compromises Tc activation at both early and late stages, while restoration promotes robust sustained cytotoxic response. Crucially to ascertain RD3‐dependent immune‐mediated tumor susceptibility, we performed tumor killing assays following 48hr CoC. All T_u_ systems showed remarkable increase in caspse‐3 activity, confirming the relevance of T_c_ mediated cytotoxicity in driving T_u_ death (Figure 6E,H; Figure S8C). Notably, this T_c_ cytotoxic effect was directly proportional to T_u_‐RD3 status. RD3‐expressing Dx and RD3‐reinstated PD T_u_ exhibited high caspase activity and greatest reduction in viable cells, indicating susceptibility to immune‐mediated killing (Figure 6E,H; Figure S8C). In contrast, RD3‐deficient CHLA‐90 T_u_ and RD3‐null CHLA‐15 RD3^−/−^ T_u_ retained larger fraction of viable cells consistent with immune‐evasive phenotype (Figure 6E,H; Figure S8C). These outcomes collectively affirm that RD3 is essential for effective T_c_ activation, engagement; and enhancing tumor susceptibility to immune attack.

Taken together, these mechanistic studies identified that RD3 is the critical regulator of T_u_ immunogenicity, and its loss with therapy pressure dictates immune checkpoint and tumor immune evasion. More importantly, these outcomes corroborate with the RD3‐regulated cellular plasticity outcomes and imply that acquired RD3‐loss with clinical therapy pressure governed CSC identity and burden crucially contributes to the composition of tumor immune evasion.

### 3.5 RD3 construe Tumor Immune Milieu In Vivo

Owing to the mechanistic insights on the RD3‐directed control of cellular plasticity and immune evasion, it is critical to understand the translation of function in real time in vivo. To that end, it is pertinent to understand the regulatory role of RD3 in the sequential evolution of TIME by assessing (i) immune equilibrium; (ii) adenosinergic immune suppression; (iii) cytotoxic T_c_ infiltration; and (iv) T_u_ killing. For this, we evaluated the tumors for RD3‐dependent fluctuations in shaping the tumor immune microenvironment during disease progression pseudo‐metastatic disease developed in mice with five systems, including (a) RD3^+/+^ Dx metastatic site derived CHLA‐42; RD3^−/−^PD (b) CHLA‐20; (c) CHLA‐90; RD3 restored PD systems (d) CHLA‐20 RD3^+/+^ and (e) CHLA‐90.

#### 3.5.1 RD3 Governs Tumor Immune Equilibrium

**FIGURE 7:**
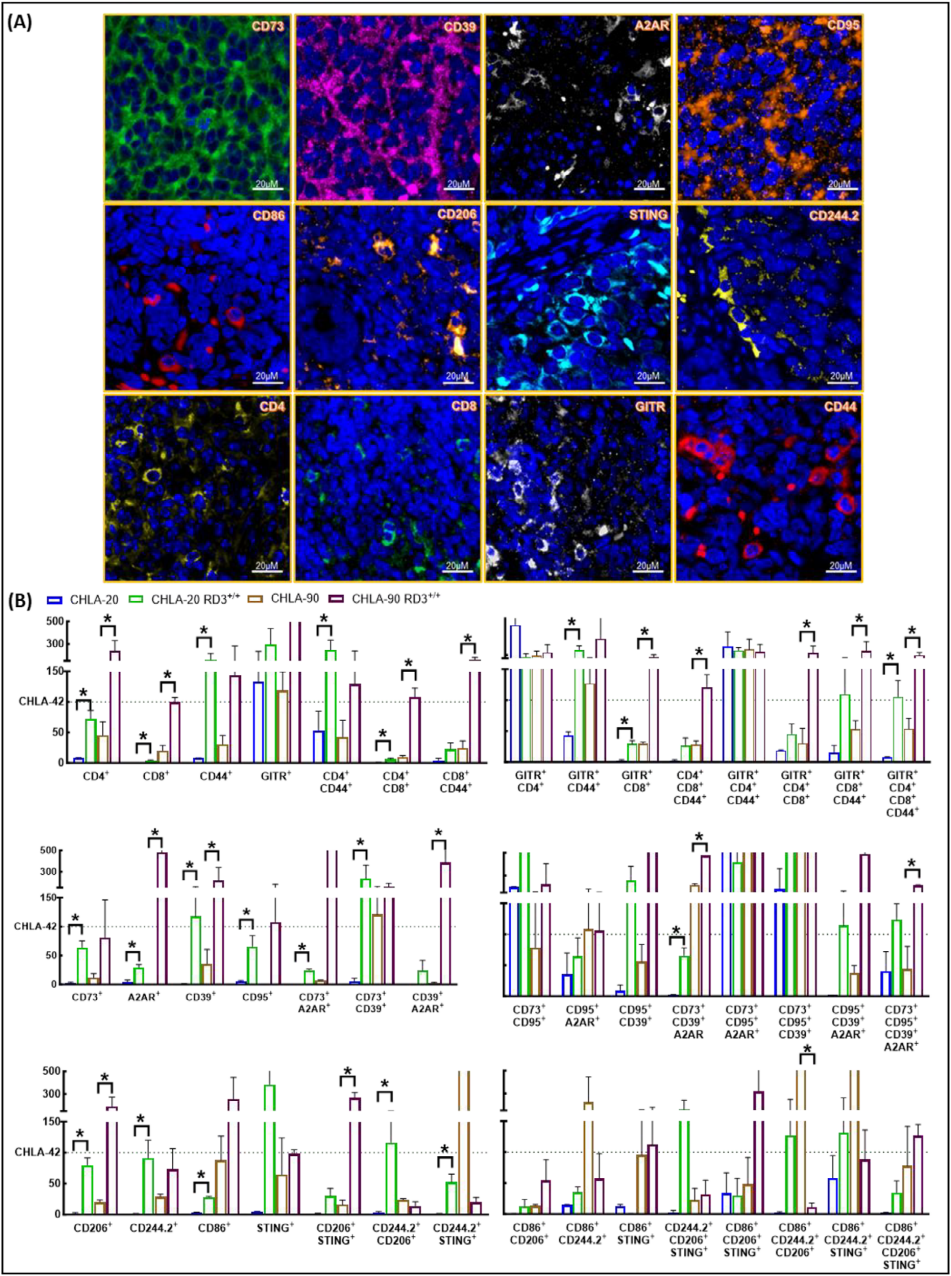
RD3‐loss dictates immune defense barrier (A) Multi‐IHF analysis of tumor sections across a panel of five distinct NB models in vivo derived from RD3‐expressing CHLA‐42, RD3‐deficient CHLA‐20, CHLA‐90, and RD3‐reexpressed CHLA‐20 RD3^+/+^ and CHLA‐90 RD3^+/+^ systems. Tumor sections were stained for curated panel of immune modulatory markers of (i) effector subsets CD4, CD8 (ii) activation CD44, GITR (iii) purinergic immunosuppression axis CD39, CD73, A2AR (iv) immune sensing and antigen presentation CD86, STING, CD206 (v) immune exhaustion CD244.2, and (vi) apoptosis CD95. Representative sections are presented at 20X magnification, and scale bars represent 20 µM. (B) Quantitative analysis of immune marker expression across all experimental conditions comparing RD3‐reinstated, RD3‐expressing and RD3‐null derived tumors. Data is organized into three functional panels reflecting immune activation, suppression and surveillance dynamics. Top panel focusses on functional polarization and activation. RD3^+/+^ tumors exhibited increased infiltration of CD4^+^ and CD8^+^ effector T_c_ compared to RD3‐null counterparts, indicating improved immune engagement. Frequency of co‐expression of CD44 and GITR on effector subsets (CD8^+^GITR^+^, CD4^+^GITR^+^, CD8^+^CD44^+^, CD4^+^CD44^+^, CD8^+^CD44^+^GITR^+^, CD4^+^CD44^+^GITR^+^) increased in RD3^+/+^ systems reflecting improved tumor immunogenicity and surveillance. Middle panel represents adenopurinergic axis mediated immune suppression and apoptotic regulation. RD3‐null tumors showed elevated co‐expression of CD39, CD73, and A2AR consistent with activation and engagement of immune suppressive and tolerogenic mechanisms promoting immune escape in NB. In contrast, RD3‐reinstated systems promoted CD95 expression on tumors, implicating RD3s’ role in immune‐mediated apoptosis and tumor clearance. The bottom panel shows immune surveillance (CD206, CD86, STING) and exhaustive landscape (CD244.2) in NB tumors. RD3‐reinstated tumors demonstrated reduced expression of immune exhaustive phenotype alongside elevated levels of STING, CD86, and CD206 in immune cells compared to RD3‐null tumors. This reinforces RD3's role in restoring immune visibility and surveillance.

CD206, a mannose receptor, is recognized for its anti‐tumor functions, promoting immune surveillance. Compared to the RD3^+/+^ Dx CHLA‐42 tumors, we found a complete loss of CD206 in both RD3^−/−^PD tumors (Figure 7A,B). However, in RD3‐restored PD systems, CHLA‐20 RD3^+/+^ and CHLA‐90 RD3^+/+^, significantly heightened CD206 expression, and corroborated the increased infiltration of APCs and enhanced immune activation signature in these tumors. Likewise, we observed a significant reversal of RD3‐deficiency dependent loss of CD86, a co‐stimulatory molecule for T_c_ activation when RD3 is restored in these PD systems (Figure 7A,B). These findings were further substantiated by elevated co‐expression of Stimulator of Interferon Genes (STING), an intracellular protein involved in immune surveillance, indicating a shift towards a pro‐inflammatory state. We found a significant elevation in STING^+^, CD206^+^STING^+^ cells in RD3‐reinstated system CHLA‐20 RD3^+/+^ and CHLA‐90 RD3^+/+^, indicating an anti‐tumor immune reprogramming enhancing immune recruitment and response. Further, CD206^+^CD86^+^ hybrid phenotype was profoundly enriched in RD3‐reexpressed systems, supporting enhanced antigen presentation capabilities co‐stimulation of cytotoxic T_c_. The diminished CD206^+^, STING^+^, CD86^+^, CD206^+^STING^+^ and CD206^+^CD86^+^ subsets in RD3‐deficeint CHLA‐20, CHLA‐90 NB systems directly reflect an immune suppressed landscape. Complementing these outcomes, we found a remarkable elevation of CD86^+^CD206^+^STING^+^ population in CHLA‐90 RD3^+/+^ compared to RD3‐intact CHLA‐42, indicating that these subsets might serve as immune organizers enhancing immune visibility and response. CD244.2, a receptor implicated in immune exhaustion, displayed a tumor‐specific response with RD3 restoration, directing an increase in CHLA‐20 tumors and a decrease in CHLA‐90 tumors (Figure 7A,B). Interestingly, this tumor‐dependent RD3 response trend stands true for all CD244.2^+^CD86^+^; CD244.2^+^CD206^+^ and CD244.2^+^STING^+^ dual positive populations, indicating tumor character (e.g., primary to BM) specific immune modulation. Although the precise functional role of these subsets remains to be fully defined, our findings indicate that their increased prevalence may skew toward an anti‐inflammatory response, attenuating T_c_ activation. Triple‐positive subsets revealed a much more complex immune state, and we observed distinct expression patterns in our RD3 systems. CD244.2^+^CD206^+^STING^+^ and CD206^+^CD86^+^STING^+^ showed a remarkable increase after forced RD3 expression in CHLA‐20 RD3^+/+^ and CHLA‐90 RD3^+/+^, while CD244.2^+^CD86^+^STING^+^ and CD244.2^+^CD206^+^CD86^+^ showed a more robust cell line‐dependent response (decreased CHLA‐90 and increased in CHLA‐20 upon RD3 restoration).

#### 3.5.2 RD3 Deters Adenosinergic Tumor Immune Suppression

To investigate the immune suppressive mechanism driven by RD3‐loss, we profiled key components of the purinergic signaling axis: CD39, a cell surface ectonucleoside triphosphate diphosphohydrolase; CD73, a cell surface ecto‐5‐nucleotidase catalyzing AMP to adenosine and A2AR, a G‐protein coupled receptor activated by adenosine. Compared to the Dx RD3^+/+^ CHLA‐42 tumors, RD3^−/−^ PD systems, CHLA‐20 and CHLA‐90 displayed a robust increase in CD73 and CD39 expression. Conversely, re‐expressing RD3 significantly inhibited CD73 in both PD tumors and CD39 in CHLA‐20 PD tumors (Figure 7A,B). (CHLA‐20) RD3 response. Consistently, A2AR expression on immune cells was markedly elevated in RD3‐null PD (Figure 7A,B; vs. Dx RD3^+/+^ CHLA‐42 tumors). This combined upregulation of CD73, CD39, A2AR upon RD3‐loss reprograms the TME toward metabolic immune suppression. In contrast, RD3‐reexpressed systems CHLA‐20 RD3^+/+^ and CHLA‐90 RD3^+/+^ showed coordinated decrease in all three purinergic signaling molecules CD39, CD73, A2AR, suggesting ablation of adenosine‐mediated immune silencing. Building on these findings, RD3‐deficiency was found to significantly enrich dual positive populations within PD state. Specifically, CD73^+^CD39^+^ cells, which possess full enzymatic machinery to convert extracellular ATP to adenosine were significantly elevated in RD3‐null cells compared to RD3^+/+^ CHLA‐42. Similarly, cells capable of producing and responding to adenosine (CD73^+^A2AR^+^) were significantly upregulated in RD3‐deficient systems CHAL‐20 and CHLA‐90, reinforcing autocrine immune suppression. Although, displaying a tumor‐dependent profile, the CD39^+^A2AR^+^ population is known to initiate and enable paracrine signaling. In stark contrast, RD3‐restoration reversed all dual positive intermediate stages of purinergic signaling, including CD39^+^A2AR^+^, CD73^+^A2AR^+^, CD73^+^CD39^+^ within TME in both PD systems investigated. This reversal underscores RD3's role as a negative regulator of purinergic signaling and adenosine‐mediated immune silencing. More importantly, our multiplex IF studies revealed co‐expression of CD73/CD39/A2AR in RD3‐null CHLA‐20 and CHLA‐90 NB models, further reinforcing a self‐sustaining immunosuppressive loop driven by RD3‐loss in NB. Taking together, RD3‐loss not only increases individual marker expression but also promotes co‐expression of adenosinergic effectors, thereby amplifying immune evasion.

#### 3.5.3 RD3 prompts T‐cell activation and surveillance

Building on our previous findings that RD3 critically alters immune cell composition within NB tumors [22], we sought to characterize the functional consequences of RD3‐loss on T_c_ subset activation and their surveillance. Using multiplex IF profiling, we examined the immune landscape across RD3‐deficient (CHLA‐20, CHLA‐90), RD3‐restored (CHLA‐20RD3^+^, CHLA‐90 RD3^+/+^), and RD3‐expressing (CHLA‐42) NB ex vivo systems. CD4^+^ and CD8^+^ T_c_ abundance showed a consistent and significant increase in RD3‐restored systems, indicating the function of RD3 in adaptive immune surveillance, promoting immune cell infiltration and/or retention within the TME. Strikingly RD3‐deficient systems exhibited a relative increase in CD4^+^ helper T_c_ and a concomitant reduction in CD8^+^ cytotoxic T_c_ when compared to RD3^+/+^ CHLA‐42 (Figure 7A,B). This deranged CD4/CD8 ratio in RD3^−/−^ CHLA‐20 and CHLA‐90 reflects compromised cytotoxic response and skewness in the adaptive immune compartment. To dissect the functional status of T_c_, we profiled the expression of CD44, a transmembrane glycoprotein involved in T_c_ activation and memory formation. RD3‐deficient systems exhibited a reduction in both CD8^+^CD44^+^ and CD4^+^CD44^+^ T_c,_ reflecting impaired cytotoxic activation and diminished support for cytotoxic T_c_ priming. In parallel, Glucocorticoid‐induced TNFR‐Related protein (GITR), a co‐stimulatory receptor expressed on activated T_c_ was profoundly reduced in both RD3^−/−^ PD (CHLA‐20, CHLA‐90) systems compared to RD3^+/+^ systems. Importantly, RD3 restoration reversed these effects by upregulating both CD44 and GITR, reinforcing immune readiness. In addition, expression of GITR in CD4 and CD8 T_c_ subsets displayed a significant shift in activated CD8^+^GITR^+^ T_c_ subset in both RD3‐reinstated CHLA‐20 RD3^+/+^ and CHLA‐90 RD3^+/+^ system, while CD4^+^GITR^+^ showed a marginal tumor‐dependent response with RD3 restoration (Figure 7A,B). Strikingly, RD3‐deficient systems exhibited a significant depletion of CD8^+^CD44^+^GITR^+^ triple‐positive population, indicative of highly inactivated and functionally incompetent cytotoxic T_c_. This triple‐positive population was actively restored in both RD3‐restored CHLA‐20 RD3^+/+^ and CHLA‐90 RD3^+/+^ systems. In parallel the CD4^+^CD44^+^GITR^+^ helper T_c_ population showed minimal effect upon RD3 reinstatement. Conformingly, these results underscore RD3's role in T_c_ activation, survival, and effector function. Beyond the conventional CD4^+^ and CD8^+^ T_c_ subsets, our multiplex IF revealed the presence of CD4^+^CD8^+^ double positive subset (Figure 7A,B). In RD3‐null systems CHLA‐20 and CHLA‐90 the dual population was remarkedly reduced while RD3 expression led to robust expansion of CD4^+^CD8^+^ translational T_c_ subset within the TME. Further, this expansion was accompanied by a coordinated upregulation of CD44 and GITR in RD3 reinstated stated systems, leading to increased presence of CD4^+^CD8^+^CD44^+^, CD4^+^CD8^+^GITR^+^, and CD4^+^CD8^+^CD44^+^GITR^+^ populations compared to their RD3‐null counterparts. Collectively, here we show that RD3‐loss disrupts cytotoxic T_c_ priming and impairs immune surveillance while its restoration, not only reverses the immune suppressive phenotype, but also reinforces the functional competence of immune subsets.

#### 3.5.4 RD3 Regulates CD95 to Promote Apoptosis

CD95, a cell surface receptor known as Fas, plays a central role in regulating apoptosis, and we found its dynamic co‐expression with adenosinergic markers CD39, CD73, and A2AR within NB‐TME. As a single marker, CD95 expression is elevated in RD3‐reinstated CHLA‐90 RD3^+/+^, showing a tumor‐dependent response for the activation of apoptosis. However, beyond its canonical role, CD95 co‐expression with purinergic molecules CD39, CD73, A2AR exhibits a more potent immune suppressive phenotype. Dual positive populations CD95^+^CD39^+^, CD95^+^CD73^+^, and CD95^+^A2AR^+^ were significantly enriched in RD3‐null systems (vs. RD3^+/+^ CHLA‐42), while stable restoration of RD3 reduced the enrichment of such dual positive populations within the NB‐TME. These dual positive populations direct the induction of apoptosis in activated immune cells or promote survival and immune evasion in T_u_. While RD3‐loss promotes the enrichment of these metabolic and apoptotic suppressive niches within TME, RD3‐reinstatement significantly decreased CD95^+^CD73^+^, CD95^+^A2AR^+^, while marginally reduced CD95^+^CD39^+^ population in CHLA‐20 RD3^+/+^ and CHLA‐90 RD3^+/+^ systems.

Overall, the results demonstrate that RD3 reinstatement coordinates a comprehensive immune reprogramming involving both innate and adaptive immune compartments. Enhanced infiltration of CD4^+^ and CD8^+^ T_c_ was accompanied by expression of co‐stimulatory molecules CD44^+^ and GITR^+^. Concurrently, the innate immune compartment was reprogrammed in RD3‐reexpressed systems, reflecting enhanced antigen presentation, interferon signaling, and support T_c_ activation through elevated expression of CD206, CD86, and STING. In contrast, CD244.2 was significantly downregulated in RD3‐restored systems, correlating with improved cytotoxicity and T_u_ killing. Simultaneously, the suppression of CD39, CD73, and A2AR in RD3‐reinstated systems indicated attenuation of immune suppressive TME, favoring immune persistence and function. These findings collectively underscore RD3's role in definitively restoring immune equilibrium, enhancing visibility, and promoting antitumor immunity.

## 4 Discussion

Cellular plasticity has its roots deep in developmental biology, endowing cells with the remarkable ability to traverse differentiation pathways and commit to specialized developmental lineages [2]. Yet, this same inherent flexibility, once orchestrating orderly development, becomes a liability in cancer. Under therapeutic and environmental stress, phenotypic plasticity enables cells to adapt and survive a hostile environment, enabling cancerous cells to reprogram its identity and promote disease progression [2, 34]. This adaptive reprogramming enables cancer cells to escape lineage constraints and transform into functionally overlapping processes, including EMT, trans‐differentiation, and acquisition of stemness traits that collectively drive tumor evolution [2, 35]. More importantly, this genetically independent mechanism not only facilitates ‘hide and seek’ but actively remodels the TME, fostering immunosuppression and limiting immune control over tumor [2, 12]. Studies have revealed that T_u_ undergoing cellular disguise actively modulates antigen presentation, rewire immune checkpoint regulation, and skew immunogenic landscape of tumors [10, 12, 36]. The communique between the phenotypically versatile T_u_ and immune compartment not only facilitates tumor survival but also accelerates immune evasion by reshaping the immune microenvironment into a sanctuary for malignant progression. Despite growing insights into the consequences of cellular plasticity, our understanding of the regulatory architecture of cellular plasticity and the mechanism of plasticity orchestrated immune remodeling are far from complete. Unraveling the regulatory determinant that initiates and sustains plasticity‐driven immune escape remains not only critical for decoding tumor adaptability but also holds potential to expose unexpected vulnerabilities that can be strategically targeted in cancer cells.

RD3 is a ubiquitous (cytosolic, perinuclear, and nuclear) small protein (∼23 KDa), whose loss causes photoreceptor degeneration and blindness in humans, termed as Leber's congenital amaurosis [37]. In retina, RD3 competitively binds to guanylate cyclases (RetGCs) that not only facilitate GCs expression and stability, but are also crucial for restoring GCs localization (trafficking GCs to outer segment), together ensuring photoreceptor cell survival [23]. The RetGC binding domain of RD3 is hypothesized to reside within RD3's central region. Although the retinal function of RD3 is well characterized, and owing to our findings that RD3 is constitutively expressed in all fetal and adult human tissues, its defined physiological roles beyond the retina remain unexplored. Our recent studies critically revealed that therapy‐pressure driven RD3‐loss is not a passive consequence but a de novo regulatory shift that synchronizes tumor progression in therapy‐defying HR‐NB [18, 22, 24, 25, 27, 30, 32]. Unearthing the relevance of the drug, if any, this study outcomes clearly iterated that the acquisition of RD3 loss is more a principal event in response to therapy pressure, rather than an exclusive response to a specific drug. This is consistent with our earlier report that NB cells resistant to current pipeline drugs (e.g., ALK inhibitor TAE‐684, BET bromodomain inhibitor IBET‐726, CDK7 inhibitor THZ1 and IGF‐1R antibody H10H5) display significant RD3 loss [18]. Grounded by this evidence and considering RD3's canonical role, we hypothesized that RD3 acts as a molecular checkpoint that stabilizes cellular identity and constrains plasticity. This hypothesis is strongly supported by evidence from our findings showing RD3 depletion dismantles lineage fidelity, enabling EMT, dedifferentiation, and stemness acquisition, while simultaneously reprogramming the tumor immune microenvironment toward immune evasion. Consistent with this, RD3 loss correlates with poor survival in other solid cancers (unpublished data), reinforcing its potential as a universal regulator of tumor adaptability. By positioning RD3 at the intersection of cellular plasticity and immune control, herein our findings mechanistically demonstrate how RD3 governs plasticity‐driven immune escape in NB, establishing its critical role in lineage stability and immune visibility.

Our unique RD3 reverse‐engineered systems developed tumors in vivo in this study, for the first time, demonstrated that RD3‐deficient tumors sequentially bring into play lineage transformation through activation of EMT, dedifferentiation, and CSC enrichment, underscoring a profound shift in NB cell identity. These findings designate RD3 as a molecular checkpoint that constrains malignant plasticity. While our previous studies have decisively implicated EMT and stemness in T_u_ plasticity and aggressive tumor behavior, findings from this study exclusively unveiled the function of RD3 in their integrated and/or coordinated rearrangements, their functions in the amplification of evolutionary competence, and therapeutic resistance. By and large, these findings affirm that RD3 functions as a stabilizer of lineage transformation, and its loss enables NB to exploit multiple adaptive trajectories. More importantly, RD3‐silencing not only inflicts molecular mayhem, but assuredly deranged the T_u_ architecture, prompting the development of tumorosphere, 3D aggregates that are functional surrogates of enhanced self‐renewal and stemness. Remarkably, this structural remodeling was accompanied by RD3‐loss dependent establishment and maintenance of pluripotency triad (SOX2, NANOG, OCT3/4), reinforcing the RD3‐loss initiates a comprehensive reprogramming towards a highly adaptable state. Critically, our RD3 pull‐down and co‐IP analysis in parental and their RD3 reverse engineered systems undeniably recognized its cell‐line independent binding to all three pluripotency core circuitry triad, identifying the functional applicability of RD3‐loss in the plasticity setting. Diving in deep, this study laid the first stone defining the direct RD3 mechanism of action when our ChIP‐qPCR analysis disclosed the RD3‐dependent promoter activation of these critical determinants of plasticity. While this evidence is unambiguous, how RD3 principally sequesters SOX2, NANOG, and OCT3/4 and the precise regulatory architecture remains unexplored. At this point, we could speculate three non‐exclusive mechanisms that RD3 could exploit including: (i) acting as a chromatin modulator to influence chromatin architecture and promoter accessibility; (ii) function as a condensate modulator to destabilize *SOX2‐OCT3/4‐NANOG* nuclear condensates and associated cofactors [38, 39] and; (iii) exerting mutual antagonism by competing with other transcriptional regulators for promoter occupancy [40]. Decoding these regulatory layers is a requisite, as they highlight that RD3's influence on pluripotency is not an isolated event but a foundational process that dictates the stemness landscape. This upstream control over pluripotency likely contributes to the enrichment of CSC niches, which we observed as enrichment of dual and triple‐positive populations (CD133^+^, CD114^+^, CD117^+^) upon RD3 depletion. Rather than simply expanding the stem‐like pool, RD3‐diminution drives the emergence of rare, aggressive CSC niches that contribute to relapse and therapeutic resistance. Crucially, our findings indicate that RD3 does not simply suppress stemness, rather it governs the architecture and composition of stemness ecosystem.

Beyond its role in driving lineage fidelity, plasticity exerts profound influence on immune regulation and shaping the TME into an immune evasive niche. Plasticity mediated immune regulation has emerged as a defining axis of cancer progression in solid tumors including NB, where dynamic shifts in cellular state actively reprogram the tumor immune microenvironment to favor immune escape [34]. This adaptive remodeling influences the trajectory of cancer immunoediting, enabling T_u_ to evade immune detection, suppress effector response and establish immunosuppressive niches. Such lineage deviation is frequently accompanied with reduced antigen presentation, enhanced ICs, resistance to cytotoxic lymphocyte mediated clearance and expansion of immune suppressive subsets [12]. These immune‐evasive tumor clones exploit their phenotypic flexibility to persist under therapeutic and immunological pressure. Despite these realizations, the deterministic regulators that initiate and sustain plasticity‐immune escape remains poorly defined. Breaking new ground, our findings position RD3 as a critical orchestrator of this axis. The transition towards stem‐like, mesenchymal phenotypes in RD3‐deficeint NB coincided with a collapse of immune visibility, marked by downregulation of MHC class I components (HLA A/B, β2M) and induction of ICs (PDL1, CD276). Conceptually, this impairment dismantles antigen presentation and heightens immune suppression, directly undermining the elimination phase of immunoediting and enable tumor clones to persist under immune pressure. Substantiating this, our outcomes identified that RD3 exerts a NFκB‐dependent regulation of immune checkpoint, a key mechanistic link between cellular plasticity and immune stealth, recognizing RD3 as an upstream regulator in immune escape pathways. The mesenchymal state further reinforces the immune escape in RD3‐deficient tumors through CD24 upregulation, a “don't‐eat‐me” signal and selective GD2 enrichment, a clinically actionable NB antigen. Crucially our findings indicated that these changes were not confined to T_u_ but extended to the immune compartment. Essentially, we showed that RD3 controls T_c_ effector responses, including cytokine secretion, activation, and their cytotoxic function. RD3‐deficeint tumors exhibited skewed T_c_ composition, depletion of activated T_c_ effectors, revitalize purinergic signaling and establish a self‐sustaining adenosine‐mediated escape loop. Collectively our findings reveal that RD3‐loss not only permits immune escape but actively engineers an immune‐silent ecosystem through coordinated modulation of antigen presentation, checkpoint signaling and effector cell dynamics. To our knowledge, these outcomes for the first time unveil RD3 as a central regulator of cancer immunoediting wherein RD3‐governed cellular plasticity dictates immune visibility and functional engagement, shaping tumor clearance in NB.

Altogether, this compelling evidence clearly establishes the decisive function of RD3 in regulating cellular plasticity and plasticity driven TIME in NB. However, the authors are aware and acknowledge the limitation that, while the outcomes iterate the RD3‐deficiency translated cellular and immunological remodeling as well in shifting T_c_ composition, antigen presentation, and activation, it is critical to determine whether tumor‐targeted RD3 restoration can reverse cellular plasticity and immune escape in vivo. Also, the authors acknowledge the limitations that tumor sphere formation assay cannot replace the gold standard in vivo limiting dilution assay quantifying tumorigenic efficiency. However, our outcomes now provide a strong rationale to test the hypothesis and to develop molecular targeted countermeasures in translatable transgenic, spontaneous, and syngeneic preclinical models. Likewise, the authors acknowledge the limitations of the mouse model used and the requirement of clinically translatable model to investigate endogenous immune response. Studies are underway in our laboratory in transgenic models with human syntenic disease and experimental mouse models of primary and metastatic disease to evaluate the therapeutic potential of tumor targeted RD3‐reinstatement. Likewise, the authors also recognize that RD3 deficiency plasticity and TIME effects could be partially managed by compensatory pathways and signaling mechanisms. However, our ex vivo and in vivo studies comprehensively covered interlaced core machineries involved in EMT, stemness, pluripotency maintenance, differentiation, metastasis, immune activation, and surveillance, and TIME, indicating that additional alternate pathways/compensatory signaling may not negate RD3 deficiency effects. With these cutting‐edge realizations, our current focus is centered on unearthing RD3's functional partners and signaling networks that facilitate cellular plasticity and TIME, or those compensatory/negatory elements, if any, through high throughput, yet directed proteomic and metabolomic strategies. Integrative bioinformatics involving transcriptomics, proteomics, phosphoproteomics etc., will not only unveil the steered genetic and molecular blueprints but will exemplify RD3‐centered core circuits and uncover actionable vulnerabilities. While our findings in this study identified that RD3 is lost in surviving cells almost for every single chemo drug tested, we are actively addressing the crucial gap of how clinical therapy pressure instigates RD3‐loss, and the mechanisms (as well the signal transduction flow‐through) involved, the key requisites of realizing clinical relevance and developing targeted maintenance therapy.

In conclusion, this vanguard study designates RD3 as a central orchestrator that governs the interplay of cellular plasticity and TIME in NB, redefining its role from a retinal protein to a critical checkpoint in cancer evolution. For the first time, this study defines the promoter repressor function of RD3 for at least for pluripotency core circuitry triad, starring its physiological function beyond retina. Therapy‐induced RD3‐loss emerges as a dominant driver of malignant adaptability and cancer immunoediting. By rewiring the transcriptional and translational programs governing cellular plasticity, RD3‐dimunition constructs a tumor architecture optimized for survival–enriched CSC niches, EMT driven metastatic competence, clonal selection and expansion all while simultaneously constructing an immune‐silent ecosystem. These coordinated events transform NB into a highly adaptable, immune‐cold tumor. By establishing this mechanistic axis, here we provide an absolute framework that explains how therapy‐pressure directed RD3‐loss defy lineage commitment; and governs cancer immunoediting. Owing to the fact that RD3 emerges as a powerful biomarker for aggressive phenotypes and immune‐cold tumors, the findings of this study critically provide rationale and open new venues for the development of targeted maintenance therapeutic strategies for immune‐cold progressive NB.

## Author Contributions

N.A. contributed to the conception and design of the experiments. P.S., S.M., L.P., S.S.N., A.J.H., N.A., and S.A. performed the experiments and contributed to the acquisition of the data. S.M., S.A., and A.J.H. performed all Digital Pathology scanning. S.A., P.S., and S.M. performed all histological processing and multiplex‐IF on tumors sections. P.S., A.J.H., S.S.N., and S.S. performed the acquisition of multiplex imaging data. N.A., P.S., S.M., L.P., and S.A. contributed to data analysis and interpretation of the data. P.S. and N.A. drafted the manuscript, and S.A., S.M., and L.P. helped in revising it critically. All authors read and approved of the final manuscript.

## Funding

This work was funded by Department of Defense, DoD CA‐210339; Kerr Foundation Funding, Kerr Foundation 28‐34200; Oklahoma Center for the Advancement of Science and Technology, OCAST‐HR19‐045; and The National Institutes of Health P20GM103639.The work was also supported by the National Cancer Institute Cancer Center Support Grant [P30CA225520, and a grant from the Oklahoma Tobacco Settlement Endowment Trust [R23‐03] both awarded to the OU Health Stephenson Cancer Center.

## Ethics Approval

All animal experiments conformed to American Physiological Society standards for animal care and were conducted following guidelines from the National Research Council. All the protocols used were approved by the Oklahoma State University Institutional Animal Care and Use Committees (IACUC‐23‐42) and the University of Oklahoma health science center (IACUC 20‐012‐CI). All animal studies complied with institutional guidelines on handling laboratory animals as well as all appropriate state and federal regulations.

## Consent

The authors have nothing to report.

## Conflicts of Interest

The authors declare no conflicts of interest.

## Supporting information




**Supporting File**: advs74148‐sup‐0001‐SuppMat.docx.

## Data Availability

All data generated or analysed during this study are included in this published article and as supplemental information.
