# Supplementary material for: Cellular Identity Crisis: RD3 Loss Fuels Plasticity and Immune Silence in Progressive Neuroblastoma
Source: Adv Sci (Weinh). 2026 Jan 31;13(20):e19586. doi: 10.1002/advs.202519586 (PMC13067867; doi:10.1002/advs.202519586)
Supplement: Supplementary file 1 — Supporting File: advs74148‐sup‐0001‐SuppMat.docx. [file ADVS-13-e19586-s001.docx]

**CELLULAR IDENTITY CRISIS: RD3 LOSS FUELS PLASTICITY AND IMMUNE SILENCE IN PROGRESSIVE NEUROBLASTOMA**

*^1^Poorvi Subramanian, ^1^Sreenidhi Mohanvelu, ^2^Sheeja Aravindan, ^1^Afsana Parveen Jahir Hussain, ^1^Sivasubramani Narayanan, ^1^Sabir Salim, ^1^Loganayaki Periyasamy and ^1, 2^Natarajan Aravindan^*^*

^1^Department of Physiological Sciences, Oklahoma State University, Stillwater, OK, USA

^2^OU Health Stephenson Cancer Center, Oklahoma City, OK, USA.

**Short title:** RD3: A facet of tumor cell plasticity and immune synergy

**Corresponding author:**

Natarajan Aravindan Ph.D.

Professor, Department of Physiological Sciences

College of Veterinary Medicine, Oklahoma State University

160 McElroy Hall

208 N McFarland St, Stillwater, OK 74078, USA.

Voice: (405) 744-6292

Email: [natarajan.aravindan@okstate.edu](mailto:natarajan.aravindan@okstate.edu)


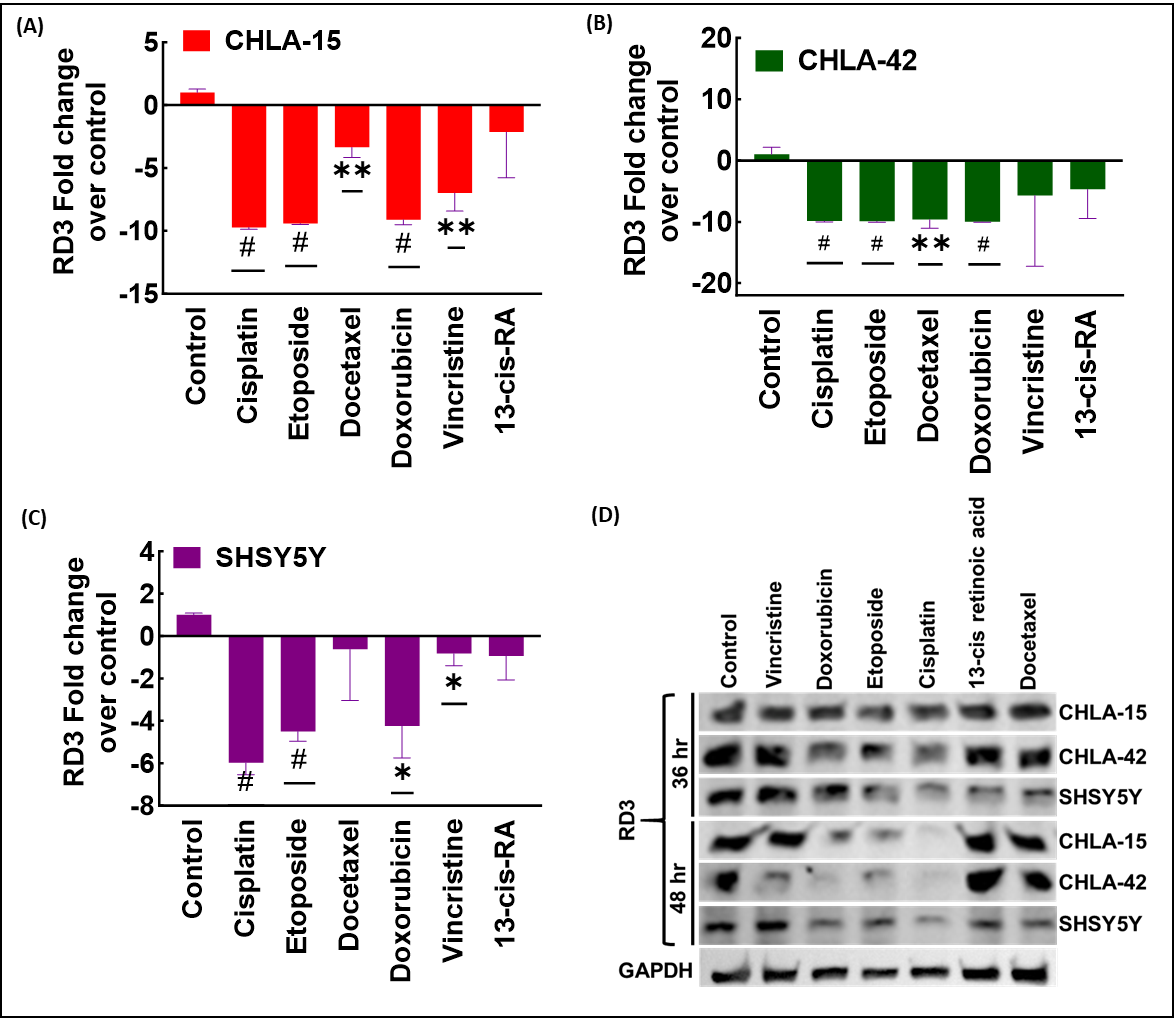


**Figure S1:** Histograms from qPCR analysis showing complete transcriptional loss of RD3 across all Dx-RD3^+/+^, (**A**) CHLA-15, (**B**) CHLA-42, and (**C**) SH-SY5Y cells treated with vincristine (0.1 µM), doxorubicin (1 µM), cisplatin (10 µM), etoposide (10 µM), 13-cis-retinoic acid (10 µM) and docetaxel (0.01 µM). Data represented as mean ± SD; statistical test (**D**) Representative immunoblots showing reduced RD3 protein levels at 36h and 48h after treatment with vincristine, doxorubicin, cisplatin, etoposide, 13-cis-retinoic acid and docetaxel. Treatment with 13-cis-retinoic acid or with docetaxel inflicted a cell-specific response with a significant loss in SH-SY5Y and only a marginal difference in other two cells investigated.


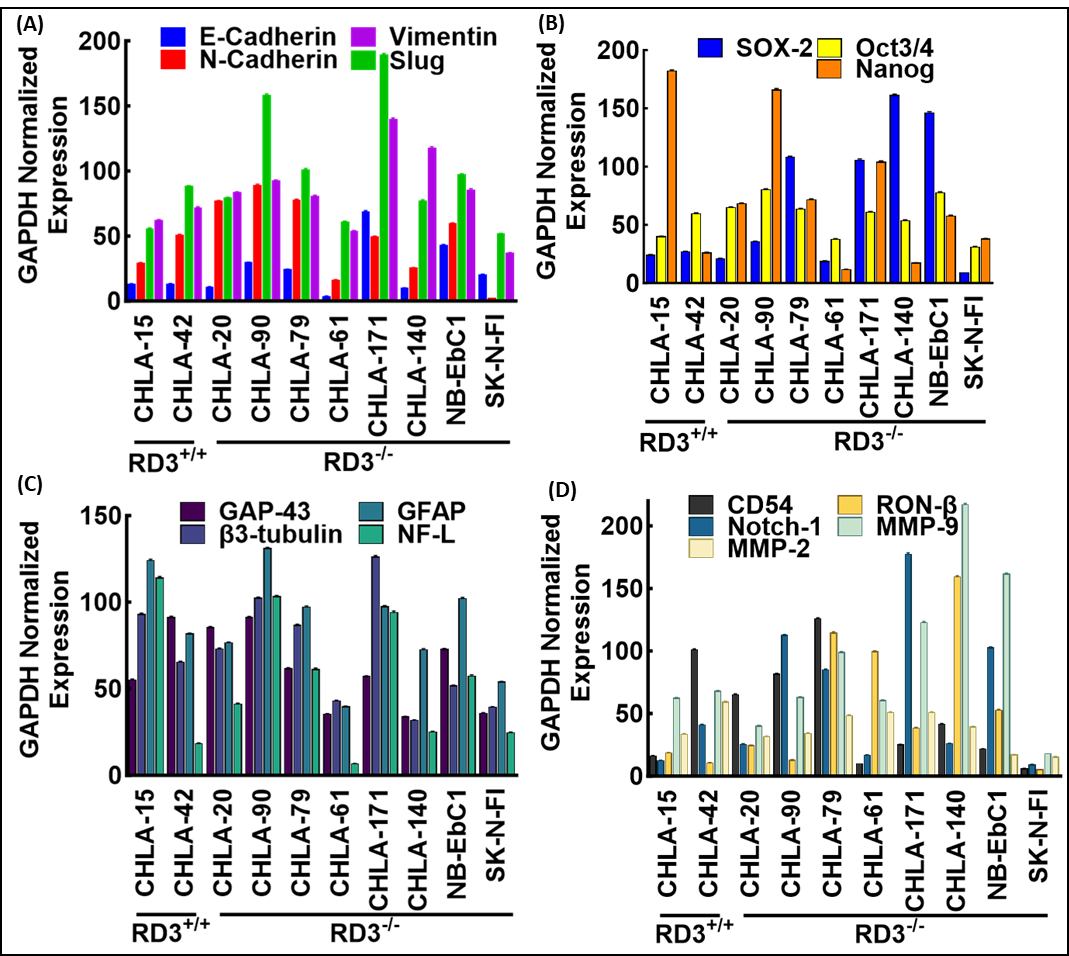


**Figure S2:** WB quantitation reveal RD3-status associated molecular reprogramming across a panel of NB cell lines derived from Dx (RD3^+/+^; CHLA-15, CHLA-42) and PD (RD3^-/-^; CHLA-20, CHLA-90, CHLA-140, CHLA-79, CHLA-61, CHLA-171, NB-Eb-C1, SK-NFI) in **(A) EMT**; PD-RD3^-/-^ cells exhibited reduced epithelial phenotype with concurrent elevation in N-Cadherin, Vimentin and Slug, consistent with mesenchymal shift and enhanced plasticity. **(B) Differentiation**; Dx-RD3^+/+^ showed higher expression of GFAP, GAP43, β-3-Tubulin and NFH while PD-RD3^-/-^ models displayed marked reduction suggesting dedifferentiation and poor lineage commitment. **(C) Pluripotency triad**; RD3-loss in PD increased the expression of all three Sox2, Nanog, Oct3/4 markers reflecting reactivation of core circuitry and acquisition of self-renewal and stem-like traits. **(D) Tumor dissemination**; protein implicated in metastatic competency MMP9, MMP2, RONβ, NOTCH1, and CD54 showed marked increase in PD cells characterized by therapy pressure acquired RD3-loss when compared to RD3-expressing Dx cells.


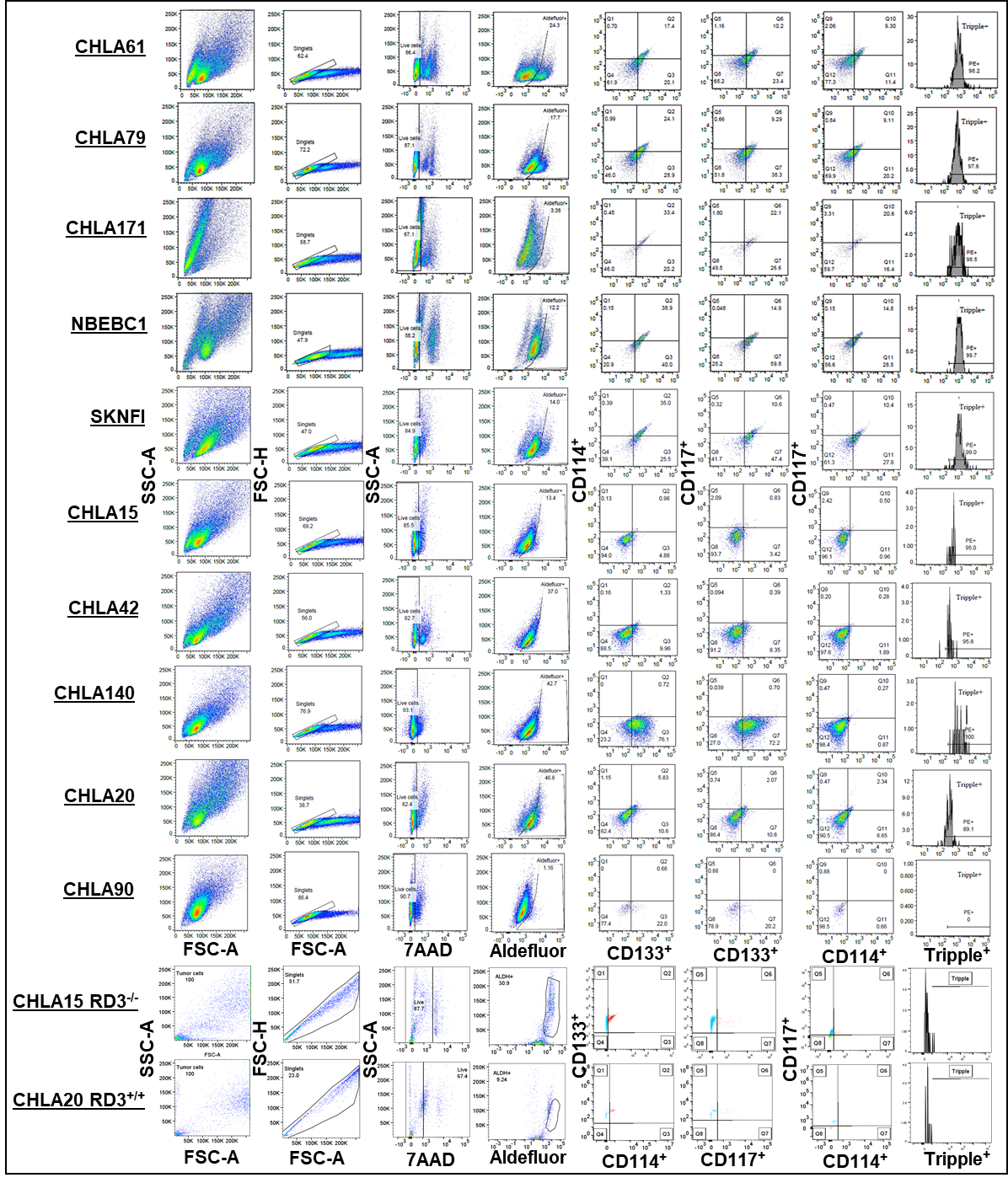


**Figure S3:** A hierarchical gating strategy that enabled precise isolation of highly enriched CSC subsets in NB. The workflow integrates stringent inclusion-exclusion criteria including selection of single viable cells followed by functional profiling to ensure high-purity recovery of biologically relevant CSCs. FSC vs. SSC parameters were used to include total cellular events and exclude debris. This ensures selection of intact cells for downstream analysis. FSC-A vs. FSC-H gating excluded doublets and cellular aggregates for accurate sorting fidelity. Live cells were further identified using a viability dye (7AAD) allowing us to exclude dead and apoptotic cells followed by identification of ALDH^+^ side population in NB. ALDH^+^ cells were further screened for the expression of canonical CSC surface markers CD133, CD114 and CD117 and sequentially sorted for single- (CD133^+^, CD114^+^, CD117^+^), double- (CD133^+^CD114^+^, CD133^+^CD117^+^, CD114^+^CD117^+^) and triple (CD133^+^CD114^+^CD117^+^) positive CSC subsets. FSC=Forward scatter; SSC=Side scatter; FSC-A=Forward scatter area; FSC-H=Forward scatter height.


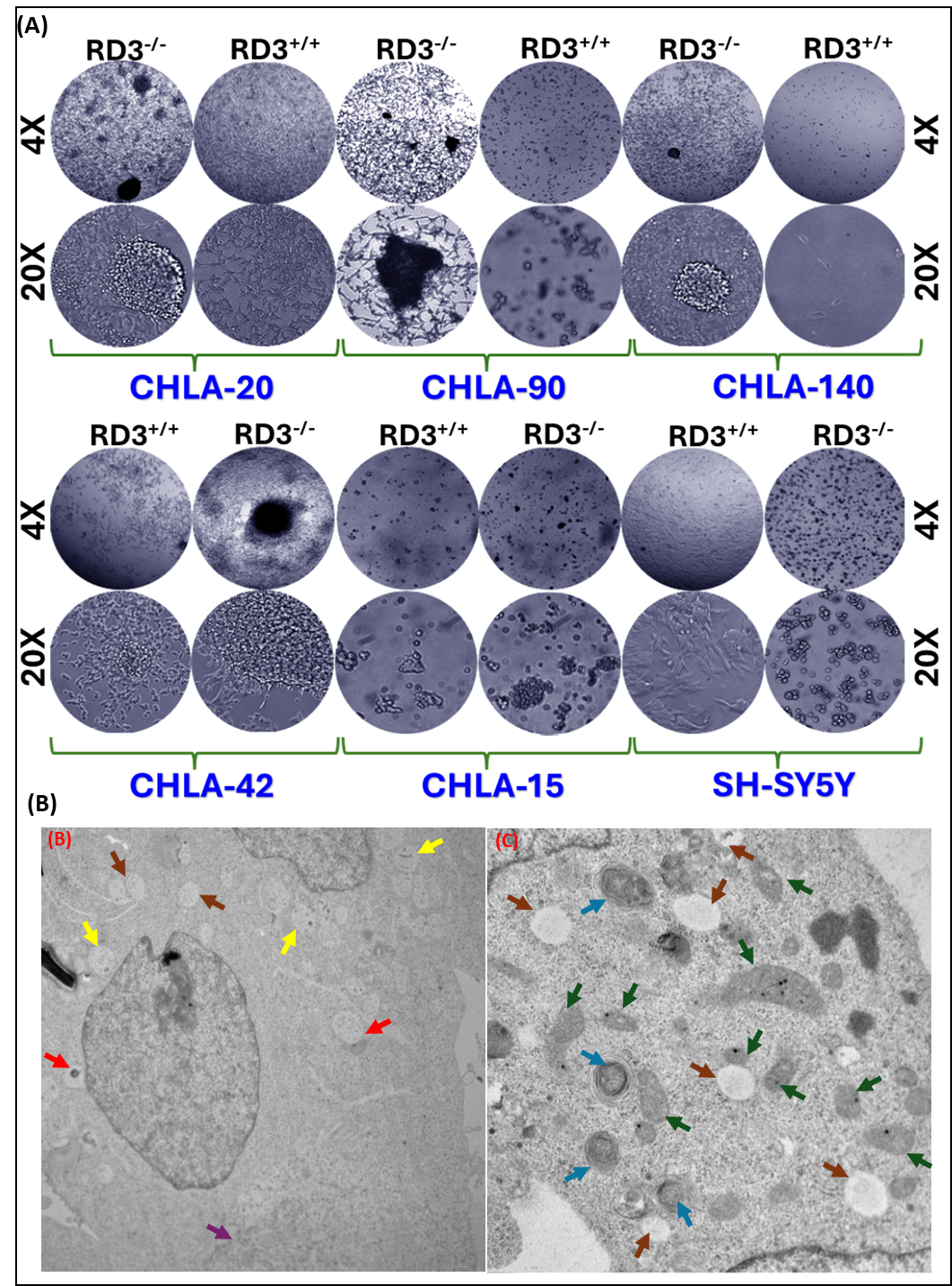


**Figure S4:** **(A)** morphometric profiling using phase contrast imaging of parental clones (n=6) and RD3-reverse engineered clones (n=6) reveals distinct RD3 -dependent architectural phenotypic shift in NB. PD-RD3^-/-^ models CHLA-20, CHLA-90, CHLA-140 exhibited growth of anchorage-independent cellular clusters with organized tumorosphere formation while stable re-expression of RD3 in these systems (CHLA-20 RD3^+/+^, CHLA-90 RD3^+/+^, CHLA-140 RD3^+/+^) completely abrogated tumorosphere formation and promoted monolayer spreading with neurite projections. On the flip side, RD3^+/+^ models displayed attached, polarized morphology while RD3-silencing in these cell lines (CHLA-15 RD3^-/-^, CHLA-42 RD3^-/-^, SH-SY5Y RD3^-/-^) led to reversal of apico-basal polarity promoting tumorosphere forming behavior. These morphometric transitions reflect a continuum of RD3 governed cytoskeletal reorganization and cellular architecture. **(B)** High-resolution TEM imaging of RD3^+/+^ and RD3^-/-^ NB systems highlight RD3-dependent changes in organelle distribution, cytoplasmic density, membrane and nuclear integrity. RD3^-/-^ cells exhibited smooth homogenous cytoplasm rich in mitochondria and lipid droplets while RD3^+/+^ cells were observed with irregular membrane and granular cytoplasm with low mitochondrial abundance suggestive of stress response. Colored arrows denote key features including mitochondrial morphology (dark green), vesicular trafficking (yellow), lipid moieties (brown), lipophagy (light blue), lysosomal contents (purple) and autophagosome (red). Magnification 5000x.

**
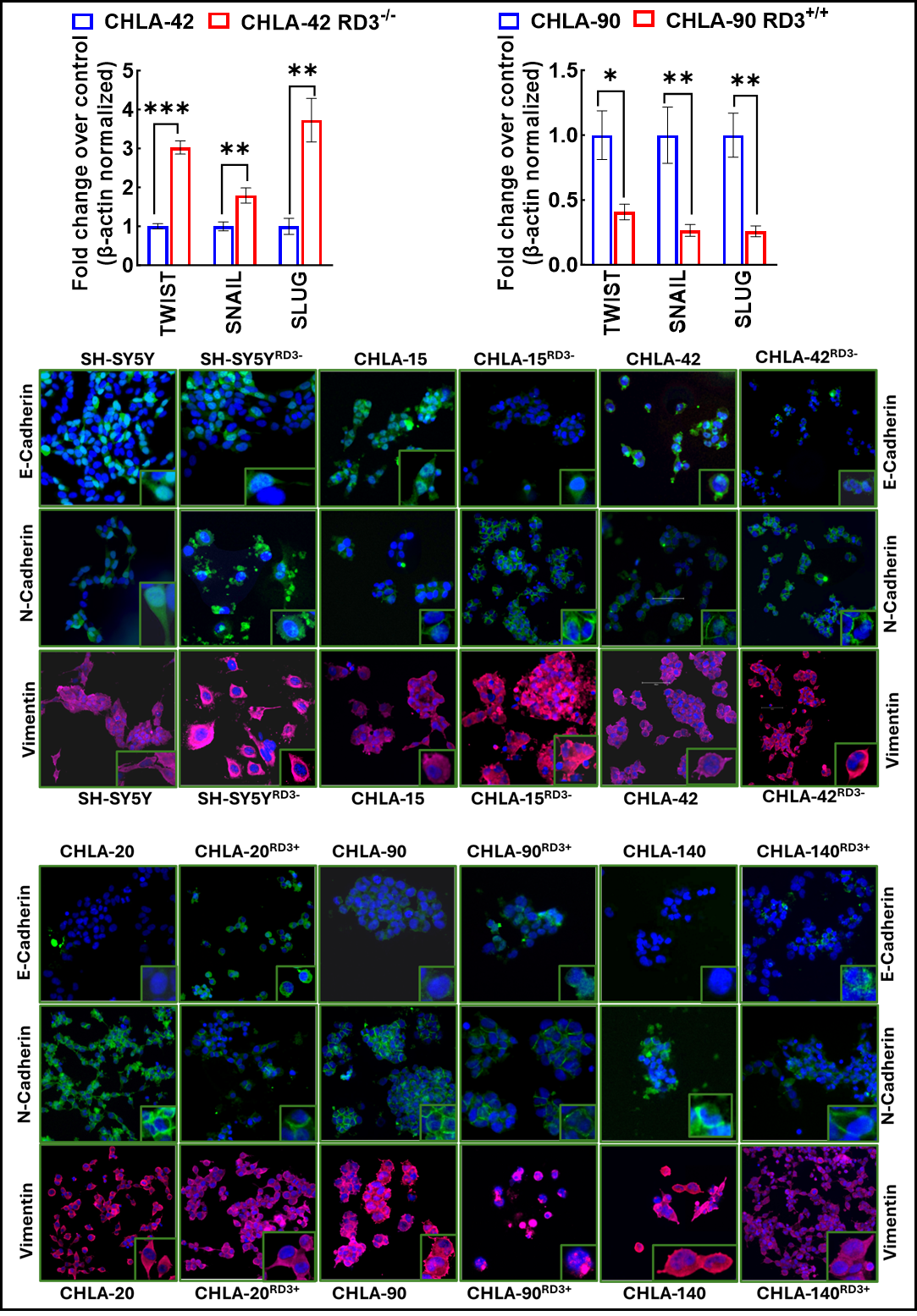
**

**Figure S5: (A) Epithelial-mesenchymal transition;** ***Top panel*** – transcriptional profiling of EMT-TFs Twist, Snail and Slug revealed significant elevation in RD3-/- clones when compared to RD3-reinstated clone, reinforcing the activation of EMT regulatory circuitry upon RD3-loss. Data represented as fold change normalized to β-actin (mean ± SEM), statistical analysis was performed using t-test. ***Bottom panel*** - Representative multiplex-IF microphotographs of a panel of NB parental (CHLA-15, CHLA-42, SH-SY5Y, CHLA-20, CHLA-90, CHLA-140) and RD3 reverse engineered (CHLA-15 RD3^-/-^, CHLA-42 RD3^-/-^, SH-SY5Y RD3^-/-^, CHLA-20 RD3^+/+^, CHLA-90 RD3^+/+^, CHLA-140 RD3^+/+^) clones depicting expression and localization of canonical EMT initiators – E-Cadherin, N-Cadherin and Vimentin. RD3-silenced and PD-RD3^-/-^ systems exhibited reduced E-cadherin alongside elevation in N-cadherin and Vimentin indicative of mesenchymal and cytoskeletal reprogramming. Contrarily, RD3-reinstated and Dx-RD3^+/+^ showed pronounced epithelial morphology with membrane localized E-cadherin.

**
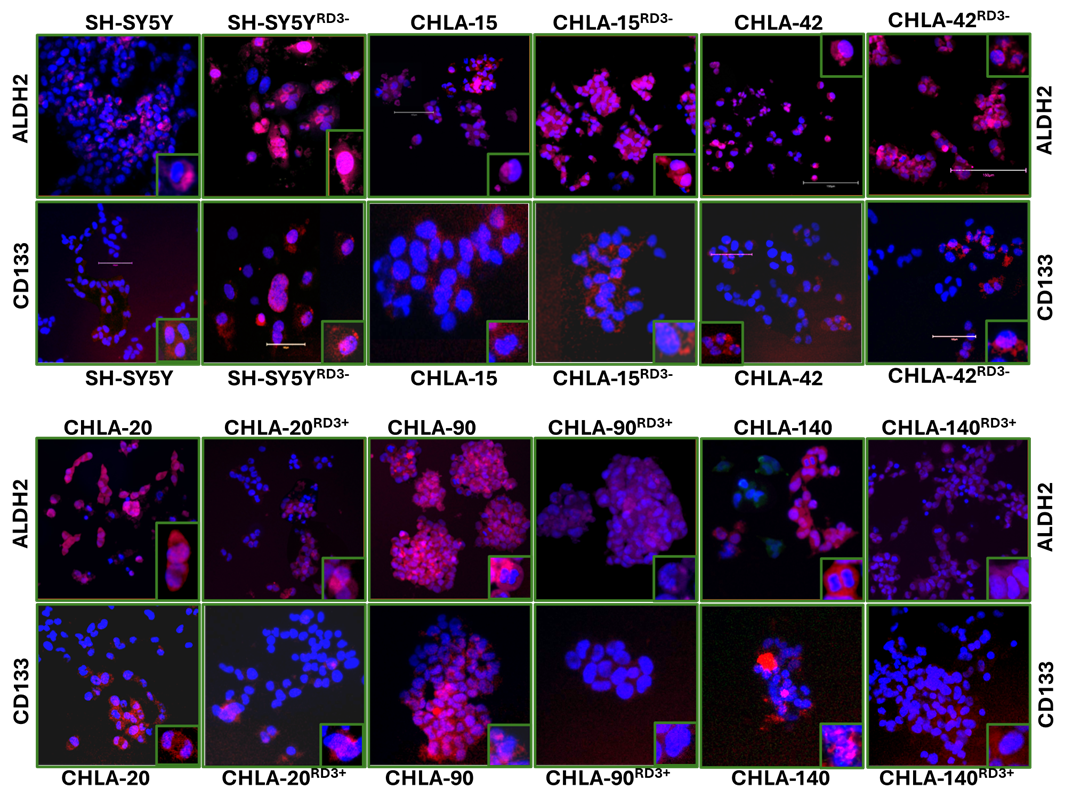
**

**Figure S5:** Representative multiplex-IF microphotographs of a panel of NB parental (CHLA-15, CHLA-42, SH-SY5Y, CHLA-20, CHLA-90, CHLA-140) and RD3 reverse engineered (CHLA-15 RD3^-/-^, CHLA-42 RD3^-/-^, SH-SY5Y RD3^-/-^, CHLA-20 RD3^+/+^, CHLA-90 RD3^+/+^, CHLA-140 RD3^+/+^) clones **(B)** Stemness; revealed dynamic shift in stem-like phenotype with RD3-null cells exhibiting enriched stem-like (CD133, ALDH2) phenotype compared to RD3-expressing and RD3-reinstated clones.

**
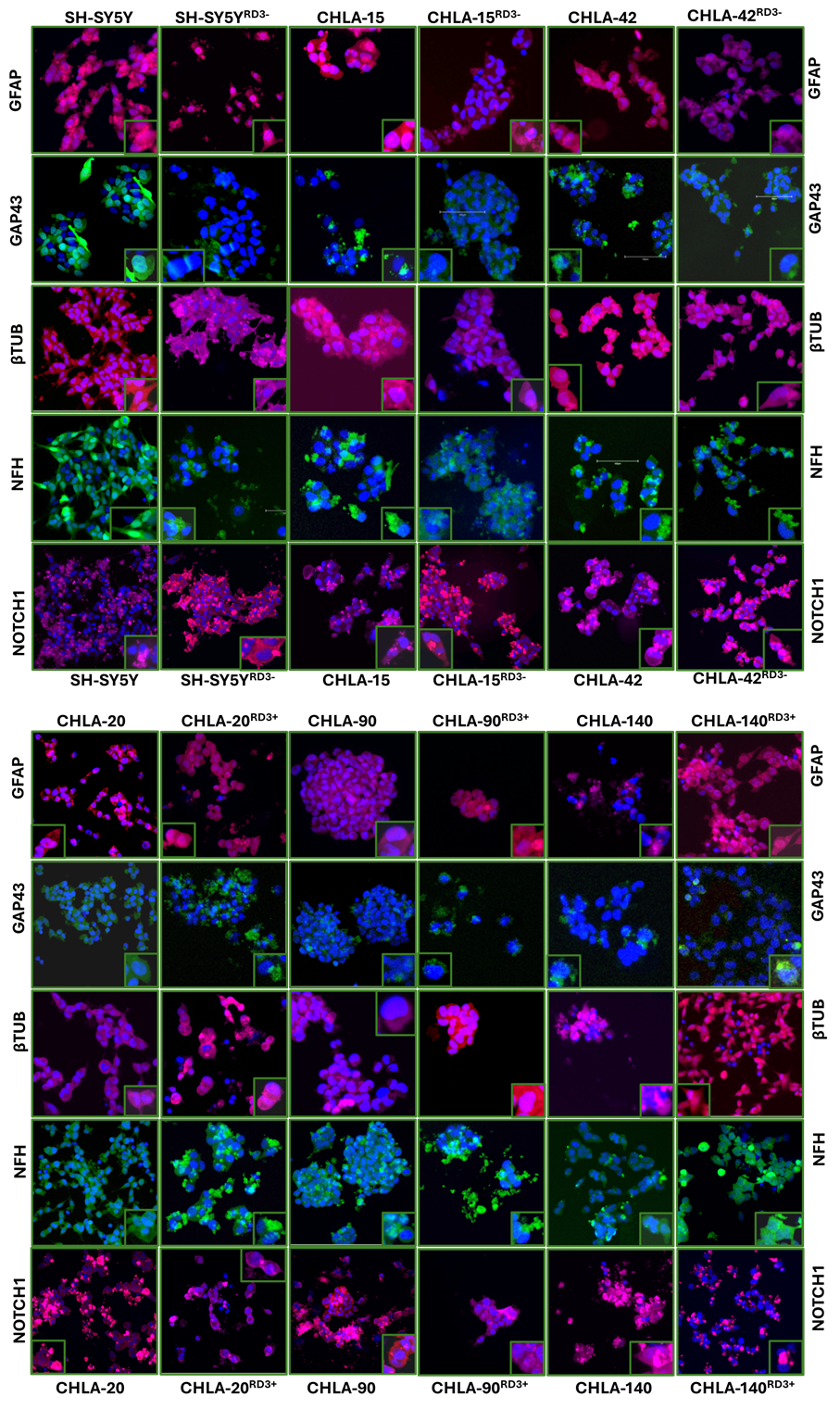
**

**Figure S5:** Representative multiplex-IF microphotographs of a panel of NB parental (CHLA-15, CHLA-42, SH-SY5Y, CHLA-20, CHLA-90, CHLA-140) and RD3 reverse engineered (CHLA-15 RD3^-/-^, CHLA-42 RD3^-/-^, SH-SY5Y RD3^-/-^, CHLA-20 RD3^+/+^, CHLA-90 RD3^+/+^, CHLA-140 RD3^+/+^) clones **(C) Differentiation;** stained for maturation markers (GFAP, GAP43, β-3-Tubulin, NFH) and stemness inducer (NOTCH1) showed that RD3-expressing and reinstated systems displayed robust expression of differentiation markers with polarized neurite outgrowth and lineage fidelity while RD3-deficeint and silenced systems exhibited undifferentiated phenotype consistent with elevated levels of NOTCH1.

**
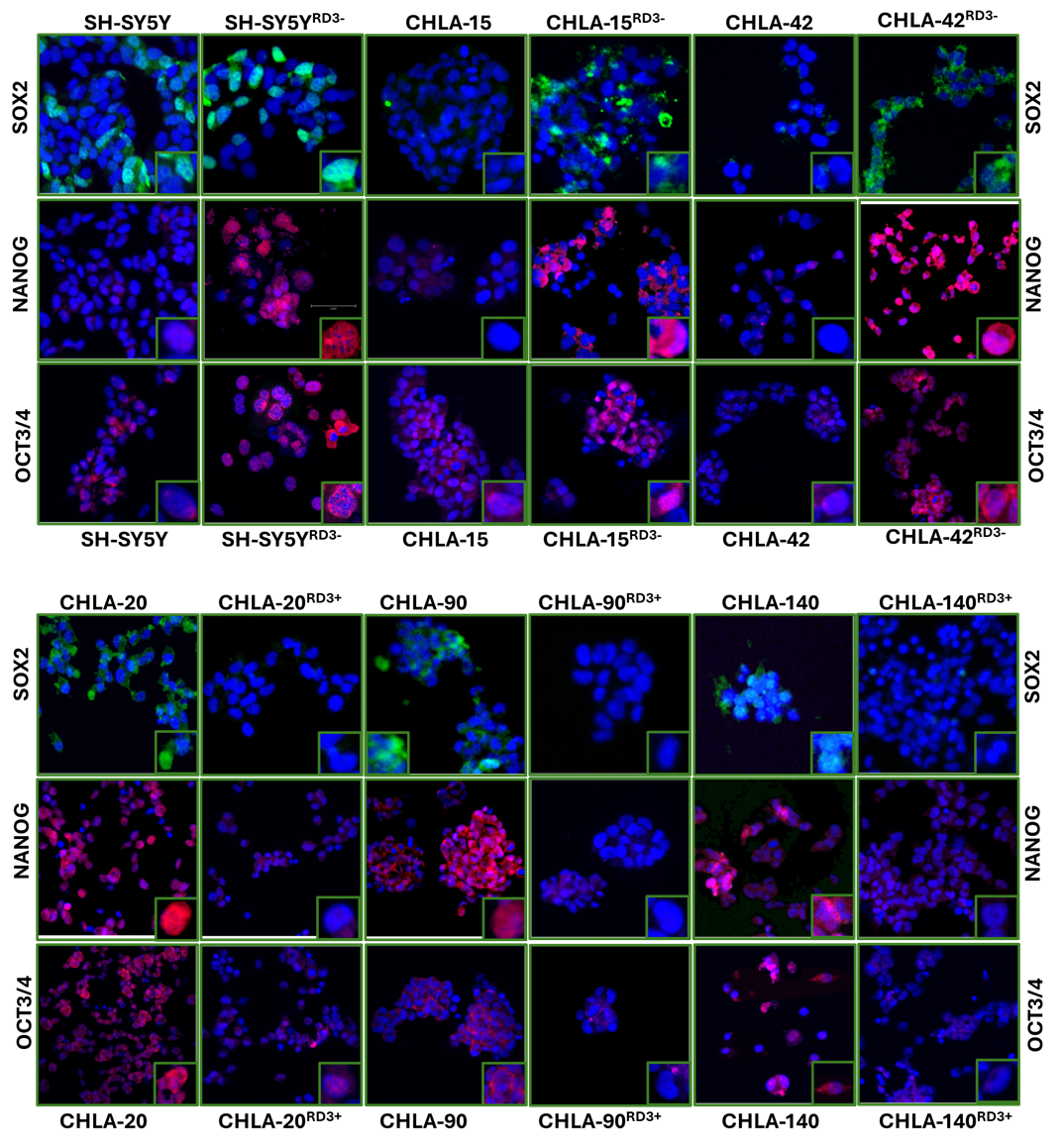
**

**Figure S5:** Representative multiplex-IF microphotographs of a panel of NB parental (CHLA-15, CHLA-42, SH-SY5Y, CHLA-20, CHLA-90, CHLA-140) and RD3 reverse engineered (CHLA-15 RD3^-/-^, CHLA-42 RD3^-/-^, SH-SY5Y RD3^-/-^, CHLA-20 RD3^+/+^, CHLA-90 RD3^+/+^, CHLA-140 RD3^+/+^) clones **(D) Pluripotency maintenance;** showed heightened expression and nuclear localization of core pluripotency components SOX2, NANOG, OCT3/4 in RD3-silenced and PD-RD3^-/-^ consistent with increased plasticity and dedifferentiation. In contrast RD3-reistated and Dx-RD3^+/+^ systems showed attenuated expression of pluripotency markers with reduced plasticity


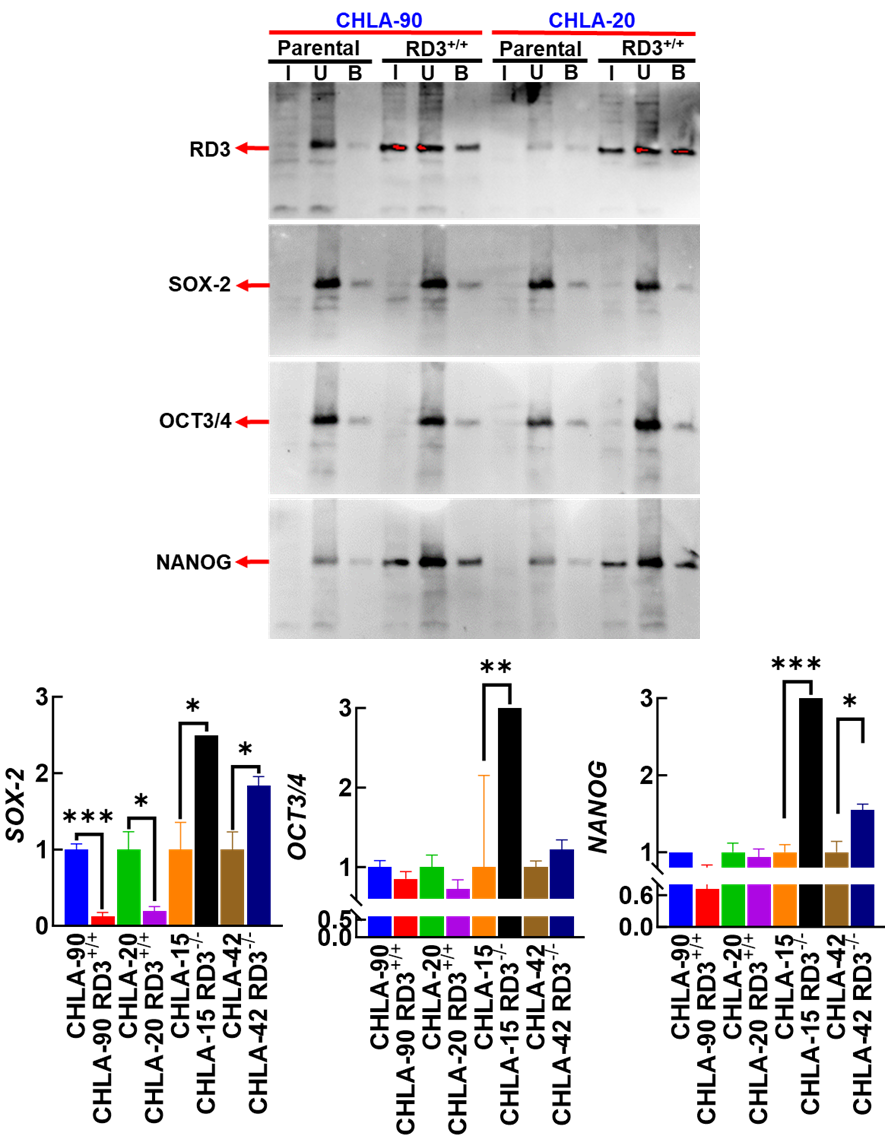


**Figure S5: RD3 binds to SOX-2, OCT3/4, NANOG and regulates their promoter activity (E)** ***Top panel*** - Representative blots from RD3-pull down probed for SOX-2, OCT3/4 and NANOG RD3-null (CHLA-20, CHLA-90) and their RD3-reinstated counterparts (CHLA-20 RD3^+/+^, CHLA-90 RD3^+/+^). Co-IP showed selective strong interaction with core circuitry pluripotency maintenance factors (SOX-2, OCT3/4, NANOG). ***Bottom panel*** - ChIP-qPCR was performed to assess the RD3-promoter occupancy at SOX-2, NANOG, OCT3/4 promoter regions in RD3^+/+^, RD3-null, RD3-silenced and RD3-reinstated clones. RD3-silencing significantly increases promoter occupancy at *SOX-2* and markedly promotes binding to both *OCT3/4* and *NANOG* promoters. Conversely, RD3 re-expression profoundly reduces promoter binding to these pluripotency drivers. Data is represented as relative quantity (mean ± SEM) normalized to respective controls; statistical analysis was performed using t-test. (I, input; U, unbound; B, bound fractions).


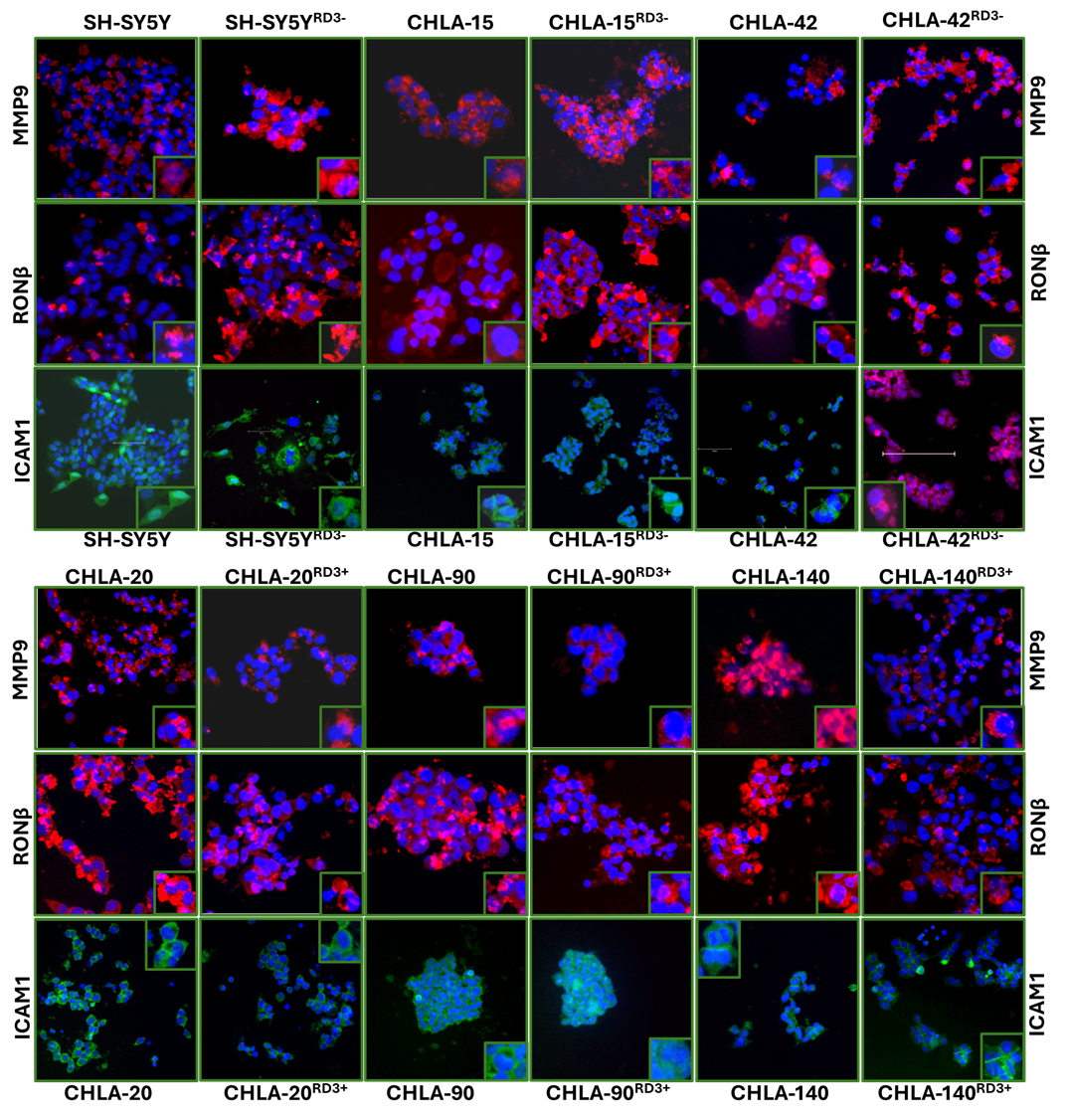


**Figure S5:** Representative multiplex-IF microphotographs of a panel of NB parental (CHLA-15, CHLA-42, SH-SY5Y, CHLA-20, CHLA-90, CHLA-140) and RD3 reverse engineered (CHLA-15 RD3^-/-^, CHLA-42 RD3^-/-^, SH-SY5Y RD3^-/-^, CHLA-20 RD3^+/+^, CHLA-90 RD3^+/+^, CHLA-140 RD3^+/+^) clones **(F) Invasion and Migration** showed enhanced metastatic competence in RD3-deficient and PD-RD3^-/-^ systems coherent with increased MMP9, RONβ and ICAM1 promoting matrix degradation and detachment. In contrast, DX-RD3^+/+^ and RD3-reexpressed systems reduced the expression of metastasis inducers reflecting a stabilized cellular architecture.

**
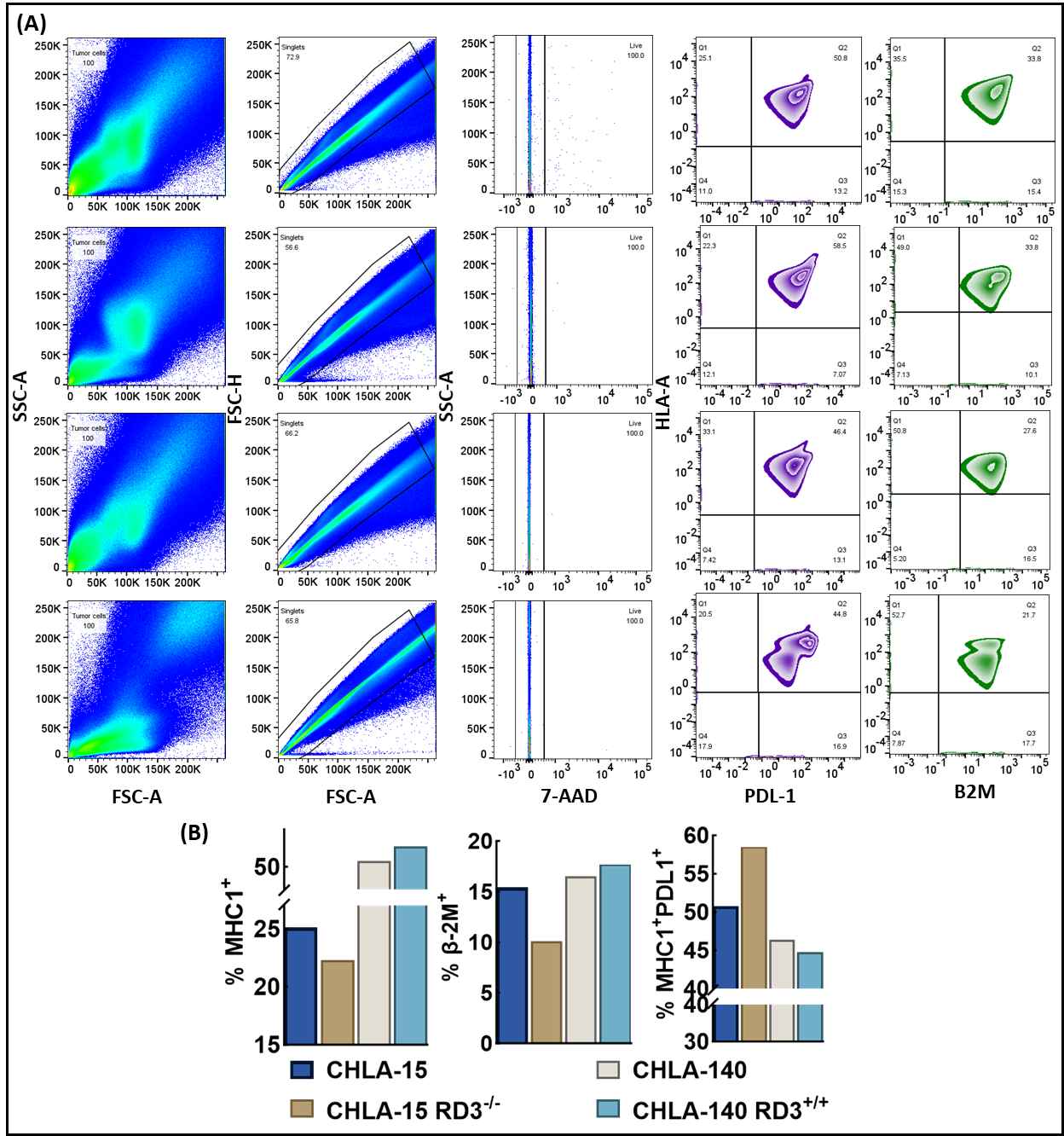
**

**Figure S6:** **MHC1, PDL1, β-2M Co-expression on NB clones (A)** FACS gating strategy for assessing surface expression of immune markers in NB parental (CHLA-15, CHLA-140) and RD3 reverse engineered (CHLA-15 RD3^-/-^ and CHLA-140 RD3^+/+^) clones. Initial gates applied on FSC-A vs SSC-A to exclude debris, followed by FSC-H vs FSC-A to select singlets. Viability was confirmed using 7-AAD exclusion. Subsequent biaxial plots show gating for double positives MHC1^+^B2M^+^ and MHC1^+^PDL1^+^. **(B)** Bar graphs show percentage of T_u_ positive for MHC1, β-2M and co-expression of MHC1^+^ PDL1^+^ across parental clones (CHLA-15, CHLA-140) and RD3 reverse engineered clones (CHLA-15 RD3^-/-^ and CHLA-140 RD3^+/+^). The results indicate T_u_ RD3-status relevant surface expression of MHC1, β-2M and MHC1+ PDL1+ those facilitate tumor immunogenicity.

**
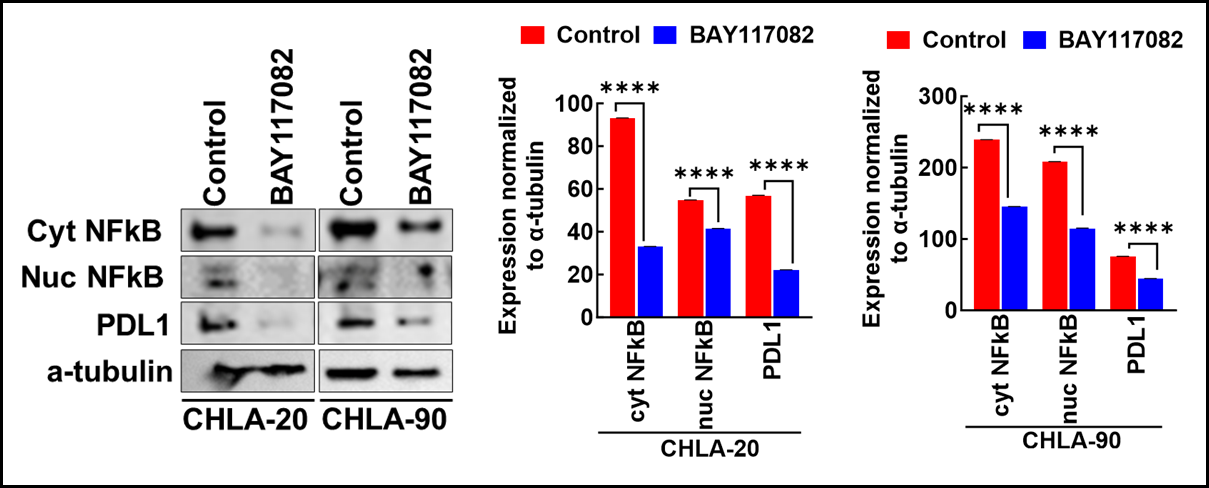
**

**Figure S7:** Representative immunoblots showing expression levels of cytoplasmic and nuclear NFκB p65 and cytoplasmic PDL-1 expression in RD3-deficient systems CHLA-20 and CHLA-90 exposed with and without NF-κB inhibitor BAY11-7082. α-tubulin is used as the loading controls. Histograms from the band intensity analysis showing: significantly strong nuclear and cytoplasmic localization of NFκB in RD3-deficient PD cells, along with significantly elevated PDL-1 expression in cytoplasm; substantial reduction in PDL-1 expression when NF-κB (p65) activation in these cells are blocked with Bay 11-7082, suggesting that RD3-loss promotes immune evasion through NF-κB-driven PDL1 induction in NB. Data represented as fold change normalized to α-tubulin (mean ± SEM), statistical analysis was performed using t-test.

**
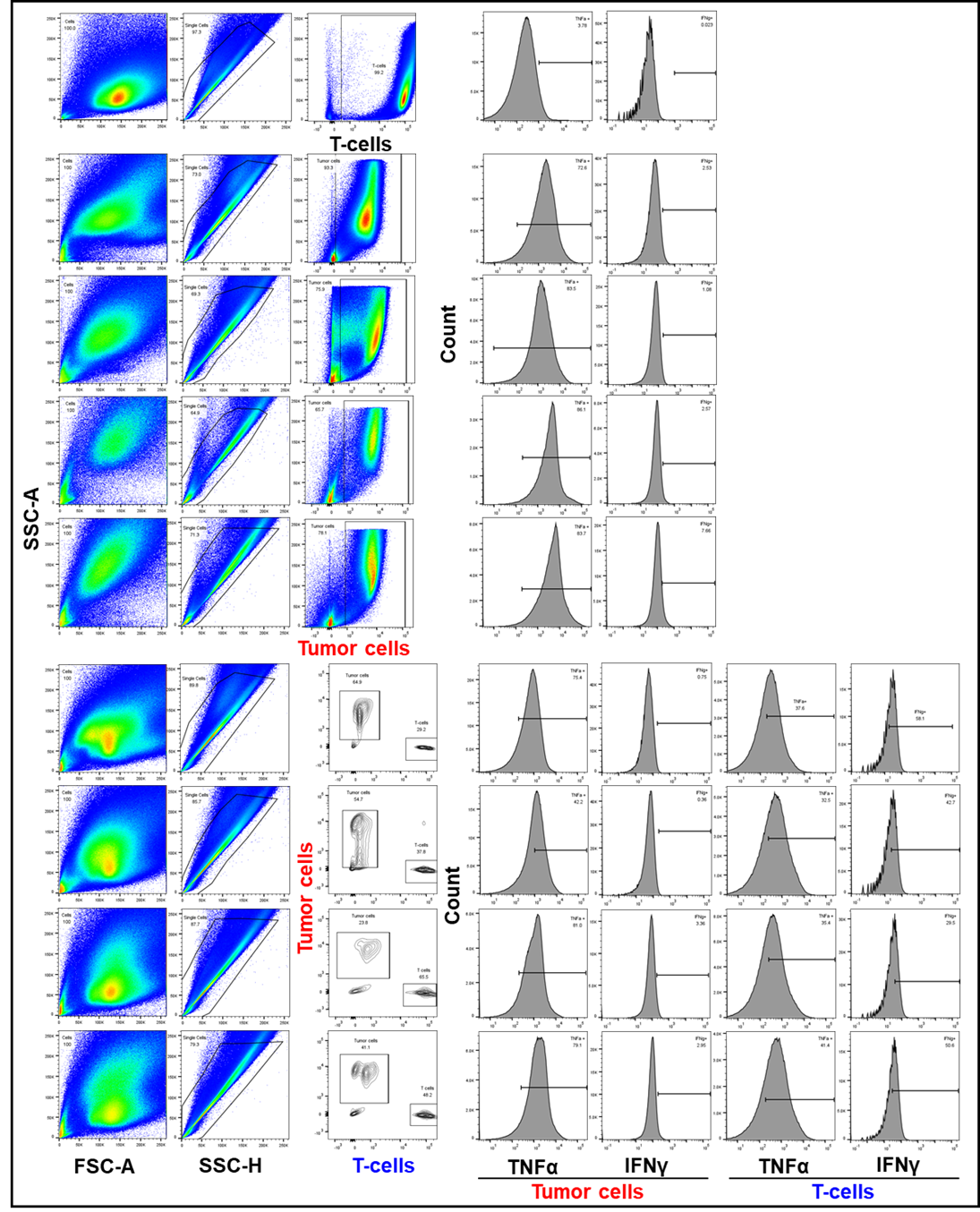
 Figure S8:** FACS gating strategy for cytokine secretion assay performed in NB parental (CHLA-15, CHLA-90) and RD3 reverse engineered (CHLA-15 RD3^-/-^ and CHLA-90 RD3^+/+^) clones. **(A)** Sequential inclusion-exclusion criteria were applied to precisely identify cytokine secreting Tc and Tu subsets. Debris, doublets and aggregates were excluded using SSC-A vs FSC-A/SSC-H parameters. Viable cells were analyzed for cytokine secreting (TNFα and IFNγ) populations in unstimulated and stimulated conditions.


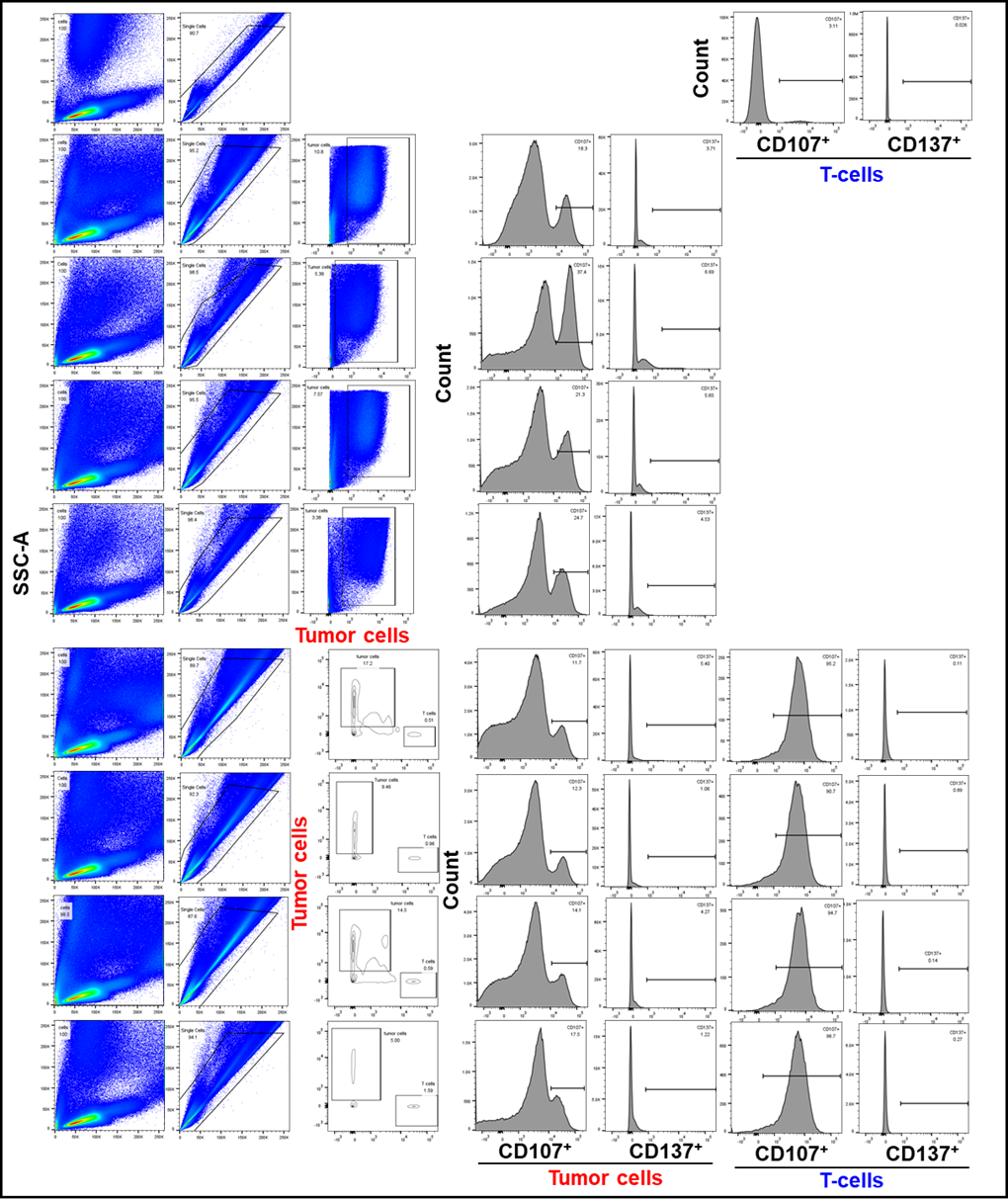


**Figure S8:** FACS gating strategy for tumor reactivity assay functional immune assays performed in NB parental (CHLA-15, CHLA-90) and RD3 reverse engineered (CHLA-15 RD3^-/-^ and CHLA-90 RD3^+/+^) clones. **(B)** Systematic workflow applied as described earlier followed by screening for tumor reactive T_c_. T_c_ sub-population expressing T_c_ activation (CD137) and degranulation (CD107a) marker under unstimulated and stimulated conditions were identified.


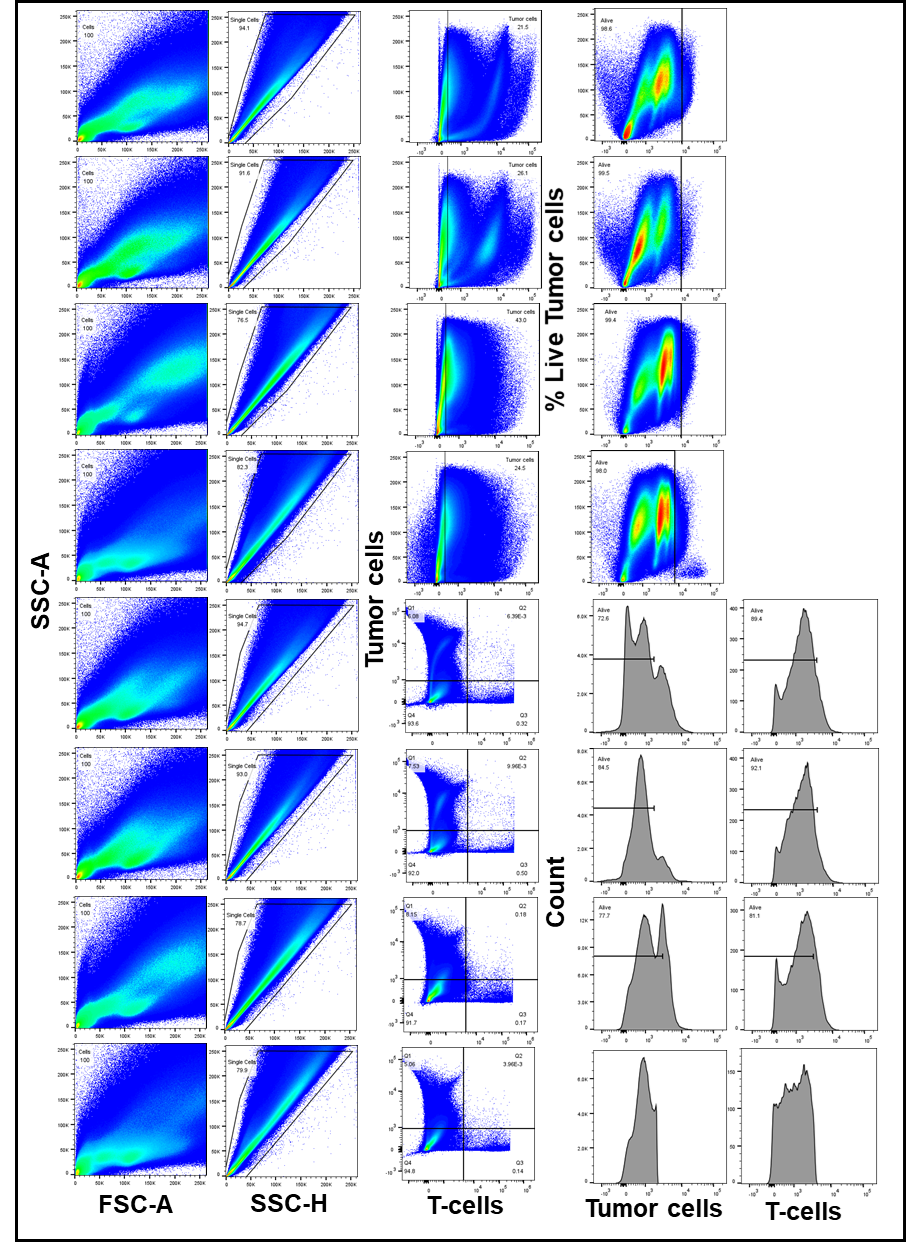


**Figure S8:** FACS gating strategy for tumor killing assay performed in NB parental (CHLA-15, CHLA-90) and RD3 reverse engineered (CHLA-15 RD3-/- and CHLA-90 RD3+/+) clones. **(C)** Cells were gated to exclude debris and doublets using FSC/SSC parameters. T_u_ was then assessed for caspase activity using fluorogenic caspase-3 substrate, enabling quantification of apoptosis following CoC with effector T_c_ under unstimulated and stimulated conditions.
